# Multifunctional kojic acid-tetrahydroisoquinoline hybrids: Synthesis, tyrosinase inhibition, and applications in the anti-browning of fresh-cut mushrooms

**DOI:** 10.1016/j.crfs.2025.101248

**Published:** 2025-11-16

**Authors:** Min Lv, Longkang Cao, Yulu Ding, Shan Gao, Yinxin Wu, Rong Li, Wenjian Tang, Lili Zhu

**Affiliations:** School of Pharmacy, Inflammation and Immune Mediated Diseases Laboratory of Anhui Province, Anhui Medical University, Hefei, 230032, China

**Keywords:** Tetrahydroisoquinoline, Antimicrobial activity, Anti-browning, Fresh cut mushrooms, Food preservation

## Abstract

Novel kojic acid-tetrahydroisoquinoline hybrids (**4a–4p**) were designed and synthesized to address the need for safe tyrosinase inhibitors in food preservation. Compound **4c** emerged as the most potent inhibitor, exhibiting 40-fold greater activity (IC_50_ = 1.27 μM) than kojic acid. Kinetic studies identified **4c** as a mixed-type inhibitor (*K*_i_ = 1.48 μM, *K*_is_ = 15.35 μM), with molecular docking revealing strong binding to tyrosinase's active site *via* copper chelation and hydrophobic interactions. Safety evaluations in zebrafish embryos and mammalian cell lines confirmed minimal cytotoxicity. Furthermore, **4c** showed broad-spectrum antimicrobial activity against *E. coli*, *S. aureus* and *B. cinerea*. In practical applications, **4c** effectively suppressed enzymatic browning in fresh-cut mushrooms. These results underscore **4c** as a promising candidate for multifunctional food preservatives, combining anti-browning efficacy, microbial inhibition, and biocompatibility. This study provides a strategic framework for developing hybrid inhibitors to enhance food quality and safety.

## Highlights

• Sixteen kojic acid-tetrahydroisoquinoline hybrids were designed and synthesized.
• **4c** exhibited 40 times more potent tyrosinase inhibitory activity than kojic acid.
• **4c** had benign safety profiles and significant antimicrobial effect.
• **4c** could effectively retard the browning of fresh-cut mushrooms.

## Graphical abstract

**Figure:**
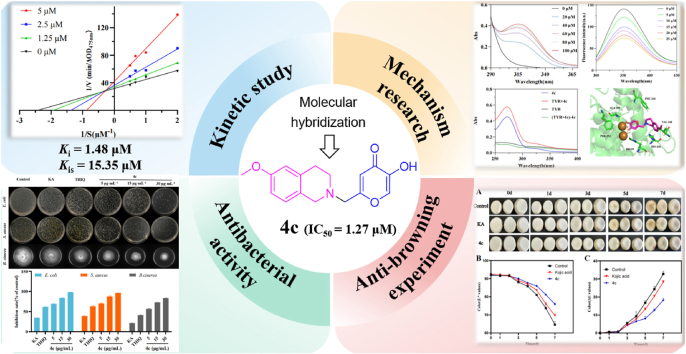

## 1 Introduction

Tyrosinase (TYR) has been identified as a crucial regulatory factor in mammalian systems, mediating two pivotal biological processes: plant-derived enzymatic browning, commonly observed in fruits, fungi, and vegetables, and abnormal melanin deposition in the skin (Zolghadri et al., 2019). Phenolic compounds in fruits and vegetables were found to undergo oxidation catalyzed by tyrosinase, leading to the formation of brown pigments. This process is identified as the primary enzymatic browning reaction (Peng et al., 2022). The enzymatic mechanism is characterized by the hydroxylation of L-tyrosine to L-3,4-dihydroxyphenylalanine (L-DOPA), which is subsequently oxidized to dopaquinone, ultimately leading to the biosynthesis of melanin (Li et al., 2021). However, enzymatic browning is often induced during the postharvest processing or storage of fresh produce, leading to significant deterioration in both appearance and flavor, a decline in nutritional value, and a reduction in marketability. Consequently, inhibition of tyrosinase activity has been recognized as a crucial strategy in food preservation (Zhang et al., 2024).

Kojic acid (KA, **1**) has been recognized as a representative tyrosinase inhibitor with prominent inhibitory activity (Patamia et al., 2024; Pillaiyar et al., 2017). Additionally, kojic acid has been reported to exhibit diverse biological properties, including antimicrobial (Brtko, 2022; Patamia et al., 2023), anti-inflammatory (Aytemir and Calis, 2010), antiviral (Saeedi et al., 2019). However, instability, susceptibility to degradation, and possible adverse reactions of KA have significantly restricted its application in the food industry (Burdock et al., 2001). Consequently, studies on kojic acid derivatives were used to develop more stable and potent tyrosinase inhibitors. For instance, kojic acid-aromatic aldehyde **6j** with 3-fluorine and 4-aldehyde had a good safety profile and potent anti-tyrosinase activity, exhibiting significant anti-browning effects in apples (Peng et al., 2023).

There are more than 3000 natural products possess or derive from the tetrahydroisoquinoline (THIQ) moiety, which plays an important role in medicinal chemistry due to its wide range of pharmacological properties (Abe et al., 2005; Gangapuram et al., 2021; Wang et al., 2017). The main active component of plant essential oils, apigenin, has broad-spectrum antibacterial activity and potential applications in food preservation (Gao et al., 2025). Currently, food packaging materials are widely used in the field of food preservation. Bio-inspired self-healing materials represent a shift from static to dynamic food packaging, offering intelligent, resilient, and sustainable preservation for the future (Feng et al., 2025a, Feng et al., 2025b; Wang et al., 2024).

**Fig. 1:**
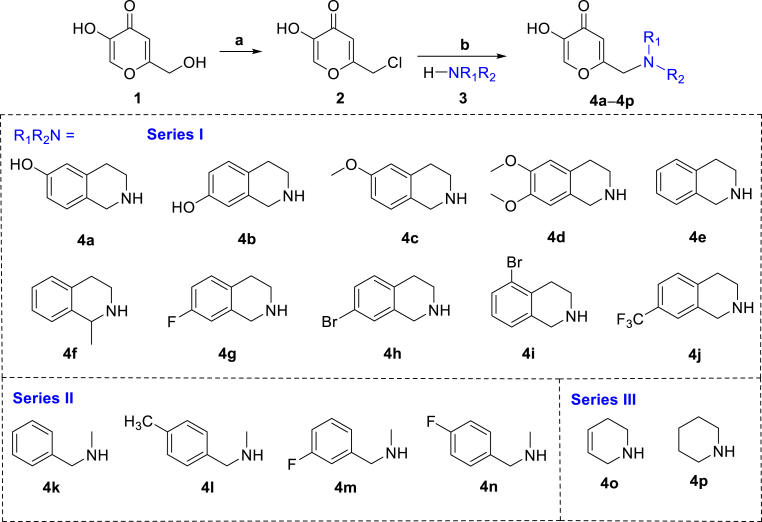
Synthesis of kojic acid-tetrahydroisoquinoline hybrids. (a) SOCl_2_, 0 °C, 3 h; (b) Et_3_N, DMSO, RT.

It is an effective strategy to hybridize two or more active skeletons of natural products into potential bioactive molecules. In this work, it is reported for the first time that the tetrahydroisoquinoline as privileged scaffold was incorporated into kojic acid into a single molecule, and novel kojic acid-tetrahydroisoquinoline hybrids were evaluated for their tyrosinase inhibitory activities (Fig. S1 and Fig. 1). The most active compound **4c** was selected to investigate its anti-browning and antibacterial effects along with safety profiles. Compared to natural systems, compound **4c** demonstrated significant improvements in stability, safety, and enzyme-inhibiting activity. This work will provide a theoretical foundation for developing safe and highly effective tyrosinase inhibitors.

## 2 Materials and methods

### 2.1 Reagents and equipment

TYR, L-tyrosine, and L-DOPA were purchased from Shanghai Macklin Biochemical Co., Ltd. (China), and kojic acid was purchased from Shanghai Aladdin Biochemical Technology Co., Ltd. (China). Tetrahydroisoquinolines with various substituent for and other raw materials for synthesis were obtained from Energy Chemicals Inc. (China). Unless otherwise noted, all other reagents are analytical grade reagents. Fresh button mushrooms were procured from local markets for anti-browning experiments. The zebrafish were provided by the research group of Sujuan Zhao at the School of Public Health, Anhui Medical University. NR60CP colorimetric spectrophotometer (Shenzhen, China). High-resolution mass spectra (HRMS) were acquired using an Agilent 1260–6221 TOF mass spectrometer. ^1^H and ^13^C NMR spectra were obtained using a Bruker AV-600 or AV-151 MHz apparatus. UV and fluorescence spectra were obtained from UV-1800 ultraviolet spectrophotometer (Shimadzu Jiangsu, China) and Cary Eclipse fluorescence spectrophotometer (Beijing, China), respectively.

### 2.2 Synthesis of **4a‒4p**

*Synthesis of compound* ***2***: KA (**1**, 10 mmol) was dissolved in dichloromethane (50 mL) and placed under an ice bath. Thionyl chloride (5 mL) was slowly dropped with stirring, while thin-layer chromatography (TLC) was used to monitor the reaction progress for 2–3 h. Subsequently, the mixture was repeatedly diluted with CH_2_Cl_2_ and concentrated to a small volume. It was then extracted with EtOAc until the aqueous phase became neutral. The organic phase was collected, concentrated to induce crystallization, and subjected to vacuum filtration to obtain intermediate **2**.

*Synthesis of compounds* ***4a‒4p***: The variously substituted tetrahydroisoquinolines (2 mmol) and intermediate **2** (352 mg, 2.2 mmol) were dissolved in dimethyl sulfoxide (15 mL). Subsequently, triethylamine (0.5 mL) was added, and the reaction mixture was allowed to react overnight at room temperature. The reaction progress was monitored by TLC. Upon completion, the mixture was extracted with EtOAc, concentrated in the organic phase, and purified by column chromatography to afford the target products (**4a‒4p**).

### 2.3 In vitro tyrosinase inhibitory study

L-tyrosine and L-DOPA served as substrates to assess the inhibitory impact of hybrids **4a‒4p** on tyrosinase. Several adjustments were implemented in the experimental methodology (Shao et al., 2018). Both the test compounds and the positive control, kojic acid, were dissolved in DMSO to prepare solutions with varying concentrations. Each well of a 24-well plate was then filled with 100 μL of the compound solution and 800 μL of a 0.5 mM substrate solution. Following a 5-min incubation at 37 °C, 100 μL of tyrosinase solution (35 U/mL) was introduced to each well, and the OD value was immediately measured at 475 nm. In the control group, no test compounds were added, while both enzymes and substrates were present. For the blank group, PB was used as a substitute. All experiments were replicated three times to ensure accuracy. The inhibition rate (%) was determined using formula (1):

(1) $$Inhibition\;rate\;\left(\%\right)=\left(1-\Delta A_{\text{sample}}/\Delta A_{\text{control}}\right)\times 100\%\text{.}$$

### 2.4 Cell viability assay

The safety of this series of hybrids was evaluated using the CCK-8 assay (Yu et al., 2022). In this experiment, B16F10 (mouse melanoma cell line) and HaCaT (human keratinocyte cell line) were primarily used as representatives to investigate safety. Log-phase cells (1 × 10^4^ cells/well) were seeded into 96-well plates, then incubated at 37 °C for 24 h. The cells were then treated with solutions of tested compounds at concentrations of 0, 12.5, 25, 50, and 100 μM. After 24 h of the interaction, the mixed solutions were aspirated and discarded, and the 96-well plates were washed twice with PBS. Subsequently, 30 μL of 10 % CCK-8 solution was added. Two hours later, the absorbance values of the mixtures were recorded at a wavelength of 450 nm using a multifunctional enzyme microplate reader.

### 2.5 Acute toxicity studies in zebrafish embryos

The acute toxicity assay was conducted in accordance with the Guidelines of Organization for Economic Co-operation and Development (OECD) for the Testing of Chemicals, Fish Embryo Acute Toxicity (FET) Test, No. 236 (OECD, 2013). All zebrafish procedures in this study were approved by Anhui Medical University Experimental Animal Ethics Committee (Protocol No.: LLSC20241625). The zebrafish were maintained in accordance with the established protocols of the Zebrafish International Resource Center. Surviving zebrafish were humanely euthanized at the end of the study to prevent prolonged suffering. The euthanasia method involved immersion in a buffered solution of tricaine methanesulfonate at a concentration of 300 mg/L for at least 10 min, followed by confirmation of death through the cessation of gill movement and heartbeat. Carcasses were promptly frozen at −20 °C and disposed of through authorized biological waste channels.

Referring to published methods with certain modifications (Qian et al., 2018), 13–15 h post-fertilization (hpf) zebrafish embryos were placed in small dishes containing 5 mL of culture medium in this experiment. Different concentrations of **4c** (25, 50, 100 μM) and KA (100 μM) were added to the dishes, respectively, and the experiments were repeated three times. The survival rate was observed at 48 hpf. Embryo coagulation, heart rate cessation, absence of somite formation, and failure of tail separation are all defined as embryo death (Zhou et al., 2022).

### 2.6 Kinetic analysis

Based on the IC_50_, the most effective compound **4c** was selected for kinetic analysis. Reaction rates were determined using different concentrations of compounds (1.25, 2.5, and 5 μM) and L-DOPA solutions (0.5, 1, 1.5, and 2 mM). The inhibitory mechanism of **4c** against tyrosinase was elucidated *via* Lineweaver-Burk double reciprocal plot analysis, revealing its inhibition dynamics and inhibition constant.

### 2.7 Copper ions chelation

Ultraviolet–visible spectrophotometry serves as a crucial analytical technique for probing the coordination chemistry of cupric ions with chromophore-containing active compounds exhibiting distinct absorption bands. By observing shifts in the ultraviolet characteristic absorption peaks, it could be determined whether chelation occurred between the small molecules and copper ions (Shang et al., 2018). According to the literature method (T. Chen et al., 2020), CuSO_4_·5H_2_O was dissolved in distilled water to prepare copper ion solutions of varying concentrations, while **4c** was dissolved in methanol to obtain a solution with a concentration of 200 μM. The copper ion solutions were added to **4c** solution and incubated at room temperature for 30 min. Subsequently, the UV–vis absorption spectrum of the mixture was recorded at room temperature within the wavelength range of 200–600 nm using a UV–vis spectrophotometer.

### 2.8 Fluorescence spectroscopy

Fluorescence intensity quantification was performed on a spectrofluorometer (Cary Eclipse) following a literature-established protocol (Peng et al., 2021), a tyrosinase solution (200 U/mL) was prepared in phosphate buffer (pH = 6.8). Incremental volumes of **4c** solution at identical concentrations were added to the enzyme solution. With an excitation wavelength of 280 nm, an emission slit width of 5 nm, and the mixture was recorded in the 290–450 nm emission wavelength range.

### 2.9 ANS-binding fluorescence measurement

An equal volume of ANS solution (80 μM) was added to the tyrosinase solution (400 U/mL). After thorough mixing, the mixture was left to stand at room temperature for 30 min. Subsequently, a specific amount of the ANS-labeled tyrosinase solution was extracted, vigorously shaken, and **4c** was incrementally added dropwise to this mixture. The fluorescence spectrum of the mixture was scanned under conditions of an excitation wavelength of 390 nm, an emission slit width of 5 nm, and an emission wavelength range of 400–600 nm (Lü et al., 2010).

### 2.10 UV spectra measurement

Following the reported methods (Chen et al., 2022), a 3 mL assay system was established by adding a solution of **4c** (300 μM, 350 μL) and tyrosinase solution (200 U/mL, 400 μL) to 2250 μL of PBS at pH 6.8. Spectral measurements were carried out using a UV-1800 spectrophotometer with wavelength scanning from 200 to 400 nm. Three experimental controls were implemented: (1) PBS buffer as blank reference, (2) **4c** solution in PBS without enzyme, and (3) enzyme solution in PBS without **4c**. To ensure measurement reliability, all spectra were acquired in triplicate through independent scanning processes. Background corrections were systematically applied using corresponding control solutions for each experimental condition.

### 2.11 Molecular docking studies

Molecular docking was performed to study the binding mechanism of **4c** with tyrosinase at the molecular level using MOE software (v2024.06). The three-dimensional structure of **4c** was generated using Chem3D Ultra 8.0. The crystal structure of mTYR (PDB: 2Y9X) was downloaded from the PDB database (https://www.rcsb.org), and subjected to structural refinement by removing heteroatoms (water molecules, polyethylene glycol, and non-essential subunits), retaining only the catalytically active copper-binding domain as the receptor model (Pierce et al., 2012). Prior to molecular docking, both the protein and **4c** underwent energy minimization. Subsequently, the region closest to the copper ion active center was selected as the binding site for searching the optimal folded conformation of **4c**, and the optimal complex structure was selected for the interaction analysis between TYR and **4c**.

### 2.12 Stability evaluation

The purpose of **stability testing** is to provide evidence on how the quality of an active substance varies with time under the influence of a variety of environmental factors such as temperature, humidity, and light. Compound **4c** with the strongest activity was evaluated for its **stability**. **4c** was first dissolved in acetonitrile to prepare a 100 mM stock solution. This stock solution was then diluted to a 10 mM working concentration using different pH buffer solutions (4.0, 7.0 and 9.0). The samples were aliquoted into sealed vials. These vials were placed at different temperatures (25 °C, 40 °C and 60 °C) under both light-exposed and light-protected conditions for stability testing. The content change of the compound was monitored at scheduled time intervals (0 h, 24 h and 72 h) using an HPLC method.

### 2.13 Antibacterial properties

#### 2.13.1 Antibacterial activity

The antibacterial activity of **4c** against *Escherichia coli* (*E. coli*) and *Staphylococcus aureus* (*S. aureus*) was evaluated using a modified plate counting method based on literature reports (Ruan et al., 2024). In this process, the strains were first activated on LB agar medium at 37 °C for 18 h. Subsequently, bacterial suspensions (1 × 10^8^ CFU/mL) were prepared in fresh nutrient broth medium according to the 0.5 McFarland turbidity standard. These suspensions were then diluted to 1 × 10^5^ CFU/mL for antibacterial testing. Various concentrations of **4c** solutions were prepared and mixed with the 1 × 10^5^ CFU/mL bacterial suspensions, with equal volumes of physiological saline added to the control group. The mixtures were incubated at 37 °C for 12 h, serially diluted, plated (100 μL) onto plate count agar medium, and incubated for 24 h at 37 °C for colony counting. Each experiment was conducted in triplicate, and the antibacterial rate was calculated using the following formula (2):

(2) $$Bacterial\;Inhibition\;rate\;\left(\%\right)=\left(1-X_{S}\;/\;X_{0}\right)\times 100\%$$

where X_0_ represented the bacterial colony count of the control group post 24-h incubation, while X_S_ denoted the average bacterial colony count of the sample group.

#### 2.13.2 Antifungal activity

Fungal contamination is a major cause of food spoilage. To evaluate the antifungal potential of **4c**, its efficacy against the common phytopathogen *Botrytis cinerea* (*B. cinerea*) was investigated (Zhou et al., 2025). The assay was performed as follows: *B. cinerea* was first cultivated on Potato Dextrose Agar (PDA) to ensure robust growth. Subsequently, **4c** was prepared at various concentrations and incorporated into PDA to form a drug-containing medium. Fungal mycelia were then inoculated onto the center of both the treatment and control plates, with the latter containing an equal volume of physiological saline instead of the compound. All plates were incubated at 27 °C for 72 h. Following incubation, the colonial growth on each Petri dish was photographed, with particular attention to the mycelial morphology and expansion. The antifungal activity was quantified by measuring the radial growth of the fungus, and the inhibition rate was calculated according to formula (3):

(3) $$Fungal\;Inhibition\;rate\;\left(\%\right)=\left(1-D_{S}\;/\;D_{0}\right)\times 100\%$$

where D_0_ is the growth size of the control group, D_S_ is the growth size of the group containing the sample group.

### 2.14 Anti-browning study

Fresh and clean mushrooms of appropriate size were purchased from the local supermarket and their surfaces were evenly trimmed. Prepared test solutions (water, 100 μM kojic acid and 100 μM **4c**) were then evenly sprayed onto the mushroom surfaces, which were placed in sterile petri dishes and then stored at 4 °C in a refrigerator. CIE Lab values (L∗, a∗, and b∗) were measured using a colorimeter (NR60CP) to monitor the color changes of the fresh-cut mushrooms (Wang et al., 2023). The browning progression on mushroom surfaces was monitored *via* daily photographic documentation. The anti-browning efficacy was quantified using the total color difference (ΔE), calculated according to formula (4):

(4) $$\Delta E={\left[{\left(L_{t}^{*}-L_{initial}^{*}\right)}^{2}+{\left(a_{t}^{*}-a_{initial}^{*}\right)}^{2}+{\left(b_{t}^{*}-b_{initial}^{*}\right)}^{2}\right]}^{0.5}$$

## 3 Results and discussion

### 3.1 Chemistry

As depicted in Fig. 1, KA (**1**) was used as the starting material to synthesize compounds **4a‒4p**. Kojic acid was reacted with SOCl_2_ to obtain the key intermediate (**2**) using the method based on literature reports (Ashooriha et al., 2020). Title compounds **4a‒4p** were synthesized by the nucleophilic substitution reaction of intermediate **2**, secondary amine **3** and triethylamine in methanol overnight at room temperature. The structures of all hybrids **4a‒4p** were characterized by ^1^H-NMR, ^13^C-NMR, and HRMS.

### 3.2 Tyrosinase inhibitory activity

The tyrosinase inhibition was assessed using two substrates, L-tyrosine and L-DOPA. The IC_50_ values of kojic acid-tetrahydroisoquinoline hybrids (**4a‒4p**) and the positive control on tyrosinase inhibition were presented in Table 1. All hybrids exhibited superior anti-tyrosinase activity relative to kojic acid (IC_50_ = 43.63 ± 1.52 μM), amongst them, compound **4c** showed the most potent tyrosinase inhibitory effects (IC_50_ = 1.27 ± 0.08 μM). Compared with 5-(2-hydroxyphenyl)-1,3,4-oxadiazole-2-thiol **2f** (IC_50_ = 17.7 ± 0.21 μM) (Zhang et al., 2025), **4c** can form a more stable and better-matched chelation with copper ions to occupy the active site of tyrosinase, thereby more effectively blocking the substrate binding to enzyme. The structure–activity relationship (SAR) analysis showed: (i) either ring-opening (**Series II**) or aromatic ring removal (**Series III**) of tetrahydroisoquinoline skeleton led to a significant decrease of tyrosinase inhibitory activity (**4e** > **4k**, **4g** > **4m**; **4e** > **4o**, **4p**); (ii) the effect of substituted group at tetrahydroisoquinoline on tyrosinase inhibition was **4c** (6-OCH_3_) > **4a** (6-OH), **4c** (6-OCH_3_) > **4d** (6,7-diOCH_3_) > **4e** (-H), **4g** (7-Br) > **4h** (7-F), **4e** (1-H) > **4f** (1-CH_3_); (iii) the effect of substituted position on tyrosinase inhibition was **4a** (6-OH) > **4b** (7-OH), **4n** (6-F) > **4m** (7-F), **4i** (5-Br) > **4h** (7-Br). SAR analysis showed that tetrahydroisoquinoline scaffold may be key for the binding with the enzyme active site, when the scaffold damaged, the activity reduced.

**Table 1:** Inhibition of mushroom tyrosinase by hybrids **4a‒4p** and kojic acid.

Compd.	IC_50_ (μΜ)		Compd.	IC_50_ (μΜ)	
	L-Tyr	L-Dopa		L-Tyr	L-Dopa
**4a**	15.60 ± 0.57∗∗∗	17.99 ± 0.39∗∗∗	**4i**	3.14 ± 1.18∗∗∗	9.03 ± 0.50∗∗∗
**4b**	16.48 ± 0.26∗∗∗	23.49 ± 0.53∗∗∗	**4j**	10.53 ± 0.70∗∗∗	15.71 ± 0.76∗∗∗
**4c**	1.27 ± 0.08∗∗∗	3.30 ± 0.94∗∗∗	**4k**	42.04 ± 1.49	42.94 ± 1.92
**4d**	4.01 ± 0.57∗∗∗	10.61 ± 0.80∗∗∗	**4l**	35.15 ± 1.02	39.82 ± 0.57
**4e**	7.30 ± 0.71∗∗∗	20.50 ± 0.60∗∗∗	**4m**	25.31 ± 1.62∗∗	35.26 ± 1.53
**4f**	24.67 ± 1.06∗∗	35.42 ± 0.66	**4n**	16.41 ± 0.85∗∗∗	26.35 ± 1.68∗∗
**4g**	19.57 ± 0.45∗∗∗	28.34 ± 1.02∗∗	**4o**	18.67 ± 1.37∗∗∗	19.69 ± 0.27∗∗∗
**4h**	5.19 ± 0.75∗∗∗	15.66 ± 0.87∗∗∗	**4p**	20.95 ± 0.72∗∗∗	25.84 ± 0.65∗∗
Kojic acid	41.75 ± 0.69	43.63 ± 1.52			

∗∗∗*p* < 0.001 in comparison with kojic acid.

∗∗*p* < 0.01 in comparison with kojic acid.

### 3.3 In vitro and in vivo toxicity studies

*In vitro cytotoxicity assay*: The safety of the synthesized hybrids as anti-browning agents on B16F10 cells (murine melanoma cells) and HaCaT cells (human immortalized epidermal keratinocytes) was evaluated. As shown in Fig. S2A and S2B, all compounds hardly affected the cell viability (the cell survival rate >80 % at 100 μM), indicating negligible cytotoxic effects on both cell lines. Consequently, new compounds were proven to be safe and effective anti-browning candidates.

*In vivo acute toxicity evaluation in zebrafish embryos*: The zebrafish embryotoxicity model is often used to assess the toxicity of active compounds. The results showed that no significant toxic effects were observed in either the control group or the **4c**-treated groups with varying concentrations in zebrafish embryos (Fig. S3A and B). While partial mortality characterized by coagulated fertilized eggs and failed yolk sac-tail bud separation was observed in the kojic acid group. This verified that **4c** had superior safety profiles compared to kojic acid in the zebrafish embryo model. Therefore, *in vitro* and in vivo toxicity evaluation verified **4c**′s low toxicity.

### 3.4 Enzyme kinetic analysis

**Fig. 2:**
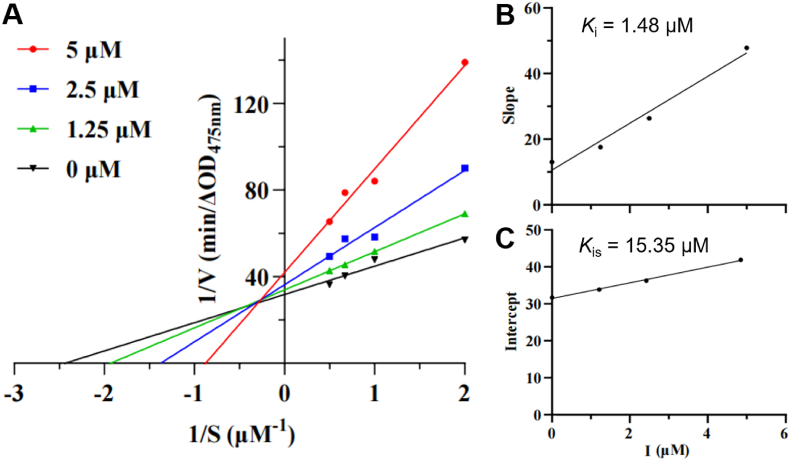
The Lineweaver-Burk plots for inhibition of tyrosinase on **4c**. (**A**) Lineweaver-Burk plots for **4c**; (**B**) The plot of slope versus the concentration of **4c** for determining the inhibition constants *K*_i_; (**C**) The plot of intercept versus the concentration of inhibitors for determining the inhibition constants *K*_is_.

Compound **4c**, the most potent tyrosinase inhibitor (Table 1), was subjected to mechanistic characterization *via* double-reciprocal Lineweaver-Burk plots. As shown in Fig. 2A, when the reciprocal of reaction rate 1/V was plotted against the reciprocal of substrate concentration 1/[S], the fitted lines at different inhibitor concentrations intersected in the second quadrant. Kinetic parameter analysis revealed that as the concentration of **4c** increased, the Michaelis-Menten constant (*K*_m_) of the system showed an increasing trend, while the maximum reaction rate (*V*_max_) gradually decreased. The kinetic characteristic conformed to the typical pattern of a mixed-type inhibitor, indicating that **4c** can bind to both free tyrosinase and the enzyme-substrate complex (You et al., 2022). However, the kojic acid-aromatic aldehyde hybrid **K-6j (**Peng et al., 2023) is a non-competitive tyrosinase inhibitor, which may be due to its *V*_max_ gradually decreasing with the increase of **K-6j**, while the *K*_m_ value remains unchanged. Further analysis of Fig. 2B and **C** showed that the binding inhibition constant (*K*_i_) of **4c** for free enzyme and the inhibition constant (*K*_is_) for the enzyme-substrate complex were 1.48 μM and 15.35 μM, respectively. The significantly lower *K*_i_ value compared to *K*_is_ (*K*_i_ *< K*_is_) confirmed stronger binding affinity of **4c** toward free tyrosinase than to enzyme-substrate complex (Yu and Fan, 2021).

### 3.5 Metal copper ion chelation

**Fig. 3:**
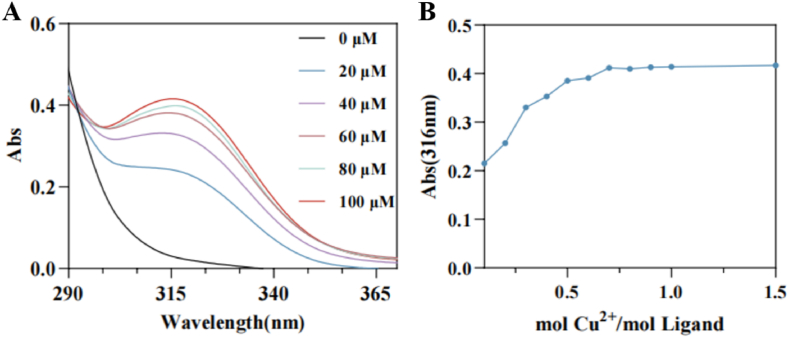
Copper-chelating ability of compound **4c**. (**A**) The UV spectra of inhibitor **4c** (100 μM) in the absence and presence of various concentrations of Cu^2+^ (0, 20, 40, 60, 80 and 100 μM) in methanol; (**B**) The ratio of Cu^2+^/Ligand.

The metal chelating capacity of **4c** toward copper ions was further investigated using UV–vis spectroscopy based on a previously reported method (Yap and Gan, 2021). As shown in Fig. 3A, with the addition of copper ions (0–100 μM), the fluorescence peak of **4c** gradually increased and shifted, which indicated that **4c** reduced the catalytic activity of TYR by chelating copper ions at the active site. Titration experiments were performed to determine the stoichiometric ratio of the **4c**-Cu^2+^ complex. Spectral changes at 316 nm were monitored when different concentrations of copper ions solution were added to **4c**. As depicted in Fig. 3B, the UV absorption intensity was significantly enhanced with increasing Cu^2+^/**4c** ratios. However, when the molar ratio reached 0.5, the absorption variation plateaued, suggesting that the stoichiometric ratio of **4c** to copper ion was 2: 1. Research has indicated that peptides capable of chelating copper ions exhibit a strong inhibitory effect on tyrosinase activity, thereby more effectively suppressing melanin production (Ju et al., 2022). It can be inferred that the hydroxy-pyranone moiety of **4c** possesses significant Cu^2+^ chelation ability, which contributes to its potent tyrosinase inhibitory effect.

### 3.6 Fluorescence quenching studies

**Fig. 4:**
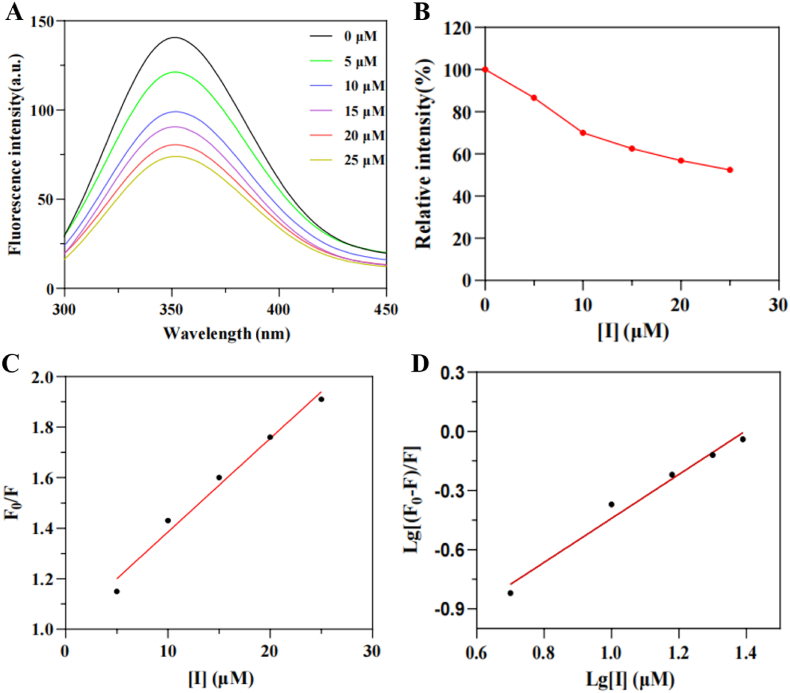
Fluorescence quenching spectra of **4c**. (**A**) Fluorescence emission spectra of tyrosinase in the presence of different concentrations of **4c**; (**B**) Relative intensities of tyrosinase at different concentrations of **4c**; (**C**) Stern-Volmer plot for the fluorescence quenching of tyrosinase; (**D**) Plot of log[(F_0_−F)/F] against log[I].

Fluorescence quenching studies were conducted to investigate the binding affinity between **4c** and tyrosinase, following established methodologies (J. Chen et al., 2020). As shown in Fig. 4A, in the absence of inhibitor **4c**, the fluorescence emission spectrum of tyrosinase was observed to peak at 354 nm. When **4c** concentration was increased to 25 μM, the fluorescence intensity decreased from 146.61 to 76.92. From Fig. 4B, it was determined that the relative intensity was reduced to 52 %. Generally, fluorescence quenching mechanisms are classified into static quenching and dynamic quenching. To elucidate the fluorescence quenching mechanism of **4c** on tyrosinase, the Stern-Volmer equation (formula 5) was applied to analyze the fluorescence quenching data (Min et al., 2023).

(5) $$F_{0}/F=1+K_{q}\tau_{0}\left[Q\right]=1+K_{\text{sv}}\left[Q\right]$$

Within the mathematical formalism, *F*_0_ and *F* denote the fluorescence emission values corresponding to pre- and post-addition of the quencher **4c**, respectively. *K*_q_ and *K*_sv_ are defined as the bimolecular quenching constant and Stern-Volmer quenching constant, respectively. τ_0_ denotes the fluorophore lifetime, and [*Q*] represents the concentration of **4c**. From the Stern-Volmer plot (Fig. 4C), *K*_q_ and *K*_sv_ were calculated as 4.3 × 10^12^ L mol^−1^ S^−1^ and 4.3 × 10^4^ L mol^−1^ S^−1^, respectively. Since the quenching rate constant *K*_q_ (4.3 × 10^12^ L mol^−1^ S^−1^) was found to exceed the maximum dynamic quenching constant for biomolecules (2 × 10^10^ L mol^−1^ S^−1^), it was preliminarily concluded that the quenching mechanism of **4c** on tyrosinase's intrinsic fluorescence was static quenching. For static quenching, the apparent binding constant (*K*_a_) and the number of binding sites (n) can be calculated using formula (6) (Zeng et al., 2020):

(6) $$\log\left[\left(F_{0}-F\right)/F\right]=\log\;K_{a}+n\;\log\left[Q\right]$$

In Fig. 4D, the values of *K*_a_ and n were calculated as 1.51 × 10^4^ L/mol and 1.11, respectively. The number of binding sites (n) for the compound **4c**-TYR complex was determined to be approximately equal to 1, indicating that there was only one binding site for **4c** with tyrosinase. Therefore, **4c** can directly bind to the active site of tyrosinase or specific nearby regions. This direct interaction interfered with the enzyme's catalytic function, a mechanism supported by observed fluorescence changes that signal alterations in the protein's conformation and the microenvironment of its active site. Notably, studies suggested that free inhibitors demonstrate a stronger binding affinity and more pronounced inhibitory effect on tyrosinase compared to pre-formed inhibitor-copper complexes. When an inhibitor pre-chelates copper ions, the functional groups essential for recognizing and binding Cu^2+^ within the active site are already occupied. This “pre-chelation” creates a steric hindrance, preventing the complex from effectively accessing the active site. As a result, the inhibition is diminished, correlating with a weaker fluorescence quenching signal (Wang et al., 2025).

### 3.7 ANS-binding fluorescence quenching studies

**Fig. 5:**
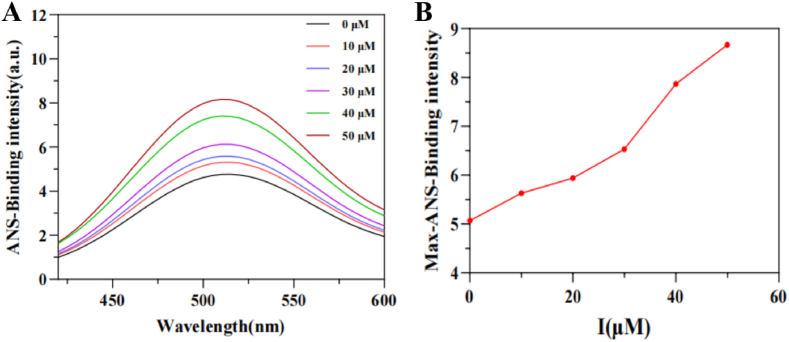
Fluorescence spectrum of tyrosinase binding to ANS under different concentrations of **4c**. (**A**) ANS-binding fluorescence spectra of tyrosinase with different concentration of **4c**; (**B**) The maximum ANS-tyrosinase fluorescence intensity with various concentration of **4c**.

ANS (1-anilinonaphthalene-8-sulfonate), a hydrophobic fluorescent probe, has been widely utilized in protein conformational studies. When ANS molecules are embedded into hydrophobic cavities and interact with nonpolar amino acid residues, significant enhancement of fluorescence emission is induced, making it an effective tool for investigating protein-ligand interactions (He et al., 2021). In the presence of **4c**, changes in the hydrophobicity of tyrosinase were recorded (Fig. 5). The maximum fluorescence intensity of ANS-TYR gradually increased as the concentration of **4c** increased from 0 μM to 50 μM. This phenomenon was attributed to the specific binding of **4c** to the hydrophobic structures on the tyrosinase surface, which led to conformational changes in the protein and enhanced the binding efficiency of the ANS probe to the hydrophobic microenvironment.

### 3.8 UV spectrum analysis

Ultraviolet spectrophotometry is widely known as a crucial technique for detecting protein structural changes. This method utilizes variations in UV absorption at 280 nm to monitor conformational and structural alterations in proteins (Shen et al., 2019). UV spectroscopic analysis was performed to comprehensively investigate the fluorescence quenching mechanism of **4c** on tyrosinase. UV absorption spectra with and without **4c** were measured separately. The results showed that the addition of **4c** resulted in a significant decrease in the intensity of the characteristic absorption peak of tyrosinase at 280 nm (Fig. S4). This phenomenon indicated that **4c** could cause significant changes in the secondary structure of TYR. Combining the consistency between the absorption spectroscopy and fluorescence spectroscopy results, the quenching mechanism of **4c** on TYR belonged to static quenching.

### 3.9 Molecular docking

**Fig. 6:**
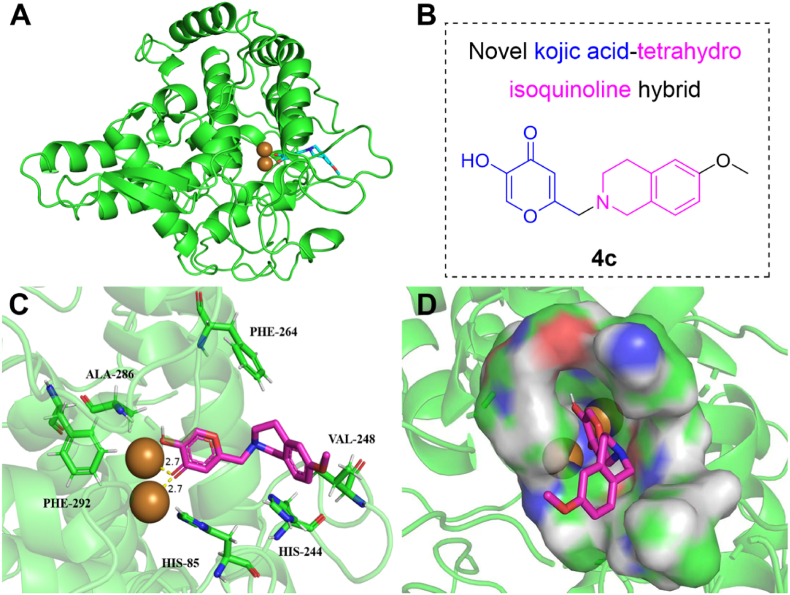
Molecular docking diagrams of **4c** interacted with tyrosinase (PDB ID: 2Y9X). (**A**) Overall structure of tyrosinase with **4c**; (**B**) chemical structure of **4c**; (**C**) binding pose of **4c** at binding site; (**D**) binding pose of **4c** in the surface of the binding pocket.

The binding mode of **4c** with tyrosinase was investigated through molecular docking. As shown in Fig. 6, Fig. 4c was found to bind tightly to the active site of tyrosinase (ΔG = −6.9803 kcal mol^−1^), and located within the hydrophobic core region of tyrosinase. Strong hydrophobic interactions were formed with residues such as Phe264, Phe292, Val248, and Ala286, which constitute a hydrophobic cavity. More importantly, the keto group of **4c** was observed to form two critical metal-chelating bonds with Cu^2+^ in the active site, with bond lengths of 2.7 Å and 2.7 Å, respectively (Fig. 6C and D). Collectively, these interactions were identified as the primary forces stabilizing the binding of **4c** to the active site, thereby inhibiting its catalytic activity.

As shown in Table S1, synthesized compounds had good binding affinity to tyrosinase (2Y9X) and their CDOCKER_INTERACTION_ENERGY had almost the same trend as tyrosinase inhibition (**4c** > **4d** > **4k** > **4o**), which further proved the correlation between tyrosinase inhibitory activity and binding energy. In the docking poses, differences in the tetrahydroisoquinoline moiety of **4d** and **4k**, as well as in the substituents of **4c**, affected the binding of their phenyl rings to the hydrophobic residue Val248. Compared with **4c**, the same trend was observed for kojic acid-1,3,4-oxadiazole hybrid **K-5f** (**4c**: ΔG = −6.9803 kcal mol^−1^, IC_50_ = 1.27 μM; **K-5f**: ΔG = −6.7073 kcal mol^−1^, IC_50_ = 5.32 μM) (Wang et al., 2023), while gallic acid-benzylidenehydrazine hybrid **G-5f** without kojic acid had higher binding affinity (ΔG = −7.2985 kcal mol^−1^), but it can't chelate Cu^2+^ ions to exhibit lower activity (IC_50_ = 3.30 μM) (Shen et al., 2019). Therefore, the metal-chelating bonds between keto group of **4c** with Cu^2+^ was key for tyrosinase inhibitory activity.

### 3.10 Stability evaluation

As shown in Table S2, the results of **stability testing** indicated that **4c** had excellent stability. After 72 h, the retention rates were maintained >95 % across all pH conditions (4.0, 7.0, and 9.0) and temperature conditions (25, 40 and 60 °C). Particularly under stringent conditions, including elevated temperature (60 °C) and alkaline environment (pH 9.0), **4c** still maintained high retention rates. A same trend was observed in the light exposure experiments. These data provided a solid foundation for confirming the remarkable stability of **4c**, thereby supporting its potential practical applications in food processing and storage.

### 3.11 Antibacterial activity

#### 3.11.1 Antimicrobial activity

**Fig. 7:**
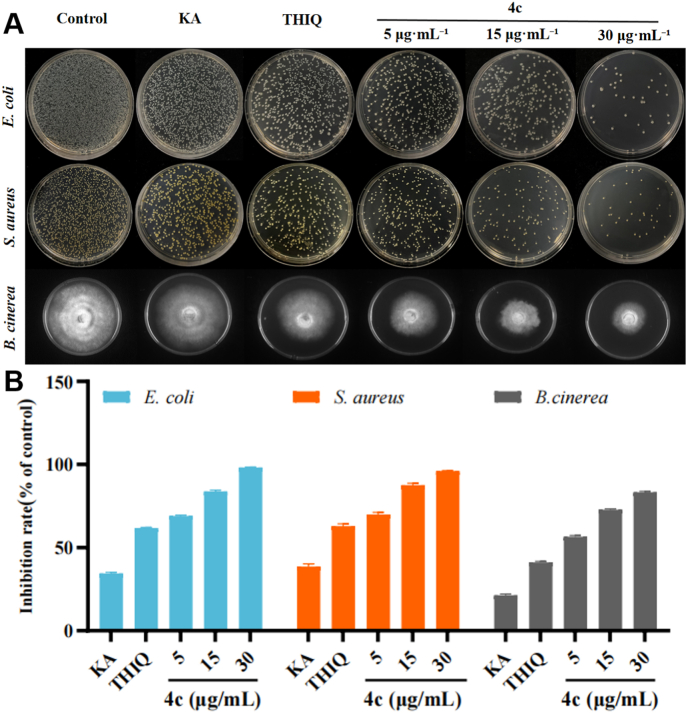
(**A**) Inhibitory properties of KA, THIQ, and different concentrations of **4c** against bacteria (*S. aureus and E. coli*) and fungi (*B. cinerea*); (**B**) Inhibition rates against bacteria (*S. aureus and E. coli*) and fungi (*B. cinerea*).

The antimicrobial experimental results demonstrated that **4c** exhibited significant concentration-dependent inhibitory effects on *E. coli* and *S. aureus*. As shown in Fig. 7A, no significant antibacterial effect was observed in the control group, while both the KA and THIQ groups displayed low antibacterial rates. In contrast, the antibacterial effect of **4c** was gradually enhanced with increasing concentrations (5, 15, and 30 μg/mL). Fig. 7B revealed that nearly 100 % inhibition rates against *E. coli* and *S. aureus* were achieved by **4c** at high concentrations, indicating stronger antibacterial capability compared to KA and THIQ. In a word, compared with the KA and THIQ, **4c** exhibited a stronger antibacterial rate at the same concentration, highlighting its unique structural advantages or stronger target affinity. The low activity of KA and THIQ may be due to their structures being unable to effectively act on key life processes of bacteria.

#### 3.11.2 Antifungal activity

The experimental results indicated that compound **4c** had exhibited considerable antifungal efficacy. As shown in Fig. 7A, the colony diameter of *B.cinerea* had gradually shrunk with increasing concentrations of **4c** (5, 15, and 30 μg/mL). Fig. 7B demonstrated that the inhibition rate of **4c** against *B.cinerea* reached nearly 90 % at high concentrations, and its antifungal activity had been stronger than that of KA and THIQ.

### 3.12 Anti-browning experiment

**Fig. 8:**
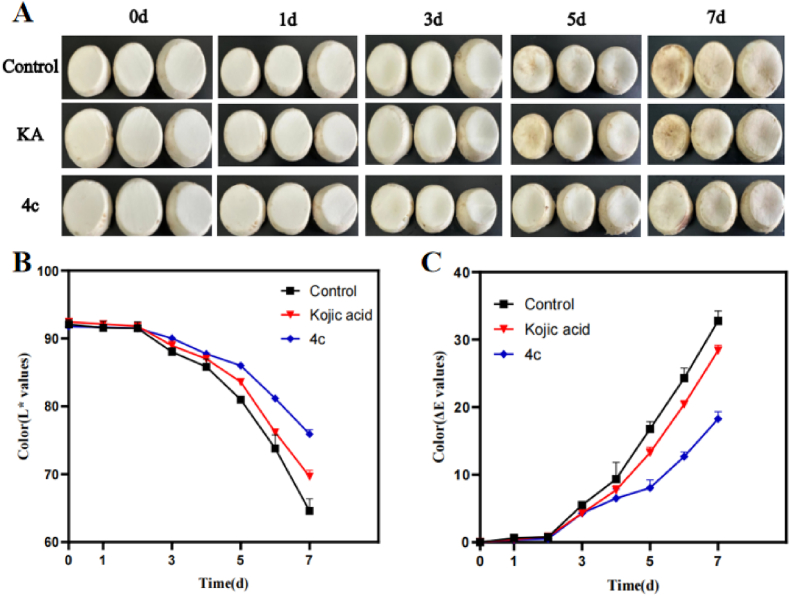
Anti-browning effect of compound **4c** on fresh-cut mushrooms. (**A**) Surface appearance of fresh-cut mushrooms after KA and **4c** treatment; (**B**) L∗ value changes; (**C**) ΔE value changes.

The browning and discoloration of fresh-cut fruits are primarily driven by enzymatic and non-enzymatic reactions, the extent of which can be quantified using chromatic parameters (*L*∗, *a*∗, *b*∗) and the total color difference (ΔE) (Zhu et al., 2022). In this study, the *L*∗ (lightness) and ΔE were used as key indicators to assess the anti-browning efficacy of **4c** on fresh-cut mushrooms. As shown in Fig. 8B and C, during the 7-day storage period, the **4c**-treated group maintained the highest *L*∗ value and the lowest ΔE value, followed by the kojic acid (KA) group and then the control. By day 7, the anti-browning effect of **4c** was significantly superior to that of the control (*p* < 0.001) and the KA group (*p* < 0.01). These colorimetric results were corroborated by physical texture observations (Fig. 8A). On day 7, mushrooms treated with **4c** retained a firm and elastic texture, quickly recovering their shape after compression without shrinkage. In contrast, both the control and KA groups became soft and tough, exhibiting indentations and slow recovery after pressing. Polyphenol oxidase (PPO) is a key factor that triggers enzymatic browning in fruits and vegetables. This is supported by studies that gallotannin derivatives from *Galla rhois* can bind to and inactivate PPO (Lee et al., 2022). Analogously, **4c** may exhibit a strong affinity for mushroom PPO, potentially greater than that of KA, effectively suppressing the browning reaction and preserving product quality.

## 4 Conclusion

This study successfully designed and synthesized sixteen novel kojic acid-tetrahydroisoquinoline hybrids (**4a–4p**) as multifunctional tyrosinase inhibitors for food preservation, and comprehensively elucidated their mechanisms of action. Among them, compound **4c** demonstrated exceptional potency, exhibiting superior inhibitory activity against both monophenolase (IC_50_ = 1.27 ± 0.08 μM) and diphenolase (IC_50_ = 3.30 ± 0.94 μM) compared to the control kojic acid (IC_50_ = 41.75 ± 0.69 μM and 43.63 ± 1.52 μM, respectively). SAR analysis revealed that the intact tetrahydroisoquinoline skeleton and the position of substituents critically influence activity, with the 6-methoxy group enhancing binding affinity. Mechanistic studies indicated that **4c** acts as a mixed-type inhibitor, capable of binding to both the free tyrosinase and the enzyme-substrate complex. Copper ion chelation assays and molecular docking simulations suggested that **4c** interacts tightly with tyrosinase, likely *via* chelation. Furthermore, fluorescence quenching studies and UV–visible spectroscopy confirmed that **4c** binds directly to the enzyme's active site or a specific adjacent region, inducing a conformational change in TYR.

Additional stability tests showed that **4c** maintained a high retention rate under various conditions. Cytotoxicity assays and zebrafish acute toxicity tests both indicated that **4c** possesses a high safety profile. **4c** also exhibited broad-spectrum antimicrobial activity against both bacteria and fungi. In fresh mushroom slice models, **4c** significantly delayed browning. These findings highlighted the potential of **4c** as a multifunctional anti-browning agent. It combines potent tyrosinase inhibition, antimicrobial activity, and a favorable safety profile, addressing key challenges in food preservation.

## Author contributions

Min Lv: Writing-original draft, Methodology, Investigation, Data curation. Longkang Cao: Investigation, Formal analysis, Conceptualization. Yulu Ding: Software, Methodology, Formal analysis. Shan Gao: Methodology, Formal analysis, Data curation. Yinxin Wu: Investigation, Data curation. Rong Li: Supervision, Methodology, Data curation. Wenjian Tang: Writing-review & editing, Supervision, Methodology, Funding acquisition. Lili Zhu: Writing-review & editing, Methodology, Funding acquisition, Data curation.

## Declaration of competing interest

The authors declare that they have no known competing financial interests or personal relationships that could have appeared to influence the work reported in this paper.

Wenjian Tang and other authors declare that they have no conflict of interests.

## Data Availability

No data was used for the research described in the article.
