# Supplementary material for: Multifunctional kojic acid-tetrahydroisoquinoline hybrids: Synthesis, tyrosinase inhibition, and applications in the anti-browning of fresh-cut mushrooms
Source: Curr Res Food Sci. 2025 Nov 16;11:101248. doi: 10.1016/j.crfs.2025.101248 (PMC12681733; doi:10.1016/j.crfs.2025.101248)
Supplement: Multimedia component 1 [file mmc1.pdf]

Multifunctional kojic acid-tetrahydroisoquinoline hybrids: Synthesis, tyrosinase inhibition, and applications in the anti-browning of fresh-cut mushrooms

Min Lv [a], Longkang Cao [a], Yulu Ding [a], Shan Gao [a], Yinxin Wu [a], Rong Li [a,*], Wenjian Tang [a,*], Lili Zhu [a,*]

[a] *School of Pharmacy, Inflammation and Immune Mediated Diseases Laboratory of Anhui Province, Anhui Medical University, Hefei 230032, China*

Content

Fig. 1. Rationale design of title compounds of this work.................................**S2**

Fig. S2. Cell viability of title compounds...**S3**

Fig. S3. Acute toxicity assay in zebrafish embryos...................................**S4**

Fig. S4. UV absorption spectra of **4c**...**S4**

Table S1. -CDOCKER_INTERACTION_ENERGY of title compounds..............**S5**

Table S2. Stability testing...**S6**

The characterized data of compounds **4a–4p**....................................**S7–S11**

Copies of NMR, HPLC and HRMS spectra of compounds **4a–4p**.............**S12–S44**

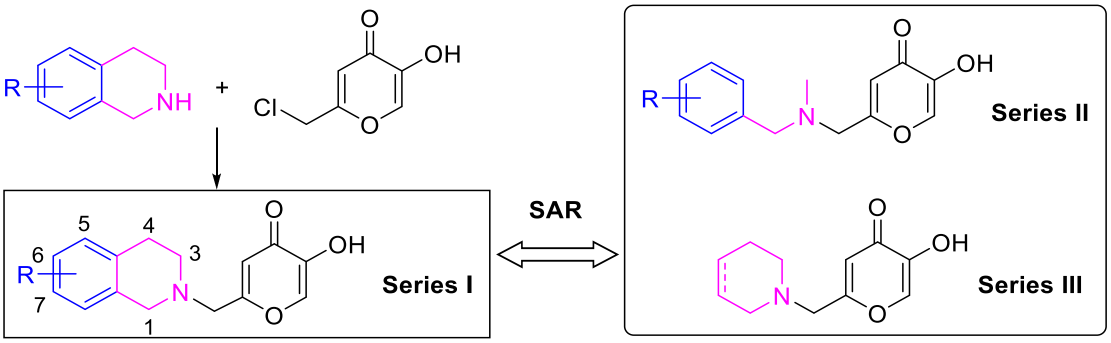

Fig. 1. Rationale design of title compounds of this work.

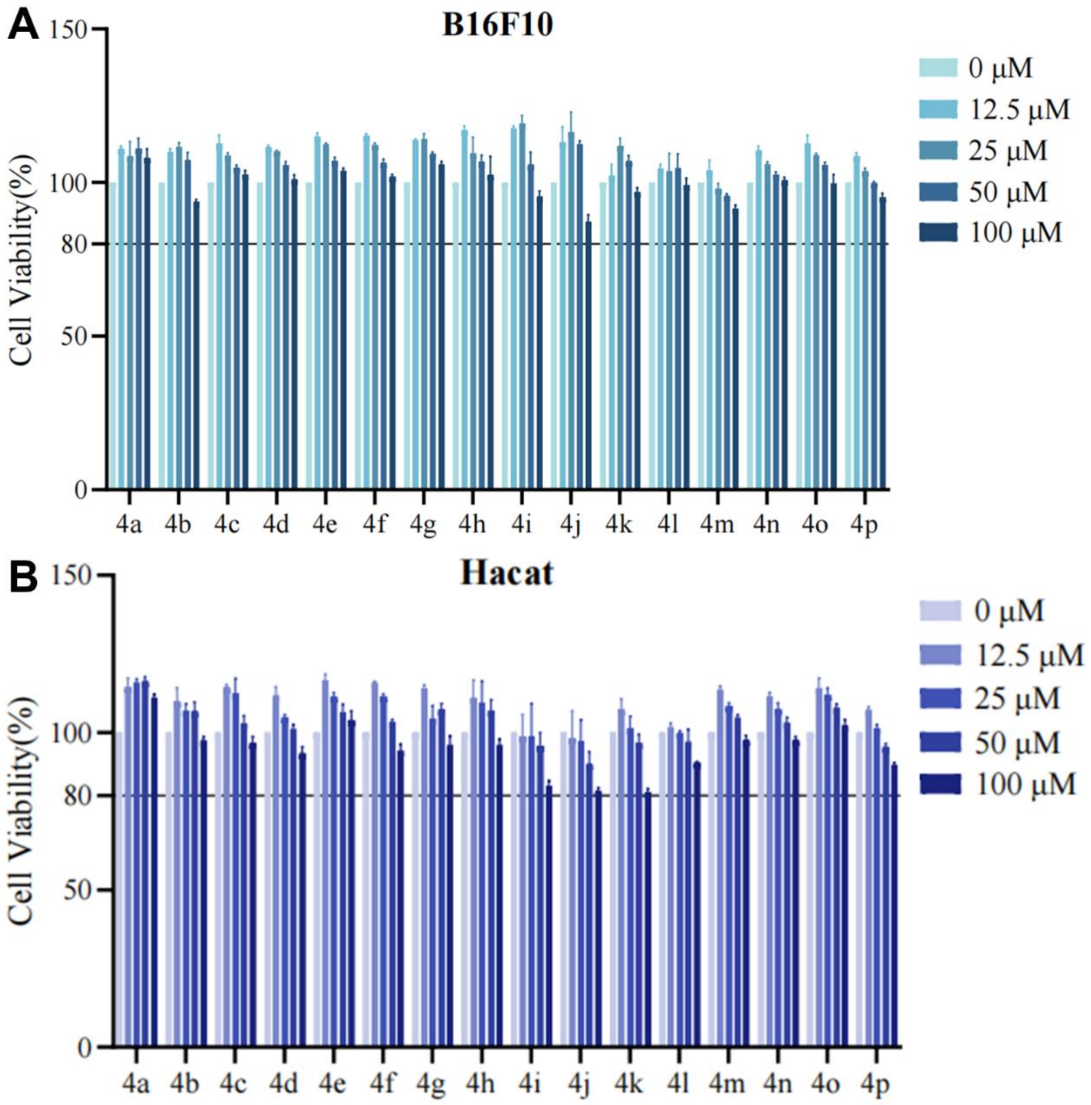

Fig. S2. Cell viability of title compounds in B16F10 cells (**A**) and HaCaT cells (**B**). HaCaT and B16F10 cells were treated with compounds **4a–4p** (0, 12.5, 25, 50, and 100 μM) for 24 h, respectively.

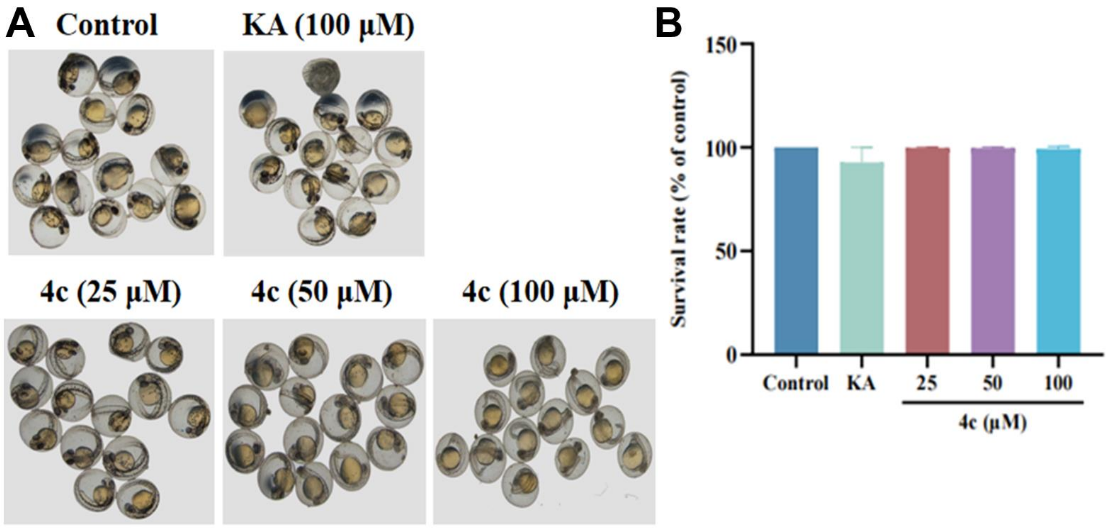

Fig. S3. Acute toxicity assay in zebrafish embryos. (**A**) Embryonic morphology was observed under a stereomicroscope; (**B**) Survival rates of embryos treated with kojic acid and compound **4c** were analyzed.

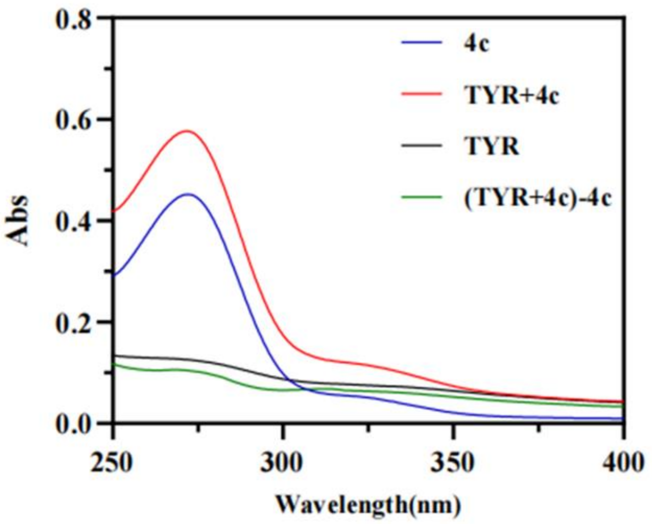

Fig. S4. UV absorption spectra of **4c** before and after adding tyrosinase.

Table S1. -CDOCKER_INTERACTION_ENERGY of title compounds and tyrosinase under the GBVI/WSA dG (kcal/mol) scoring function.

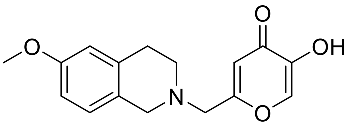

4c

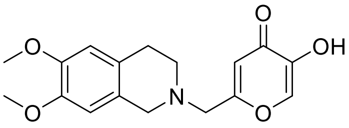

4d

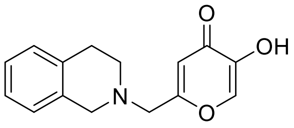

4k

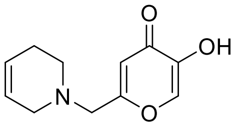

4o

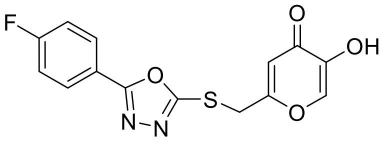

K-5f

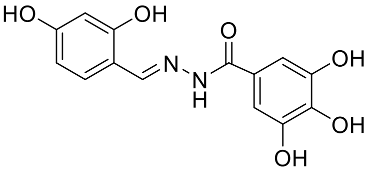

G-5f

Compd.	-CDOCKER_INTERACTION_ENERGY ΔG (kcal/mol)
**4c**	6.9803
**4d**	6.7440
**4k**	6.4584
**4o**	6.1014
**K-5f**	6.7073
**G-5f**	7.2985

Table S2. Stability testing of compound **4c** under the influence of temperature, humidity, and light

**Temperature**	**pH**	**0 h**	**24 h**	**72 h**
25℃	4	97.01%	98.10%	98.32%
	7	98.63%	97.48%	98.03%
	9	97.43%	97.48%	97.59%
40℃	4	97.02%	97.50%	97.41%
	7	97.56%	96.04%	97.75%
	9	96.51%	95.00%	96.12%
60℃	4	98.22%	96.90%	97.85%
	7	96.64%	96.84%	96.62%
	9	96.61%	96.04%	96.26%
**Light**	4	98.22%	96.90%	97.85%
	7	96.64%	96.84%	96.62%
	9	96.61%	96.04%	96.26%

The characterized data of compounds **4a–4p** including ^{1}H-NMR, ^{13}C-NMR, HPLC and HRMS

5-hydroxy-2-((6-hydroxy-3,4-dihydroisoquinolin-2(1H)-yl)methyl)-4H-pyran-4-one (4a). Off-white powder; yield: 90%, mp: 144.7-146.2 °C, HPLC purity: 98.9%; ^{1}H NMR (600 MHz, $CDCl_3$) δ 8.75 (s, 1H, OH), 8.56 (s, 1H, OH), 7.82 (s, 1H), 6.80 (d, *J* = 8.0 Hz, 1H), 6.66 – 6.50 (m, 2H), 6.45 (s, 1H), 3.59 (s, 2H, NCH_2), 3.55 (s, 2H, NCH_2), 2.89 – 2.68 (m, 4H, NCH_2); ^{13}C NMR (151 MHz, $CDCl_3$) δ 174.0 (C=O), 164.7, 155.2, 145.7, 138.7, 134.2, 126.8, 124.1, 114.4, 113.0, 112.2, 58.6 (NCH_2), 54.8 (NCH_2), 50.3 (NCH_2), 28.6 (CH_2); TOF-HRMS (ESI) *m/z*: $[M + H]^+$, calcd for $C_{15}H_{16}NO_4$: 274.1074; found: 274.1073.

5-hydroxy-2-((7-hydroxy-3,4-dihydroisoquinolin-2(1H)-yl)methyl)-4H-pyran-4-one (4b). Off-ginger powder; yield: 83%, mp: 142.4-143.2 °C, HPLC purity: 96.7%; ^{1}H NMR (600 MHz, $CDCl_3$) δ 7.85 (s, 1H), 6.91 (d, *J* = 8.4 Hz, 1H), 6.70 (dd, *J* = 8.4, 2.3 Hz, 1H), 6.66 – 6.61 (m, 1H), 6.57 (s, 1H), 3.65 (s, 2H, NCH_2), 3.57 (s, 2H, NCH_2), 2.90 (d, *J* = 5.6 Hz, 2H), 2.80 (d, *J* = 5.7 Hz, 2H, NCH_2); ^{13}C NMR (151 MHz, $CDCl_3$) δ 174.4 (C=O), 166.2, 158.3, 145.8, 138.1, 134.9, 127.6, 126.1, 113.4, 112.4, 111.9, 59.4 (NCH_2), 55.4 (NCH_2), 50.9 (NCH_2), 21.2 (CH_2); TOF-HRMS (ESI) *m/z*: $[M + H]^+$, calcd for $C_{15}H_{16}NO_4$: 274.1074; found: 274.1074.

5-hydroxy-2-((6-methoxy-3,4-dihydroisoquinolin-2(1H)-yl)methyl)-4H-pyran-4-one (4c). Off-white powder; yield: 78%, mp: 124.4-126.3°C, HPLC purity: 98.6%; ^{1}H NMR (600 MHz, DMSO-d_6) δ 9.14 (s, 1H, OH), 8.06 (s, 1H), 6.94 (d, *J* = 8.2 Hz, 1H), 6.75 – 6.61 (m, 2H), 6.40 (s, 1H), 3.69 (s, 3H, OCH_3), 3.54 (m, 4H, $2NCH_2$), 2.79 (t, *J* = 5.7 Hz, 2H), 2.71 (t, *J* = 5.5 Hz, 2H, NCH_2); ^{13}C NMR (151 MHz, DMSO-d_6) δ 173.8 (C=O), 164.9, 157.6, 145.9, 139.8, 135.0, 127.4, 126.4, 113.0, 112.7, 112.1, 58.2 (NCH_2), 54.9 (OCH_3), 54.5 (NCH_2), 50.2 (NCH_2), 28.9 (CH_2); TOF-HRMS (ESI) *m/z*: $[M + H]^+$, calcd for $C_{16}H_{18}NO_4$: 288.1231; found: 288.1236.

2-((6,7-dimethoxy-3,4-dihydroisoquinolin-2(1H)-yl)methyl)-5-hydroxy-4H-pyran-4-one (4d). Off-ginger powder; yield: 82%, mp: 164.4-166.6°C, HPLC purity: 97.0%; ^{1}H NMR (600 MHz, $CDCl_3$) δ 7.86 (s, 1H), 6.60 (s, 1H), 6.58 (s, 1H), 6.49 (s, 1H), 3.85 (s, 3H, OCH_3), 3.82 (s, 3H, OCH_3), 3.64 (s, 2H, NCH_2), 3.58 (s, 2H, NCH_2), 2.84 (d, J = 5.1 Hz, 2H), 2.82 (d, J = 5.0 Hz, 2H, NCH_2); ^{13}C NMR (151 MHz, $CDCl_3$) δ 174.3 (C=O), 166.4, 147.9, 147.5, 145.7, 137.9, 125.7, 125.6, 111.9, 111.5, 109.4, 59.4 (NCH_2), 56.1 (OCH_3), 56.1 (OCH_3), 55.5 (NCH_2), 51.1 (NCH_2), 28.5 (CH_2); TOF-HRMS (ESI) *m/z*: $[M + H]^+$, calcd for $C_{17}H_{20}NO_5$: 318.1336; found: 318.1340.

2-((3,4-dihydroisoquinolin-2(1H)-yl)methyl)-5-hydroxy-4H-pyran-4-one (4e). Off-white powder; yield: 78%, mp: 174.2-175.1°C, HPLC purity: 98.5%; ^{1}H NMR (600 MHz, $CDCl_3$) δ 7.86 (s, 1H), 7.15 (s, 1H), 7.12 (s, 1H), 7.11 (s, 1H), 6.99 (d, J = 7.2 Hz, 1H), 6.58 (s, 1H), 3.72 (s, 2H, NCH_2), 3.59 (s, 2H, NCH_2), 2.93 (d, J = 5.8 Hz, 2H), 2.84 (d, J = 5.8 Hz, 2H, NCH_2); ^{13}C NMR (151 MHz, $CDCl_3$) δ 174.4 (C=O), 166.2, 145.9, 138.3, 133.8, 133.8, 128.9, 126.6, 126.6, 125.9, 112.0, 59.4 (NCH_2), 55.9 (NCH_2), 50.9 (NCH_2), 28.9 (CH_2); TOF-HRMS (ESI) *m/z*: $[M + H]^+$, calcd for $C_{15}H_{16}NO_3$: 258.1125; found: 258.1122.

5-hydroxy-2-((1-methyl-3,4-dihydroisoquinolin-2(1H)-yl)methyl)-4H-pyran-4-one (4f). Off-brick red powder; yield: 90%, mp: 134.2-135.6°C, HPLC purity: 96.9%; ^{1}H NMR (600 MHz, $CDCl_3$) δ 7.81 (s, 1H), 7.17 – 7.00 (m, 4H), 6.59 (s, 1H), 3.85 (d, J = 6.7 Hz, 1H), 3.61 (s, 2H, NCH_2), 3.12 (m, 1H), 2.89 (m, 1H), 2.75 (m, 2H, NCH_2), 1.36 (d, J = 6.7 Hz, 3H, CH_3); ^{13}C NMR (151 MHz, $CDCl_3$) δ 174.6 (C=O), 167.5, 145.8, 139.1, 138.3, 133.5, 128.9, 127.4, 126.2, 125.9, 111.5, 56.8 (NCH_2), 55.6 (NCH_2), 44.8 (NCH_2), 27.1 (CH_2), 20.3 (CH_3); TOF-HRMS (ESI) *m/z*: $[M + H]^+$, calcd for $C_{16}H_{18}NO_3$: 272.1281; found: 272.1280.

2-((7-fluoro-3,4-dihydroisoquinolin-2(1H)-yl)methyl)-5-hydroxy-4H-pyran-4-one (4g). Off-white powder; yield: 88%, mp: 137.2-139.5°C, HPLC purity: 98.3%; ^{1}H NMR

(600 MHz, $CDCl_3$) δ 7.86 (s, 1H), 7.06 (dd, J = 8.3, 5.8 Hz, 1H), 6.85 (td, J = 8.5, 2.5 Hz, 1H), 6.70 (dd, J = 9.3, 2.3 Hz, 2H), 6.57 (s, 1H), 3.69 (s, 2H, NCH_2), 3.58 (s, 2H, NCH_2), 2.87 (d, J = 5.6 Hz, 2H), 2.82 (d, J = 5.6 Hz, 2H, NCH_2); ^{13}C NMR (151 MHz, $CDCl_3$) δ 174.3 (C=O), 166.0, 161.1 (d, J = 240 Hz, C-F), 145.8, 138.1, 135.7 (d, J = 7.5 Hz), 130.3 (d, J = 7.5 Hz), 129.3 (d, J = 3 Hz), 113.8 (d, J = 21 Hz), 112.9 (d, J = 21 Hz), 111.95, 59.2 (NCH_2), 55.7 (d, J = 1. 5 Hz, NCH_2) 50.9 (NCH_2), 28.29 (CH_2); TOF-HRMS (ESI) *m/z*: $[M + H]^+$, calcd for $C_{15}H_{15}FNO_3$: 276.1031; found: 276.1031.

2-((7-bromo-3,4-dihydroisoquinolin-2(1H)-yl)methyl)-5-hydroxy-4H-pyran-4-one (4h). Off-white powder; yield: 95%, mp: 127.2-129.3°C, HPLC purity: 96.4%; ^1H NMR (600 MHz, $CDCl_3$) δ 7.97 (s, 1H), 7.37 (d, J = 6.2 Hz, 1H), 7.09 (d, J = 8.1 Hz, 1H), 6.78 (s, 1H), 6.68 (s, 1H), 3.79 (s, 2H, NCH_2), 3.69 (s, 2H, NCH_2), 2.97 (d, J = 5.4 Hz, 2H), 2.93 (d, J = 5.5 Hz, 2H, NCH_2); ^{13}C NMR (151 MHz, $CDCl_3$) δ 174.3 (C=O), 165.9, 145.8, 138.1, 136.1, 132.8, 130.5, 129.7, 129.5, 119.5, 111.9, 59.1 (NCH_2), 55.4 (NCH_2), 50.6 (NCH_2), 28.5 (CH_2); TOF-HRMS (ESI) *m/z*: $[M + H]^+$, calcd for $C_{15}H_{15}BrNO_3$: 336.023; found: 336.0229.

2-((5-bromo-3,4-dihydroisoquinolin-2(1H)-yl)methyl)-5-hydroxy-4H-pyran-4-one (4i). Off-light yellow powder; yield: 94%, mp: 113.6-114.9°C, HPLC purity: 97.7%; ^1H NMR (600 MHz, $CDCl_3$) δ 7.86 (s, 1H), 7.41 (d, J = 7.8 Hz, 1H), 7.01 (t, J = 7.7 Hz, 1H), 6.95 (d, J = 7.6 Hz, 1H), 6.56 (s, 1H), 3.69 (s, 2H, NCH_2), 3.58 (s, 2H, NCH_2), 2.88 (d, J = 4.8 Hz, 2H), 2.86 (d, J = 4.7 Hz, 2H, NCH_2); ^{13}C NMR (151 MHz, $CDCl_3$) δ 174.3 (C=O), 165.9, 145.8, 138.2, 136.3, 133.7, 130.7, 127.3, 125.8, 125.4, 112.0, 59.0 (NCH_2), 55.9 (NCH_2), 51.09 (NCH_2), 30.1 (CH_2); TOF-HRMS (ESI) *m/z*: $[M + H]^+$, calcd for $C_{15}H_{15}BrNO_3$: 336.023; found: 336.0231.

5-hydroxy-2-((7-(trifluoromethyl)-3,4-dihydroisoquinolin-2(1H)-yl)methyl)-4H-pyran-4-one (4j). Off-white powder; yield: 78%, mp: 145.6-147.8°C, HPLC purity: 95.4%; ^1H NMR (600 MHz, $CDCl_3$) δ 7.84 (s, 1H), 7.37 (d, J = 8.0 Hz, 1H), 7.24 (s,

1H), 7.20 (d, *J* = 8.0 Hz, 1H), 6.55 (s, 1H), 3.74 (s, 2H, NCH_2), 3.59 (s, 2H, NCH_2), 2.95 (t, *J* = 5.5 Hz, 2H), 2.83 (t, *J* = 5.8 Hz, 2H, NCH_2); ^{13}C NMR (151 MHz, $CDCl_3$) δ 174.3 (C=O), 165.6, 145.9, 138.4, 137.9, 134.6, 129.2, 128.2, 124.2 (q, J = 271.9 Hz, CF3), 123.5, 123.2, 112.1, 59.0 (NCH_2), 55.5 (NCH_2), 50.4 (NCH_2), 28.9 (CH_2); TOF-HRMS (ESI) *m/z*: $[M + H]^+$, calcd for $C_{16}H_{15}F_3NO_3$: 326.0999; found: 326.0995.

2-((benzyl(methyl)amino)methyl)-5-hydroxy-4H-pyran-4-one (4k). Off-white powder; yield: 92%, mp: 154.6-156.8°C, HPLC purity: 97.6%; ^1H NMR (600 MHz, $CDCl_3$) δ 7.83 (s, 1H), 7.37 – 7.27 (d, *J* = 4.4 Hz, 4H), 7.26 (d, *J* = 4.8 Hz, 1H), 6.58 (s, 1H), 3.60 (s, 2H, NCH_2), 3.41 (s, 2H, NCH_2), 2.30 (s, 3H, CH_3).; ^{13}C NMR (151 MHz, $CDCl_3$) δ 174.4 (C=O), 167.2, 145.8, 138.2, 138.0, 128.9 (2C), 128.6 (2C), 127.6, 111.7, 61.9 (NCH_2), 58.1 (NCH_2), 42.7 (NCH_3); TOF-HRMS (ESI) *m/z*: $[M + H]^+$, calcd for $C_{14}H_{15}NO_3$: 246.1125; found: 246.1125.

5-hydroxy-2-((methyl(4-methylbenzyl)amino)methyl)-4H-pyran-4-one (4l). Off-white powder; yield: 76%, mp: 167.6-168.8°C, HPLC purity: 97%; ^1H NMR (600 MHz, $CDCl_3$) δ 7.82 (s, 1H), 7.20 (d, *J* = 7.8 Hz, 2H), 7.13 (d, *J* = 7.8 Hz, 2H), 6.56 (s, 1H), 3.56 (s, 2H, NCH_2), 3.39 (s, 2H, NCH_2), 2.33 (s, 3H, NCH_3), 2.29 (s, 3H, CH_3); ^{13}C NMR (151 MHz, $CDCl_3$) δ 174.4 (C=O), 167.3, 145.8, 138.1, 137.2, 134.9, 129.2 (2C), 128.9 (2C), 111.6, 61.7 (NCH_2), 57.9 (NCH_2), 42.7 (CH_3), 21.2 (CH_3); TOF-HRMS (ESI) *m/z*: $[M + H]^+$, calcd for $C_{15}H_{18}NO_3$: 260.1281; found: 260.1286.

2-(((3-fluorobenzyl)(methyl)amino)methyl)-5-hydroxy-4H-pyran-4-one (4m). Off-earthy yellow powder; yield: 79%, mp: 156.6-158.9°C, HPLC purity: 96.8%; ^1H NMR (400 MHz, DMSO-d_6) δ 8.05 (s, 1H), 7.50 – 7.30 (m, 1H), 7.21 – 7.12 (m, 2H), 7.07 (d, *J* = 2.5 Hz, 1H), 6.39 (s, 1H), 3.58 (s, 2H, NCH_2), 3.44 (s, 2H, NCH_2), 2.16 (s, 3H, NCH_3); ^{13}C NMR (101 MHz, DMSO-d_6) δ 173.9 (C=O), 165.1, 162.3 (d, *J* = 375 Hz, C-F), 146.1, 141.7 (d, *J* = 15 Hz), 139.7, 130.2 (d, *J* = 15 Hz), 124.6 (d, *J* = 3 Hz), 115.1

(d, J = 31.5 Hz), 113.9 (d, J = 31.5 Hz), 112.3, 60.1 (NCH_2), 57.5 (NCH_2), 41.7 (CH_3). TOF-HRMS (ESI) *m/z*: $[M + H]^+$, calcd for $C_{14}H_{15}FNO_3$: 264.1031; found: 264.1033.

2-(((4-fluorobenzyl)(methyl)amino)methyl)-5-hydroxy-4H-pyran-4-one (4n). Off-light orange powder; yield: 80%, mp: 165.6-168.5°C, HPLC purity: 97.8%; ^{1}H NMR (600 MHz, $CDCl_3$) δ 7.83 (s, 1H), 7.28 (t, J = 6.2 Hz, 2H), 7.00 (t, J = 8.1 Hz, 2H), 6.80 (s, 1H, OH), 6.56 (s, 1H), 3.56 (s, 2H, NCH_2), 3.40 (s, 2H, NCH_2), 2.28 (s, 3H, NCH_3); ^{13}C NMR (151 MHz, $CDCl_3$) δ 174.4 (C=O), 167.1, 162.3 (d, J = 240 Hz, C-F), 145.8, 138.1, 133.8 (d, J = 3 Hz), 130.4 (d, J = 9 Hz, 2C), 115.4 (d, J = 21 Hz, 2C), 111.6, 61.2 (NCH_2), 58.0 (NCH_2), 42.5 (CH_3); TOF-HRMS (ESI) *m/z*: $[M + H]^+$, calcd for $C_{14}H_{15}FNO_3$: 264.1031; found: 264.1027.

2-((3,6-dihydropyridin-1(2H)-yl)methyl)-5-hydroxy-4H-pyran-4-one (4o). Off-yellow crystal; yield: 83%, mp: 143.6-145.7°C, HPLC purity: 96.4%; ^{1}H NMR (600 MHz, $CDCl_3$) δ 7.81 (s, 1H), 6.48 (s, 1H), 5.73 (d, J = 10.0 Hz, 1H), 5.61 (d, J = 10.0 Hz, 1H), 3.46 (s, 2H, NCH_2), 3.02 (s, 2H, NCH_2), 2.60 (d, J = 8.1 Hz, 2H, NCH_2), 2.20 – 2.13 (m, 2H); ^{13}C NMR (151 MHz, $CDCl_3$) δ 174.5 (C=O), 165.9, 145.9, 138.6, 125.3, 124.6, 112.3, 59.4 (NCH_2), 52.4 (NCH_2), 49.9 (NCH_2), 25.8 (CH_2); TOF-HRMS (ESI) *m/z*: $[M + H]^+$, calcd for $C_{11}H_{14}NO_3$: 208.0968; found: 208.0973.

5-hydroxy-2-(piperidin-1-ylmethyl)-4H-pyran-4-one (4p). Off-white powder; yield: 87%, mp: 134.6-135.8°C, HPLC purity: 98.8%; ^{1}H NMR (600 MHz, $CDCl_3$) δ 7.83 (s, 1H), 6.92 (s, 1H, OH), 6.49 (s, 1H), 3.35 (s, 2H), 2.43 (s, 4H, $2NCH_2$,), 1.66 – 1.55 (m, 4H), 1.43 (s, 2H); ^{13}C NMR (151 MHz, $CDCl_3$) δ 174.4 (C=O), 166.6, 145.8, 138.2, 112.0, 60.6 (NCH_2), 54.7 (2C, NCH_2), 25.9 (2C), 23.9; TOF-HRMS (ESI) *m/z*: $[M + H]^+$, calcd for $C_{11}H_{16}NO_3$: 210.1125; found: 210.1126.

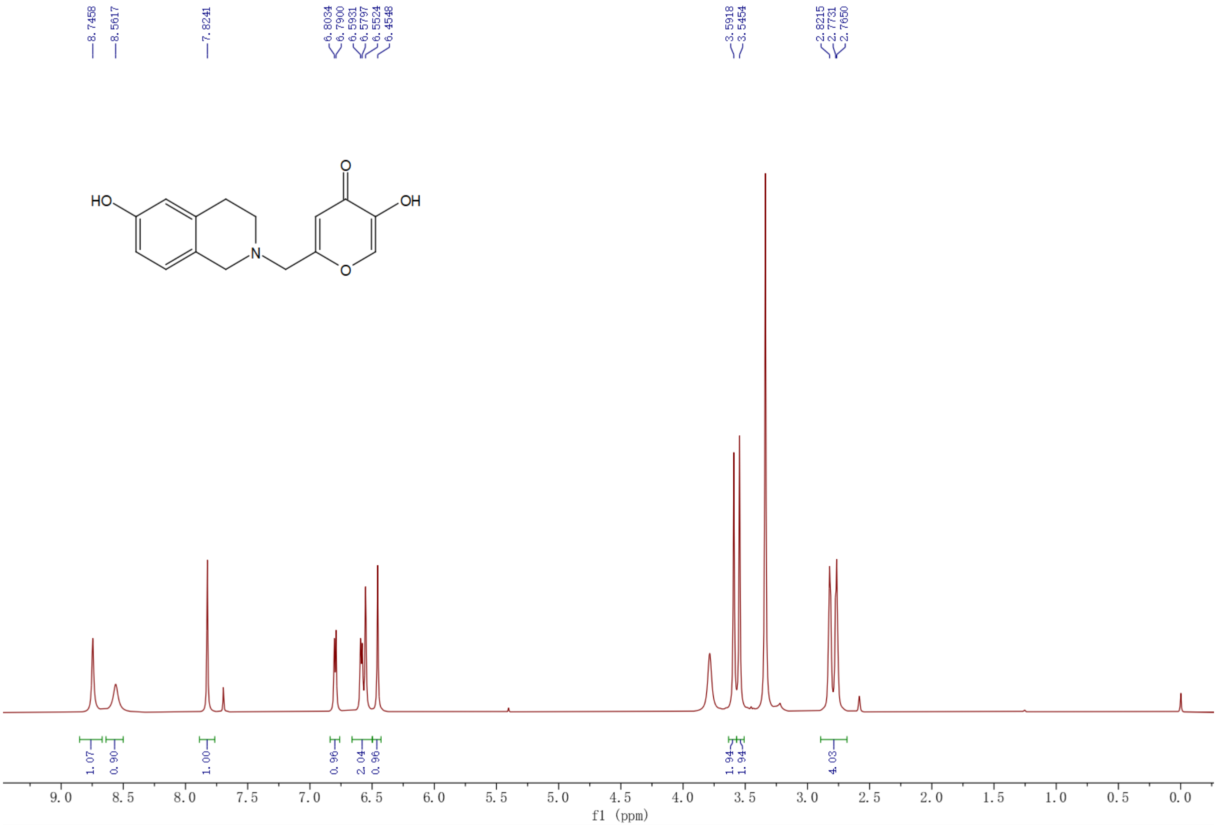

8.7458
8.5617
7.8241
6.8034
6.7900
6.5931
6.5797
6.5524
6.4548
3.5918
3.5454
2.8215
2.7731
2.7650
HO
N
O
OH
O
1.07
0.90
1.00
0.96
2.04
0.96
1.94
1.94
4.03
9.0
8.5
8.0
7.5
7.0
6.5
6.0
5.5
5.0
4.5
4.0
3.5
3.0
2.5
2.0
1.5
1.0
0.5
0.0
f1 (ppm)

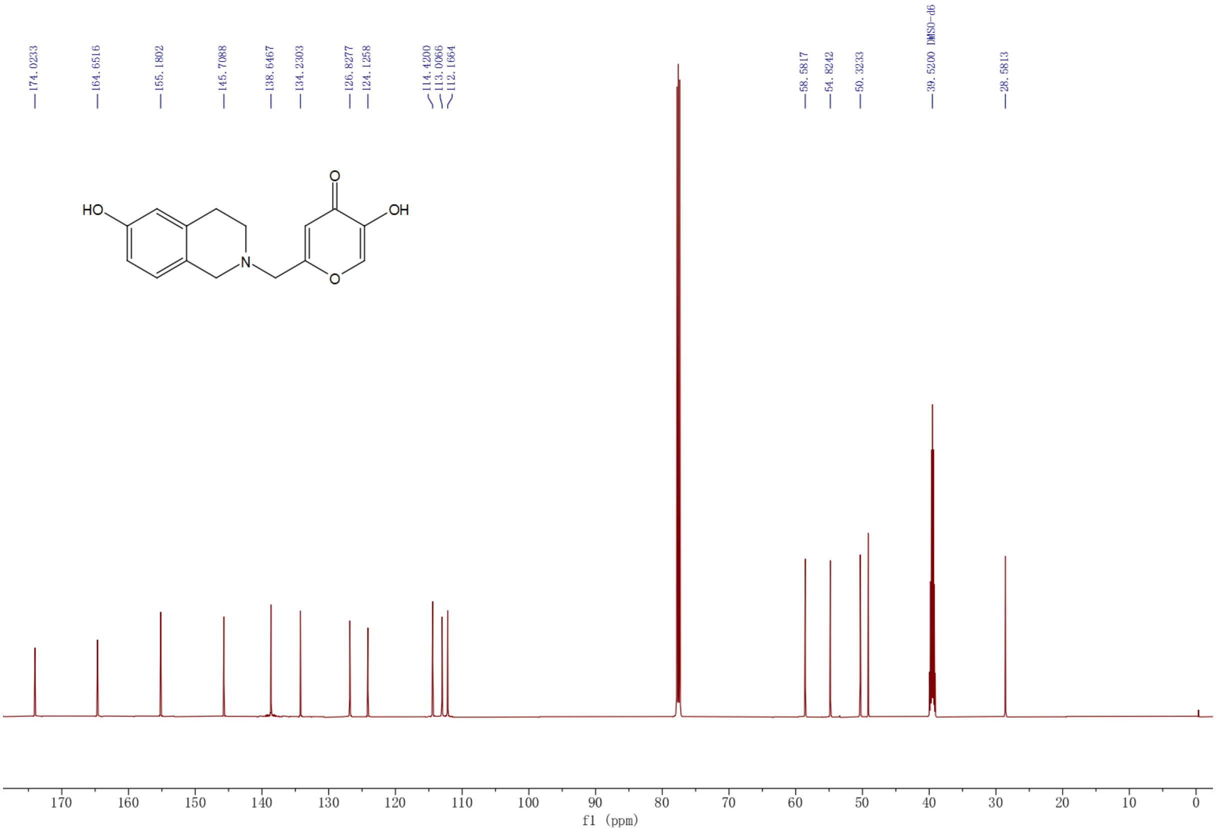

174.0233
164.6516
155.1802
145.7088
138.6467
134.2303
126.8277
124.1258
114.4200
113.0066
112.1664
58.5817
54.8242
50.3233
39.5200 DMSO-d6
28.5813
HO
N
O
OH
O
170
160
150
140
130
120
110
100
90
80
70
60
50
40
30
20
10
0
f1 (ppm)

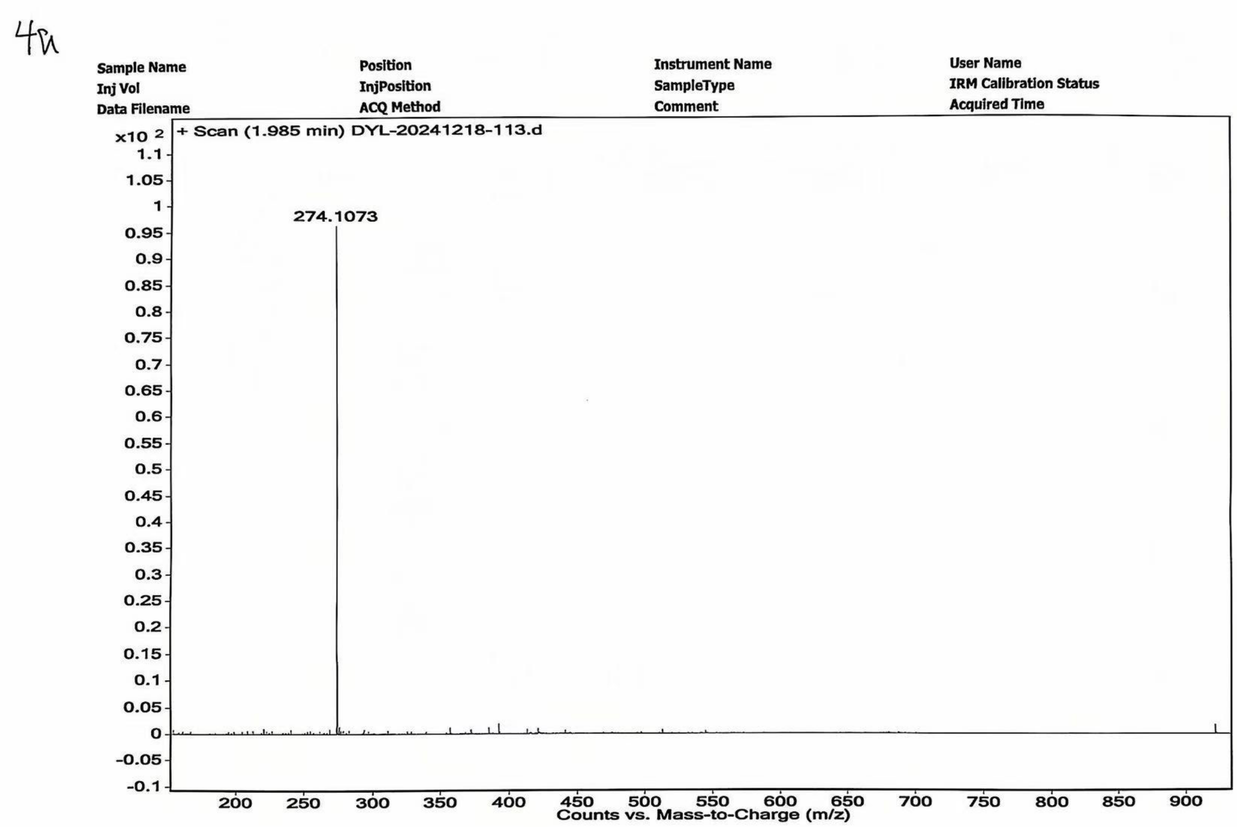

Figure S1. ^{1}H-NMR, ^{13}C-NMR and HRMS (ESI) spectra of compound **4a**.

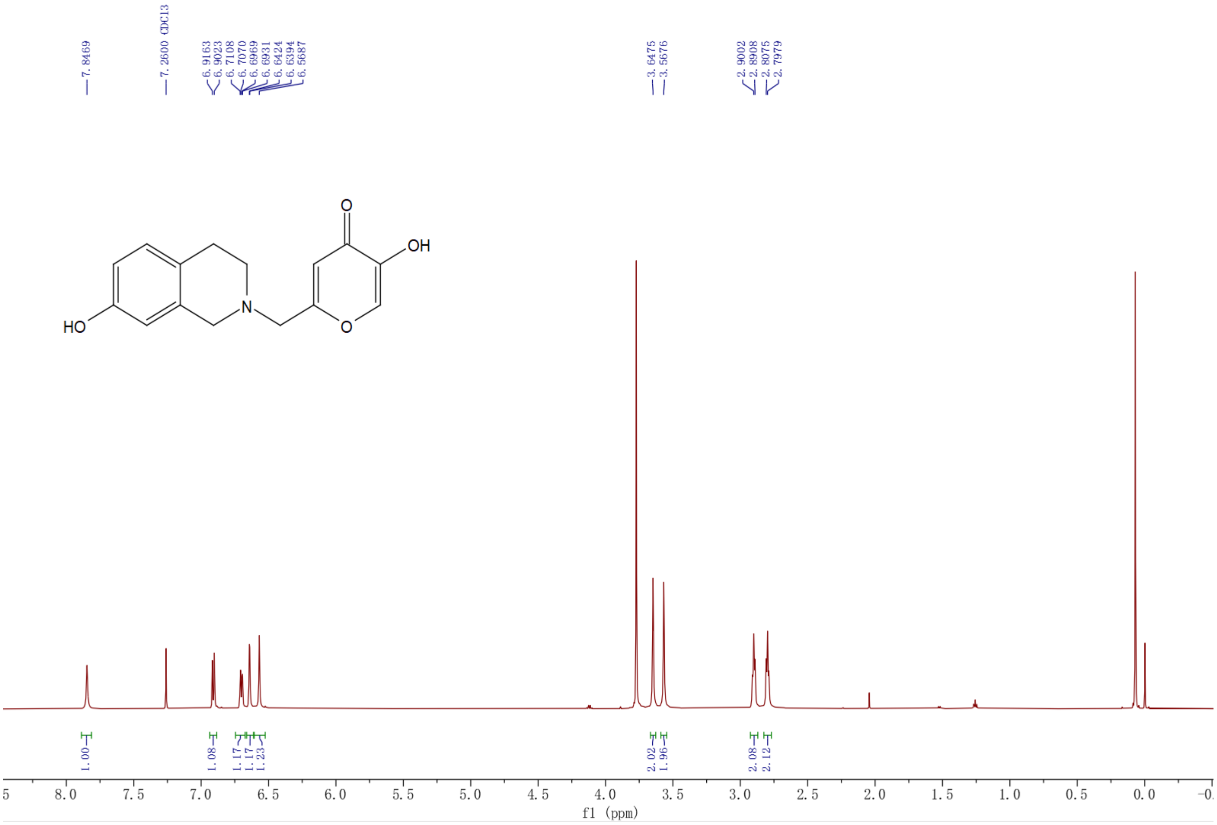
7.8469
7.2600 CDCl3
6.9163
6.9023
6.7108
6.7070
6.6969
6.6931
6.6424
6.6394
6.5687
3.6475
3.5676
2.9002
2.8908
2.8075
2.7979
HO
N
O
OH
O
1.00
1.08
1.17
1.17
1.23
2.02
1.96
2.08
2.12
f1 (ppm)

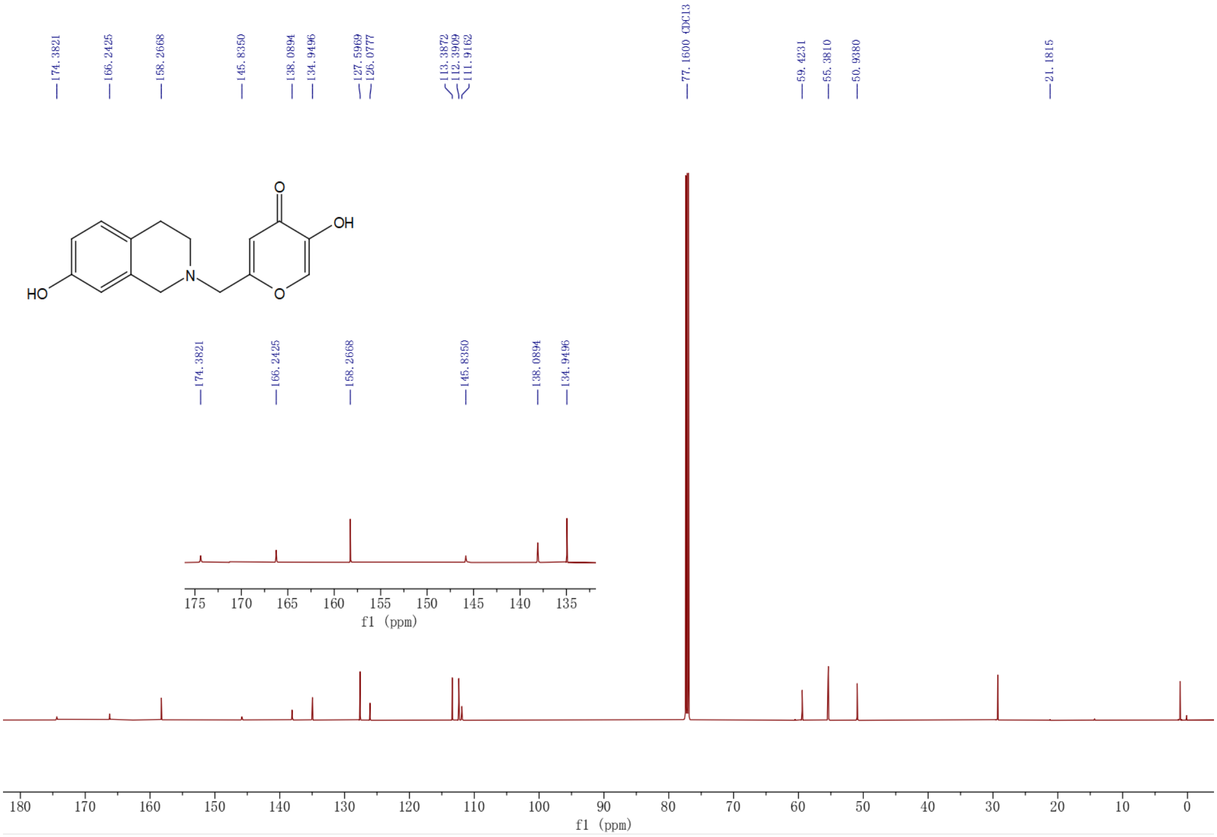
174.3821
166.2425
158.2668
145.8350
138.0894
134.9496
127.5969
126.0777
113.3872
112.3909
111.9162
77.1600 CDCl3
59.4231
55.3810
50.9380
21.1815
HO
N
O
OH
O
f1 (ppm)

4b

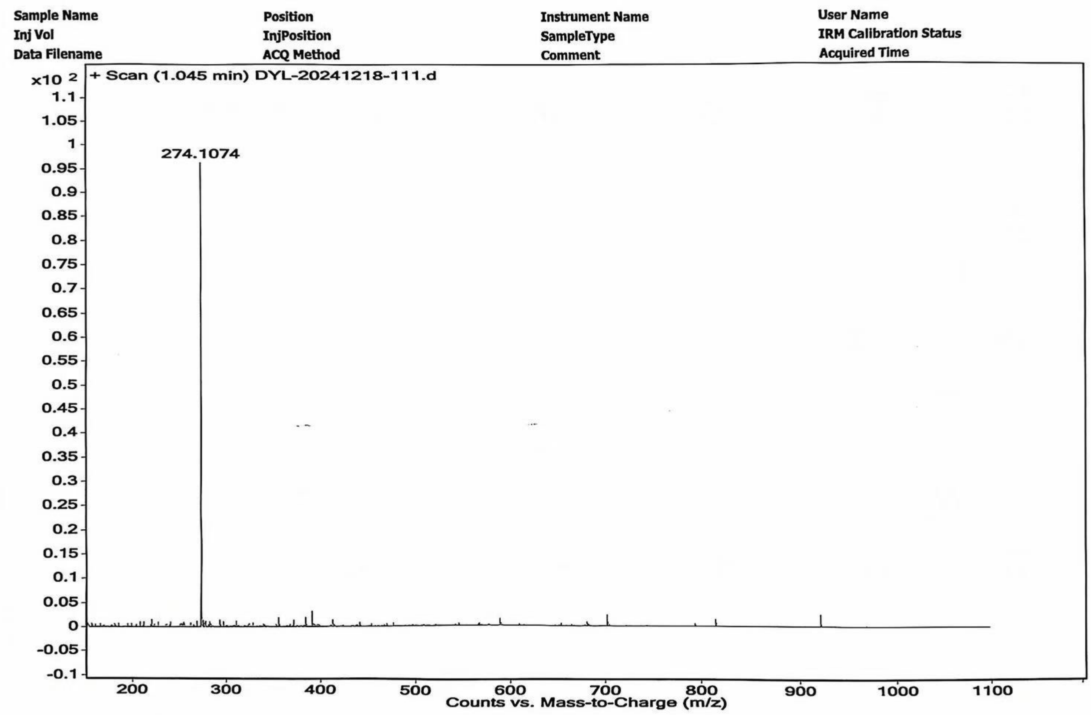

Figure S2. ^{1}H-NMR, ^{13}C-NMR and HRMS (ESI) spectra of compound **4b.**

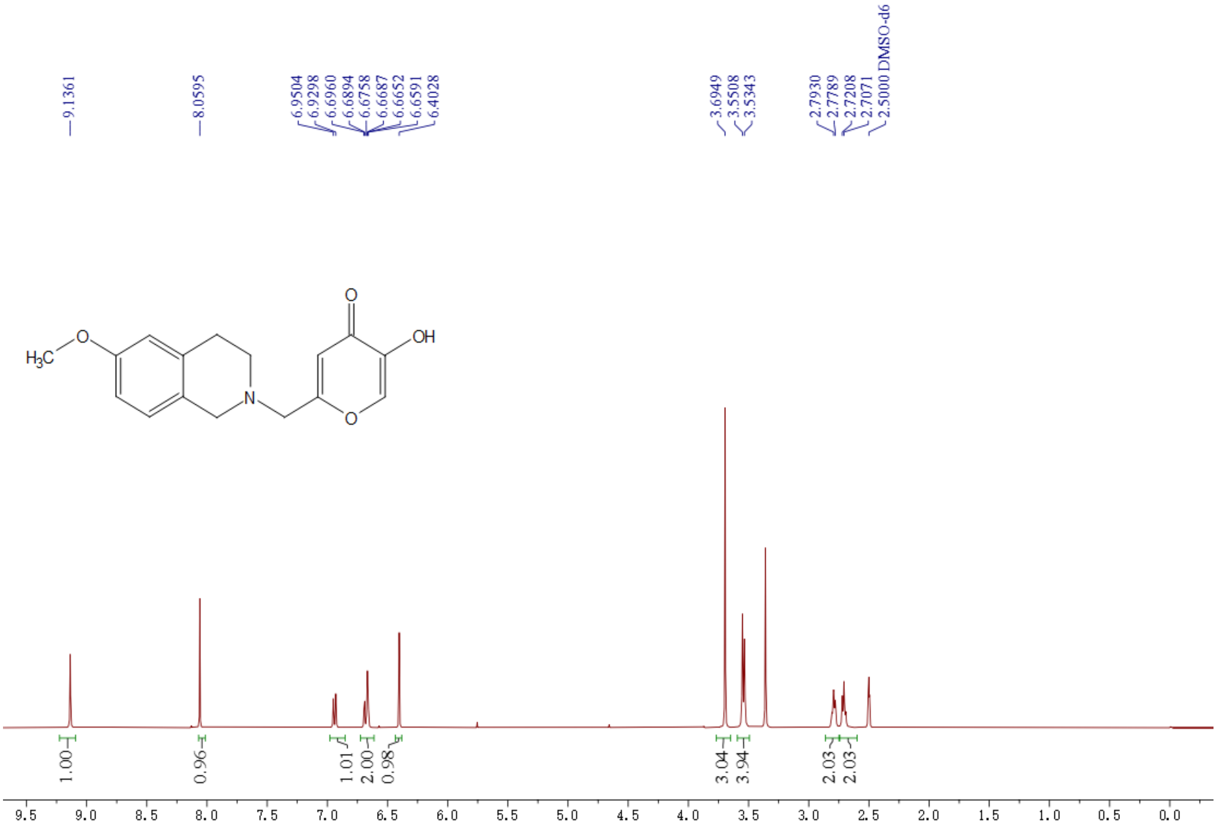
9.1361
8.0595
6.9504
6.9298
6.6960
6.6894
6.6758
6.6687
6.6652
6.6591
6.4028
3.6949
3.5508
3.5343
2.7930
2.7789
2.7208
2.7071
2.5000 DMSO-d6
1.00
0.96
1.01
2.00
0.98
3.04
3.94
2.03
2.03

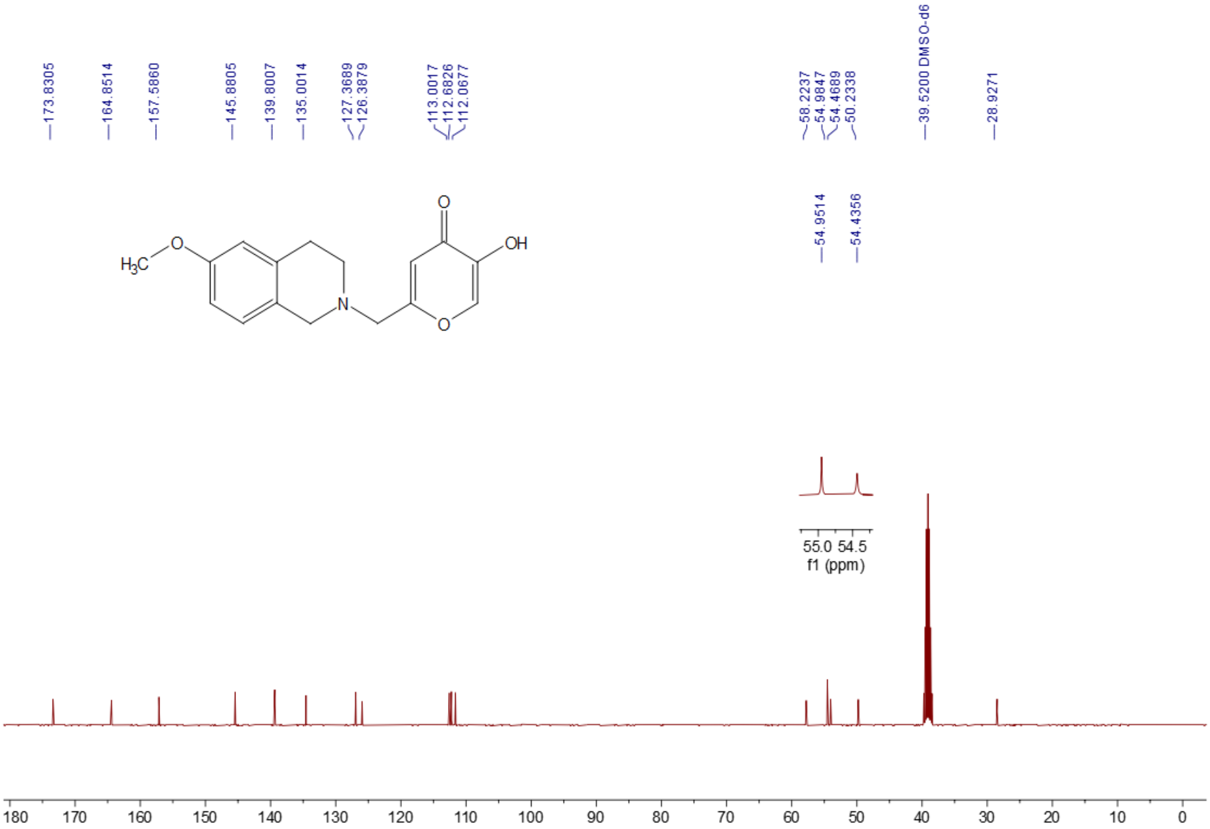
173.8305
164.8514
157.5860
145.8805
139.8007
135.0014
127.3689
126.3879
113.0017
112.6826
112.0677
58.2237
54.9847
54.4689
50.2338
39.5200 DMSO-d6
28.9271
54.9514
54.4356
f1 (ppm)

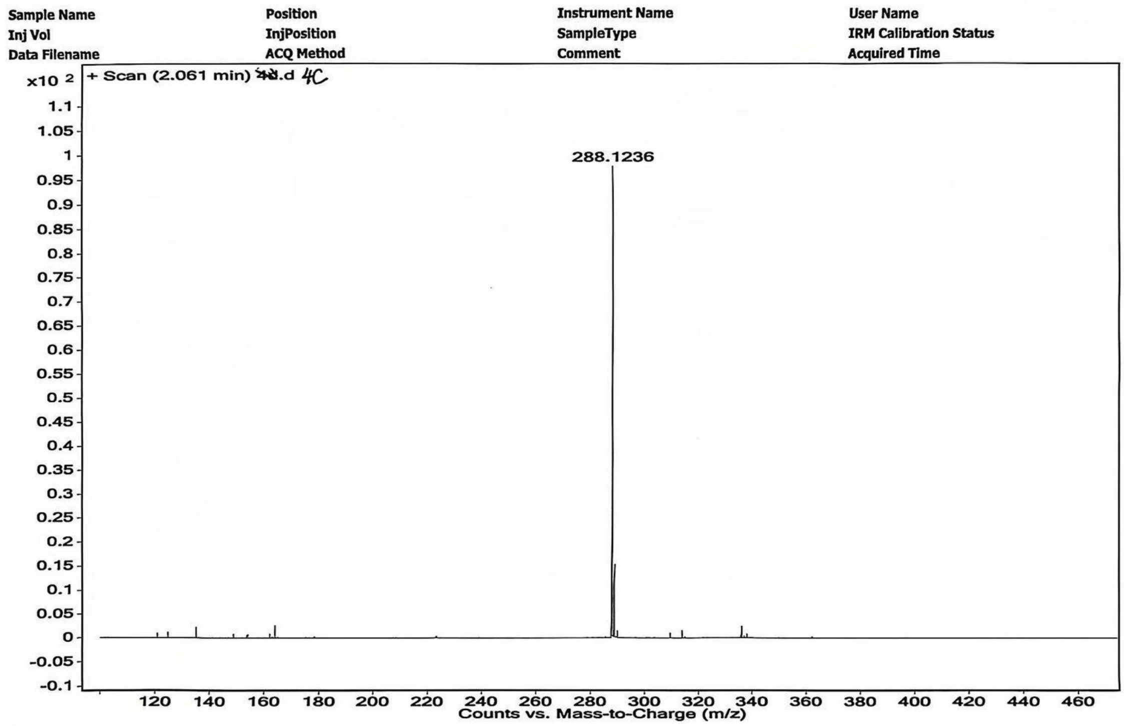
Sample Name
Inj Vol
Data Filename
Position
InjPosition
ACQ Method
Instrument Name
SampleType
Comment
User Name
IRM Calibration Status
Acquired Time
x10 2
+ Scan (2.061 min)
4C
288.1236
Counts vs. Mass-to-Charge (m/z)

Analysis

<Sample message>

Sample : 4C
SampleID : 4C
File name : 4C.lcd
Mothod File : method-3.lcm
Batch File :
Samplebottle : 1-23
Injection Volume : 20 uL
Analysis Date : 2025/9/12 12:22:04
Administrator Manipulate Date : 2025/9/15 16:16:46

Sample Type : Standard
Analyser : System Administrator
Handler : System Administrator

<Chromatogram>

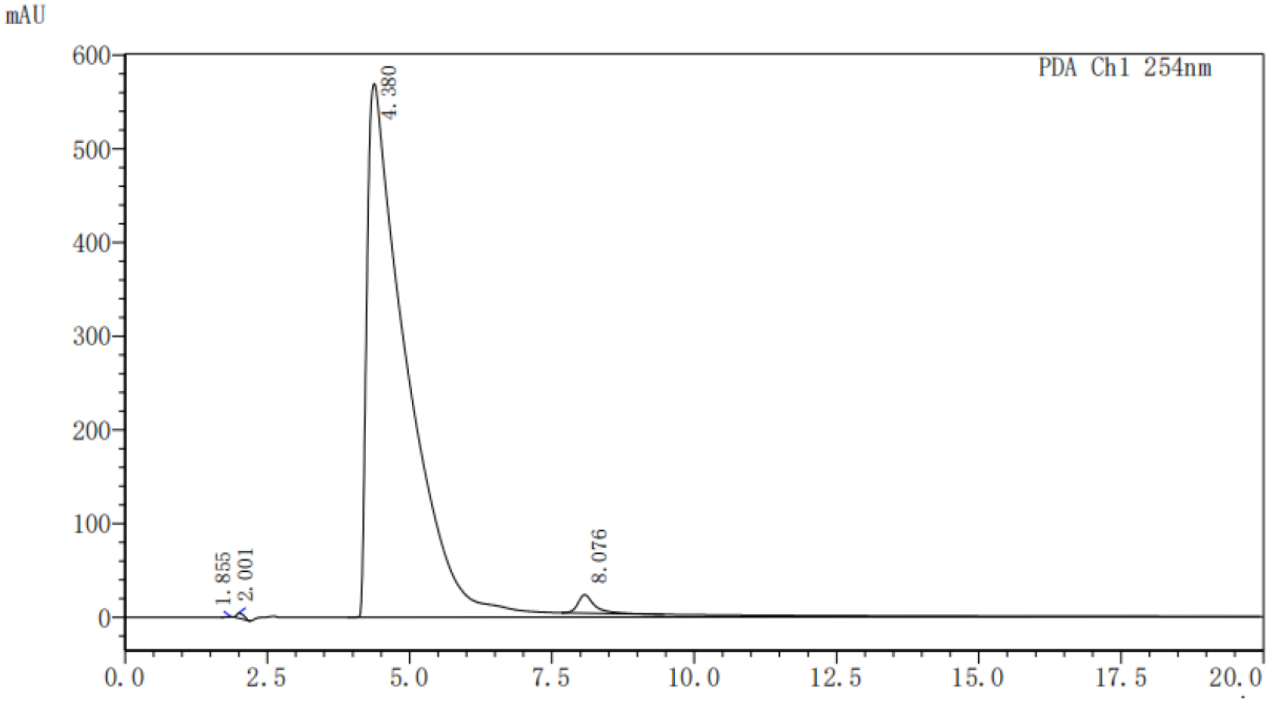

<Peak Table>

PDA Ch1 254n m

Peak	t_R	Area	Height	Concentration	Unit	Mark	Compound name
1	1.855	1356	276	0.000			
2	2.001	46256	5321	0.000			
3	4.380	28376960	569494	0.000			
4	8.076	408803	19742	0.000			
Total		28833375	594833				

Figure S3. ^{1}H-NMR, ^{13}C-NMR, HRMS (ESI) and HPLC spectra of compound **4c.**

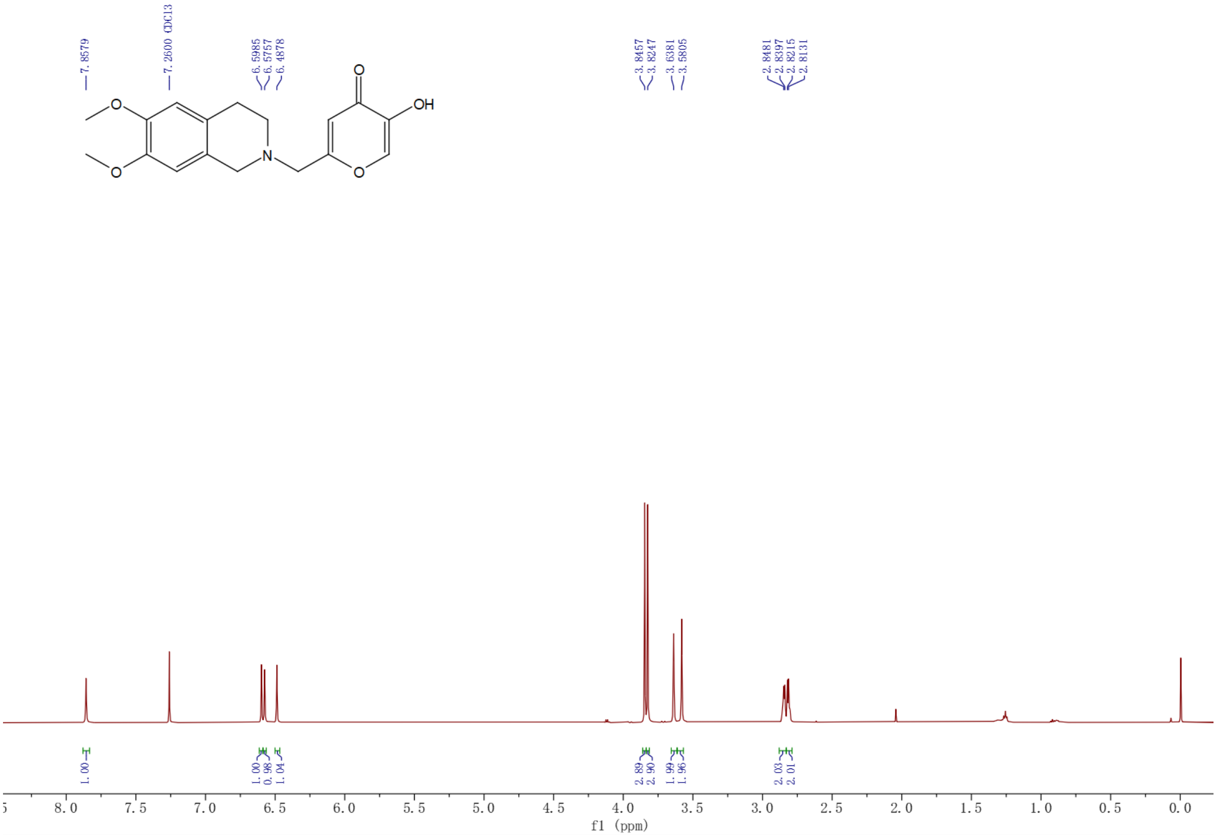
7.8579
7.2600 CDCl3
6.5985
6.5757
6.4878
3.8457
3.8247
3.6381
3.5805
2.8481
2.8397
2.8215
2.8131
O
OH
N
1.00
1.00
0.98
1.04
2.89
2.90
1.99
1.96
2.03
2.01
f1 (ppm)

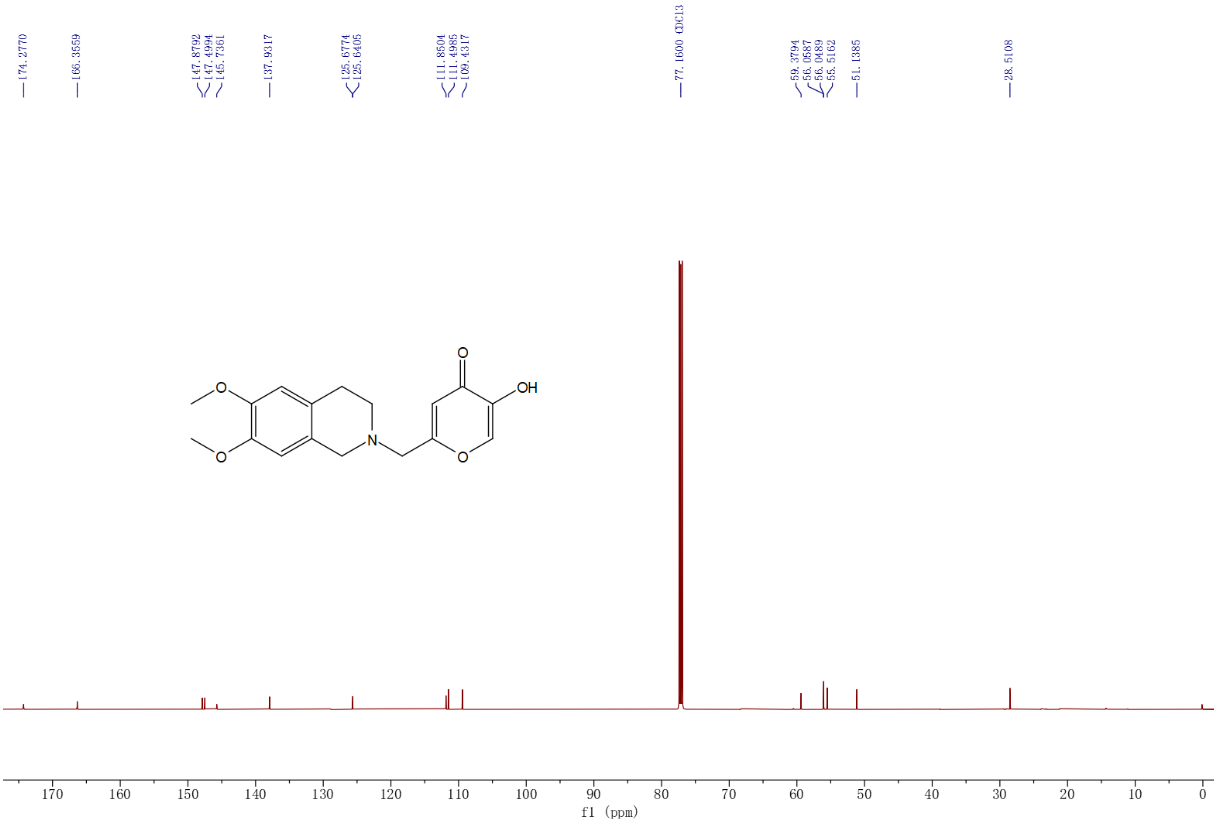
174.2770
166.3559
147.8792
147.4994
145.7361
137.9317
125.6774
125.6405
111.8504
111.4985
109.4317
77.1600 CDCl3
59.3794
56.0587
56.0489
55.5162
51.1385
28.5108
O
OH
N
f1 (ppm)

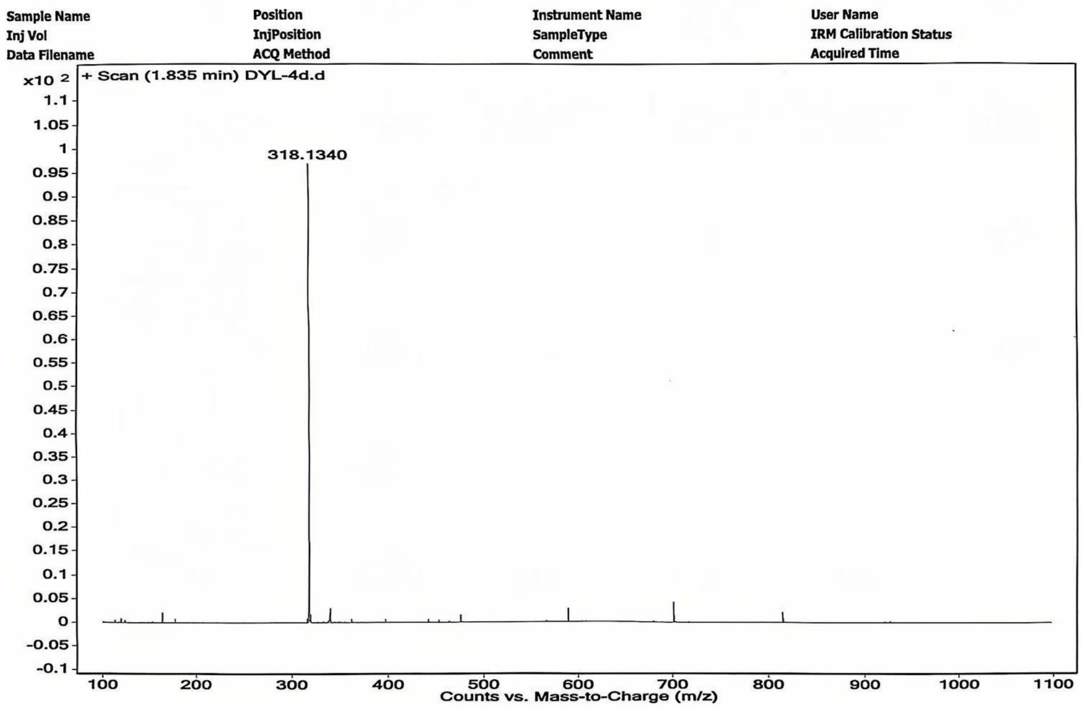

Figure S4. ^{1}H-NMR, ^{13}C-NMR and HRMS (ESI) spectra of compound **4d**.

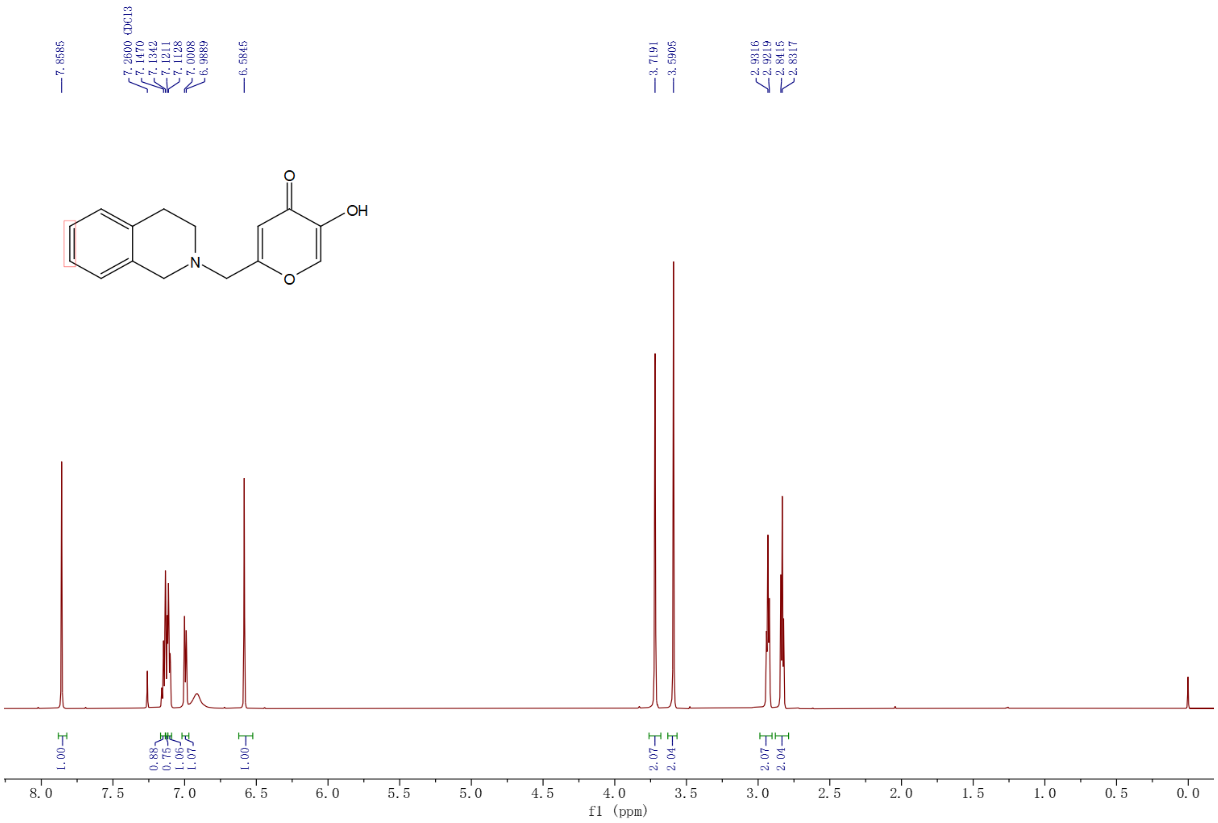

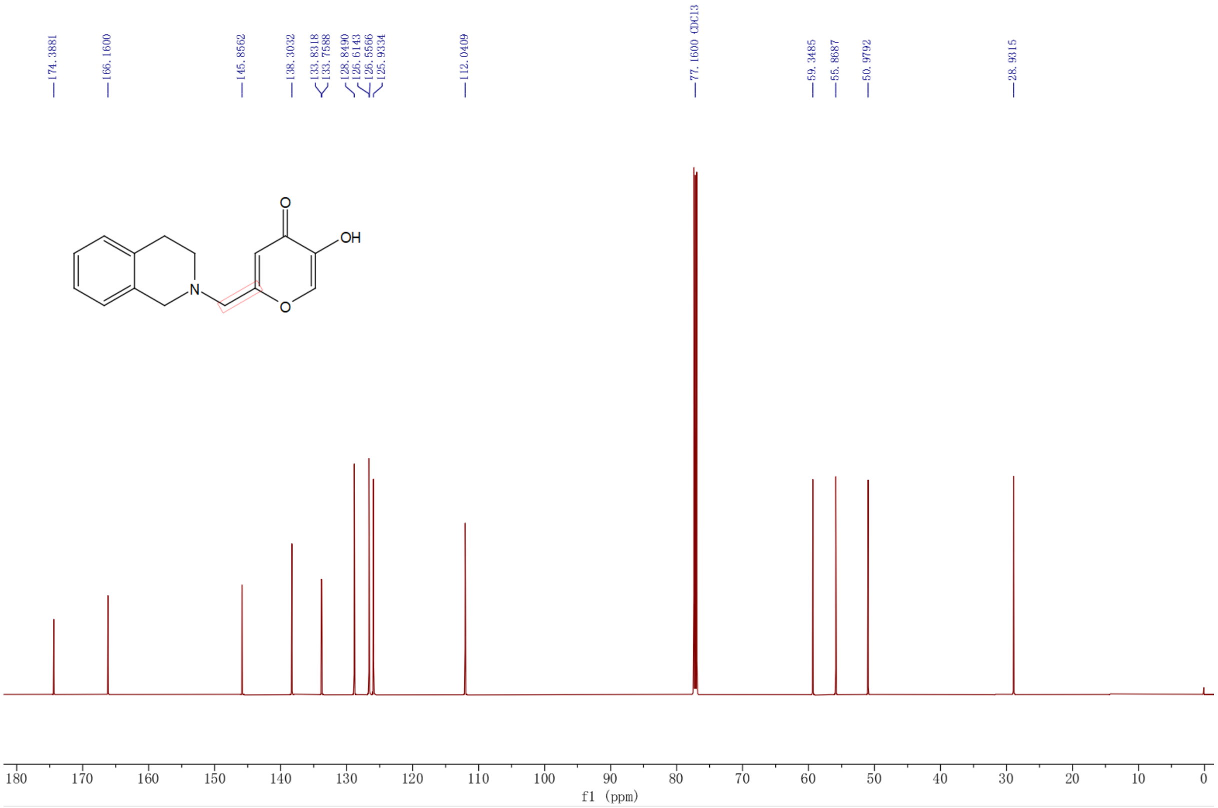

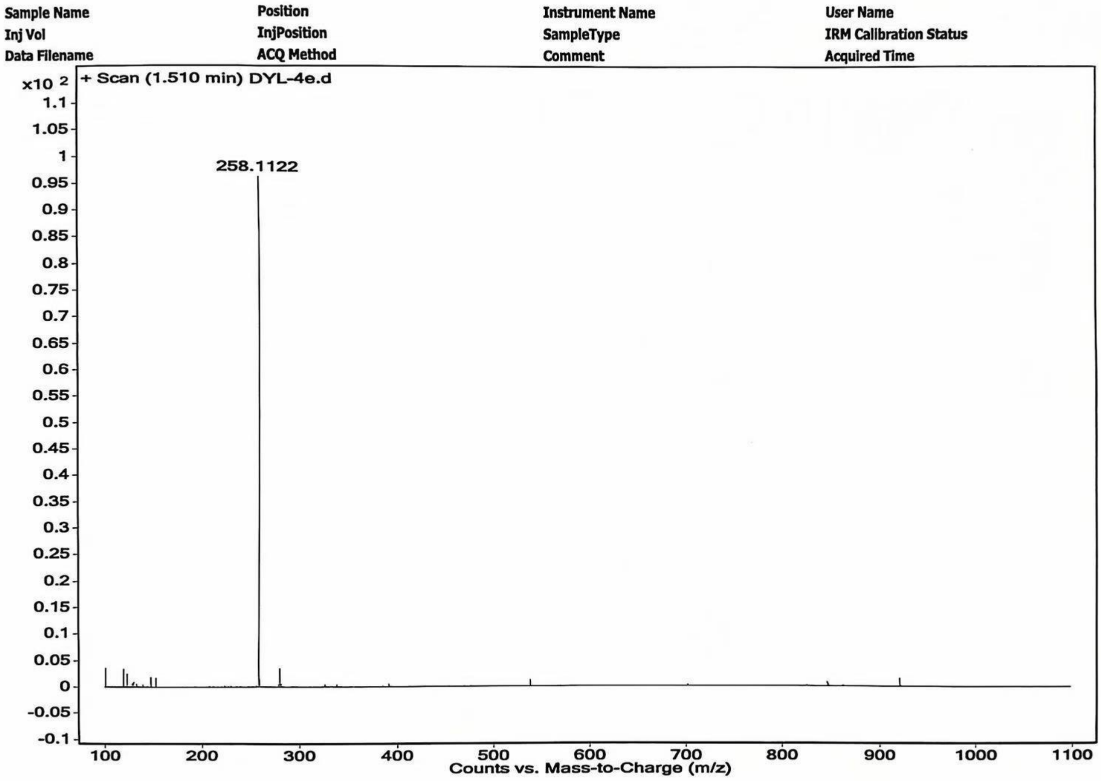

Figure S5. ^{1}H-NMR, ^{13}C-NMR and HRMS (ESI) spectra of compound **4e**.

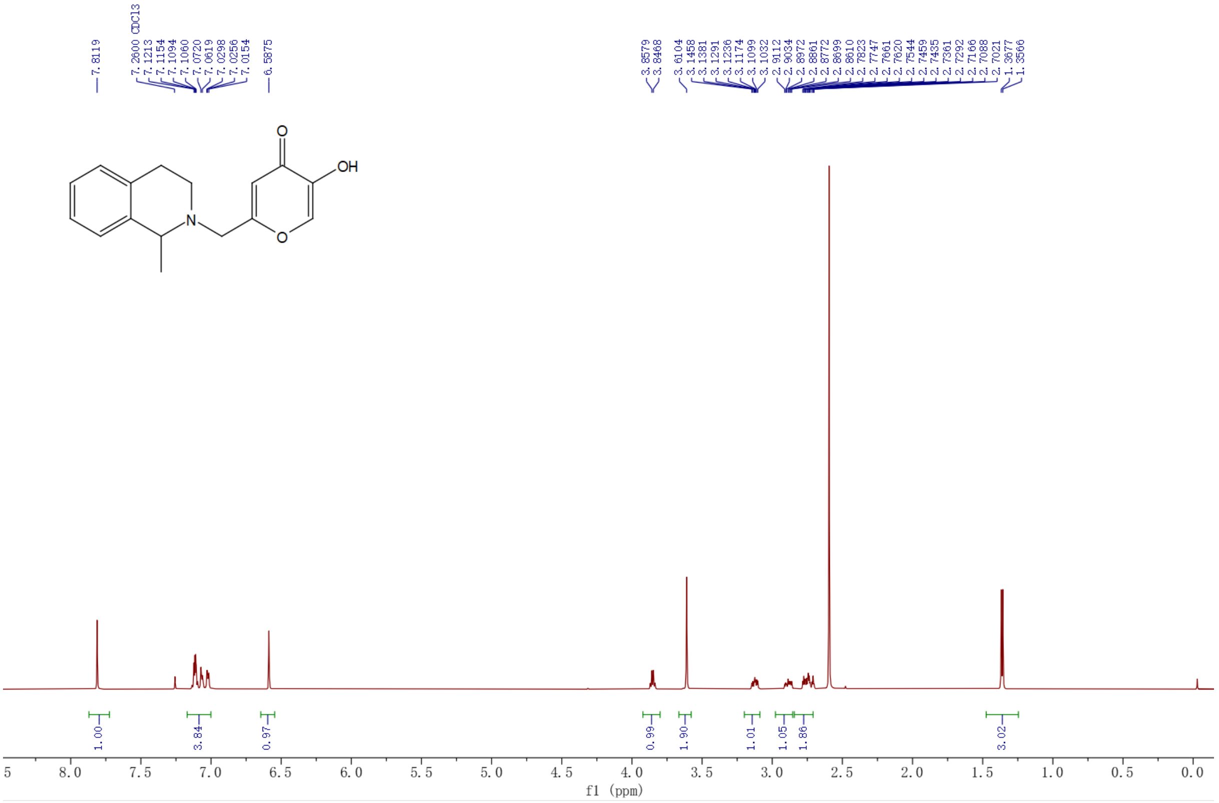

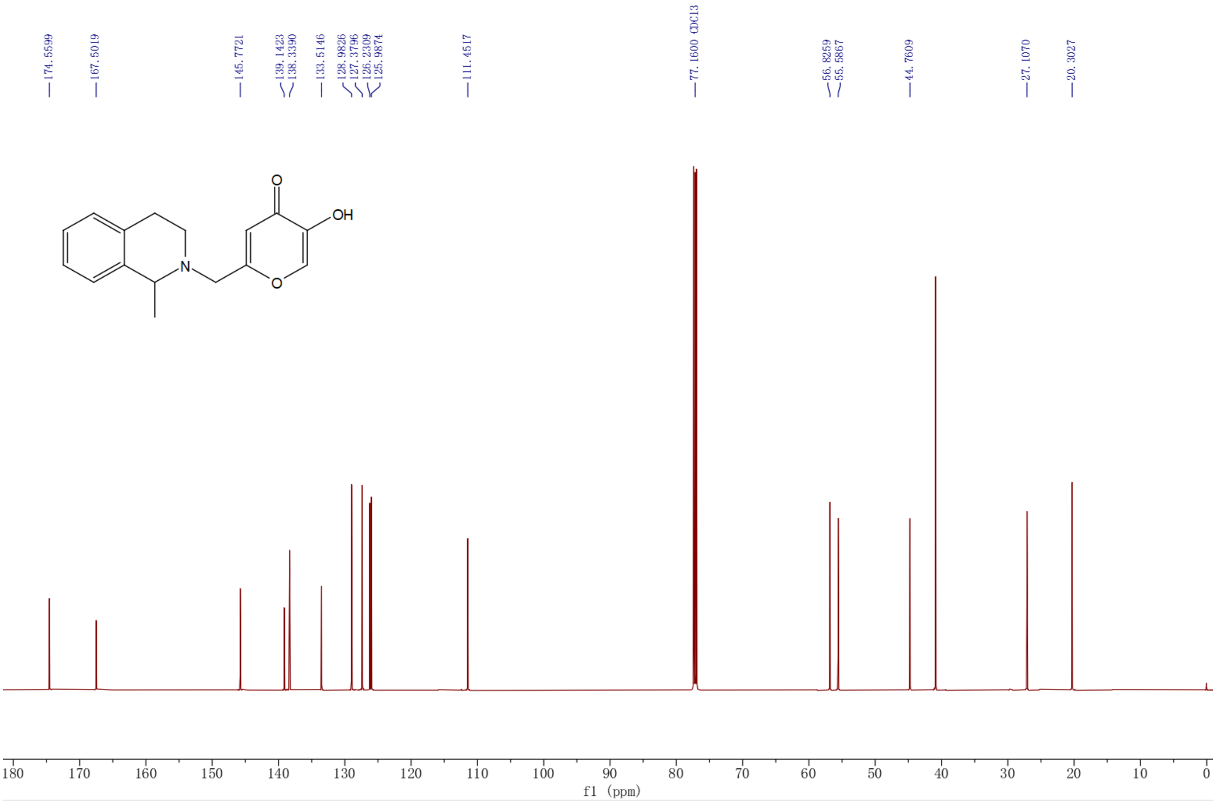

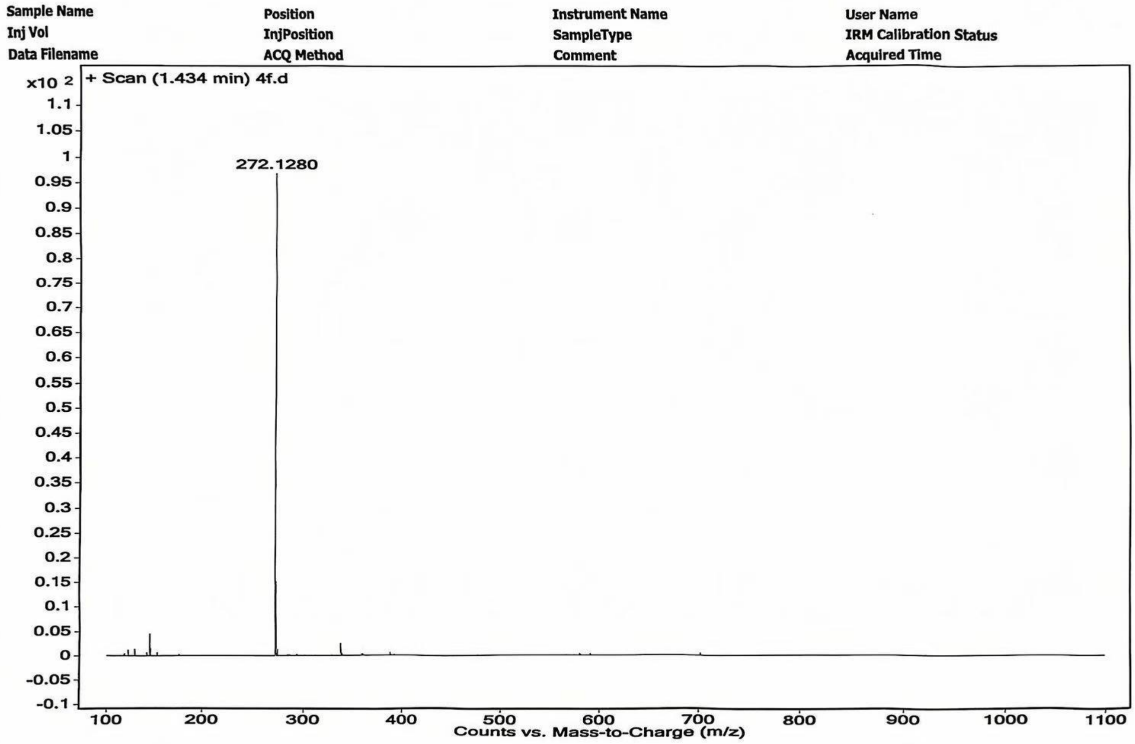

Figure S6. ^{1}H-NMR, ^{13}C-NMR and HRMS (ESI) spectra of compound **4f**.

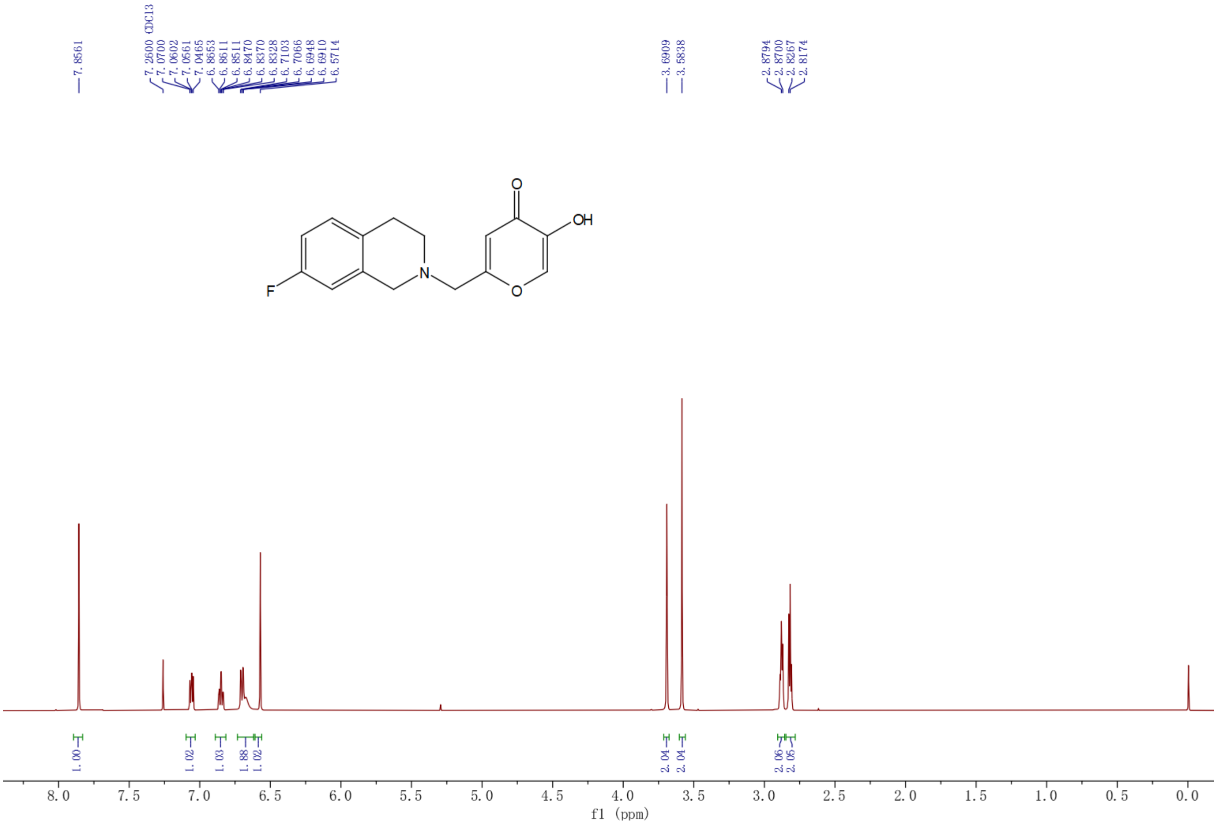

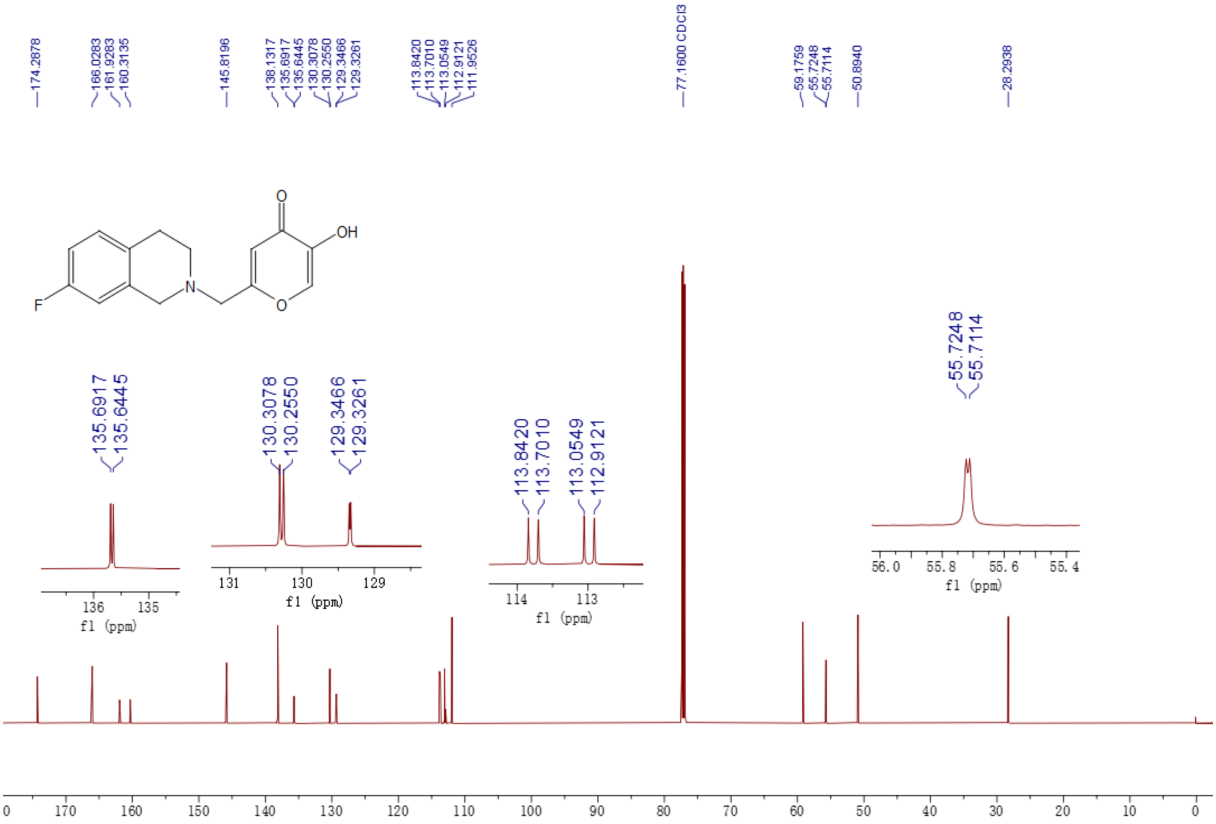

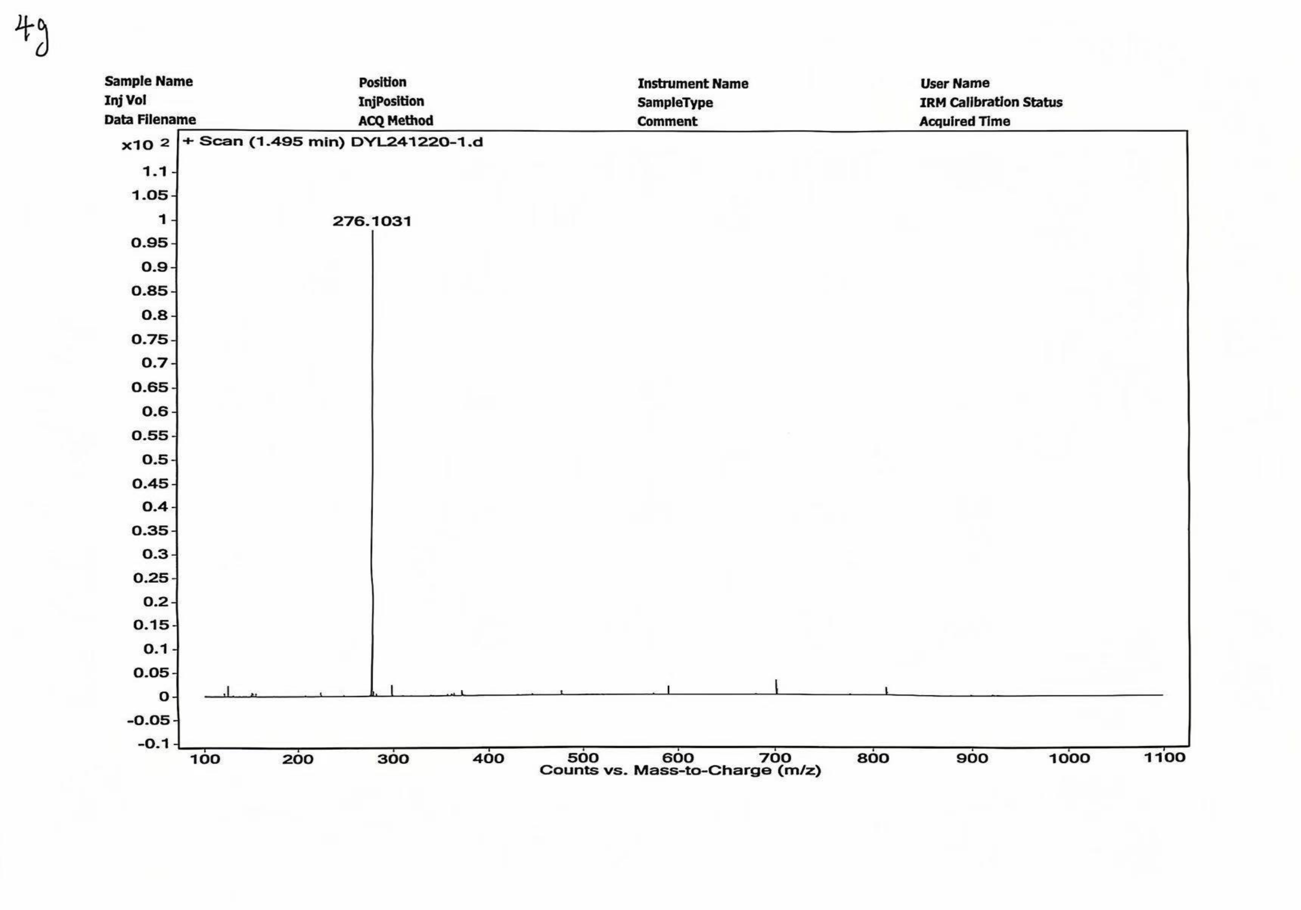

Figure S7. ^{1}H-NMR, ^{13}C-NMR and HRMS (ESI) spectra of compound **4g**.

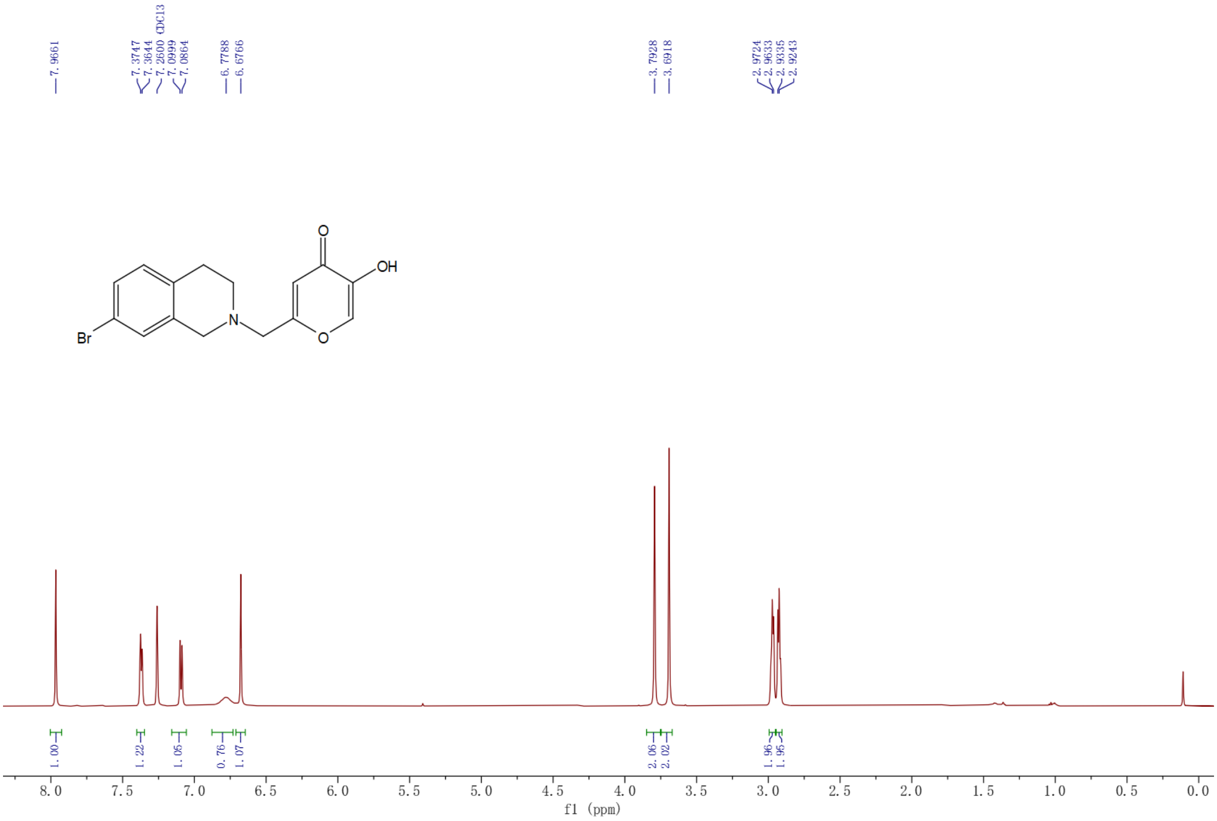
7.9661
7.3747
7.3644
7.2600 CDCl3
7.0999
7.0864
6.7788
6.6766
3.7928
3.6918
2.9724
2.9633
2.9335
2.9243
OH
O
Br
N
1.00
1.22
1.05
0.76
1.07
2.06
2.02
1.96
1.95
f1 (ppm)

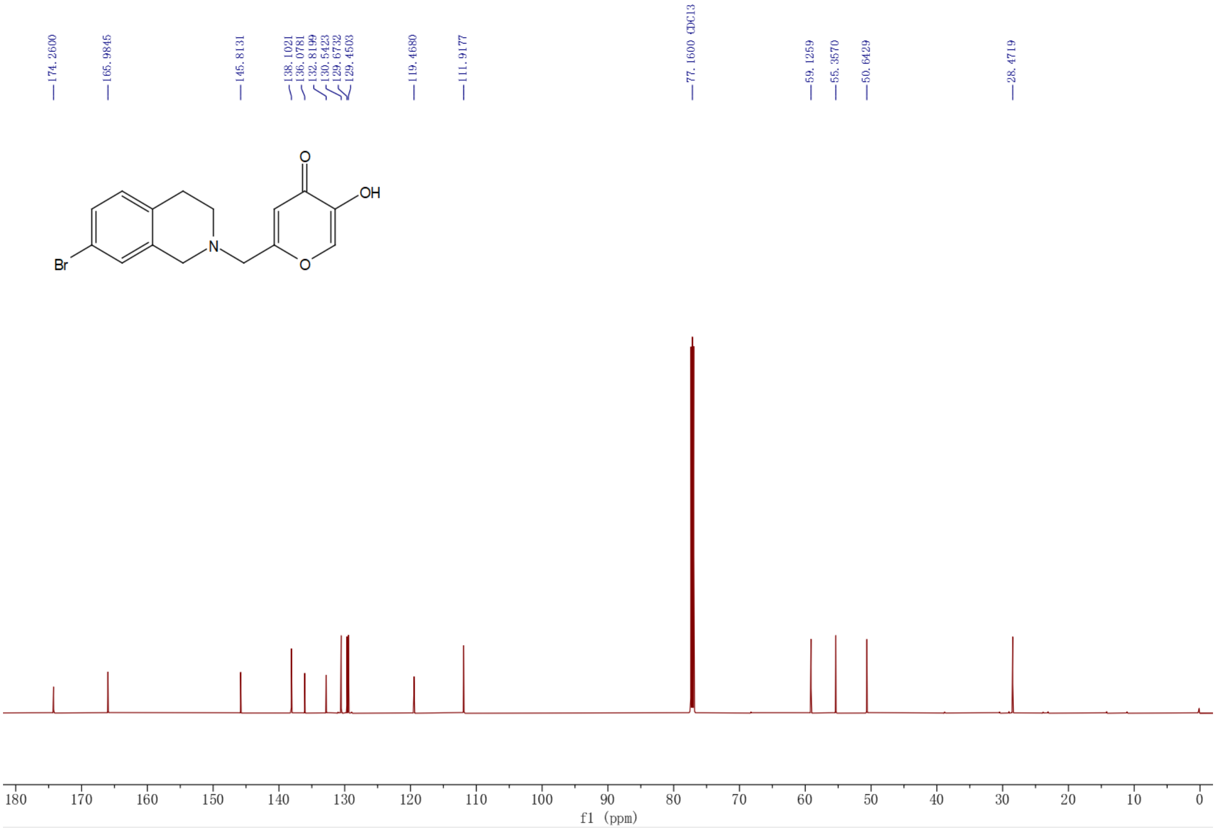
174.2600
165.9845
145.8131
138.1021
136.0781
132.8199
130.5423
129.6732
129.4503
119.4680
111.9177
77.1600 CDCl3
59.1259
55.3570
50.6429
28.4719
OH
O
Br
N
f1 (ppm)

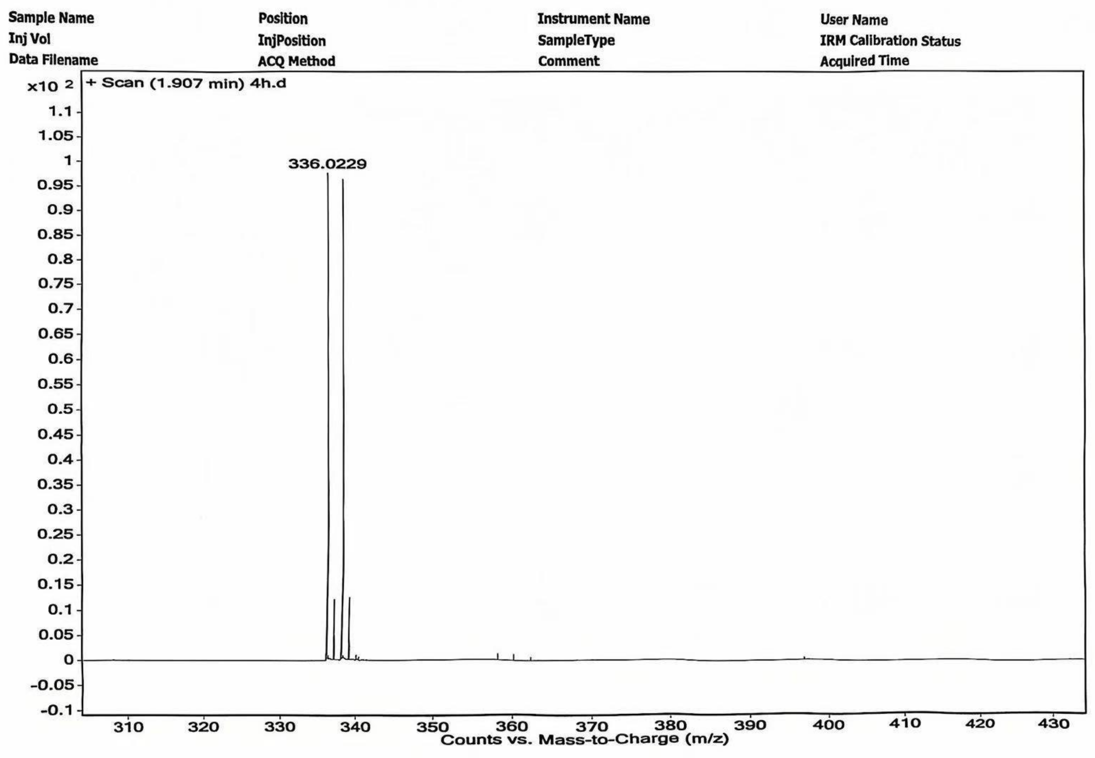

Figure S8. ^{1}H-NMR, ^{13}C-NMR and HRMS (ESI) spectra of compound **4h**.

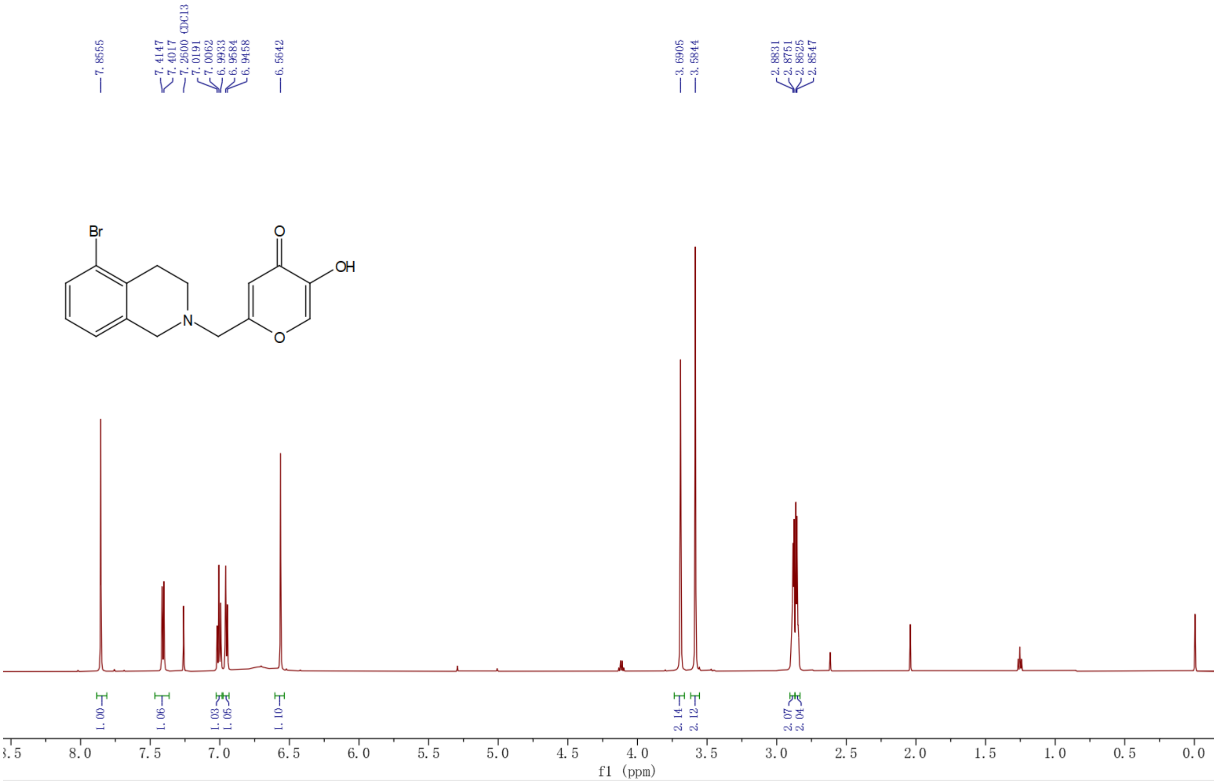
7.8555
7.4147
7.4017
7.2600 CDCl3
7.0191
7.0062
6.9933
6.9584
6.9458
6.5642
3.6905
3.5844
2.8831
2.8751
2.8625
2.8547
Br
O
OH
N
O
1.00
1.06
1.03
1.05
1.10
2.14
2.12
2.07
2.04
f1 (ppm)

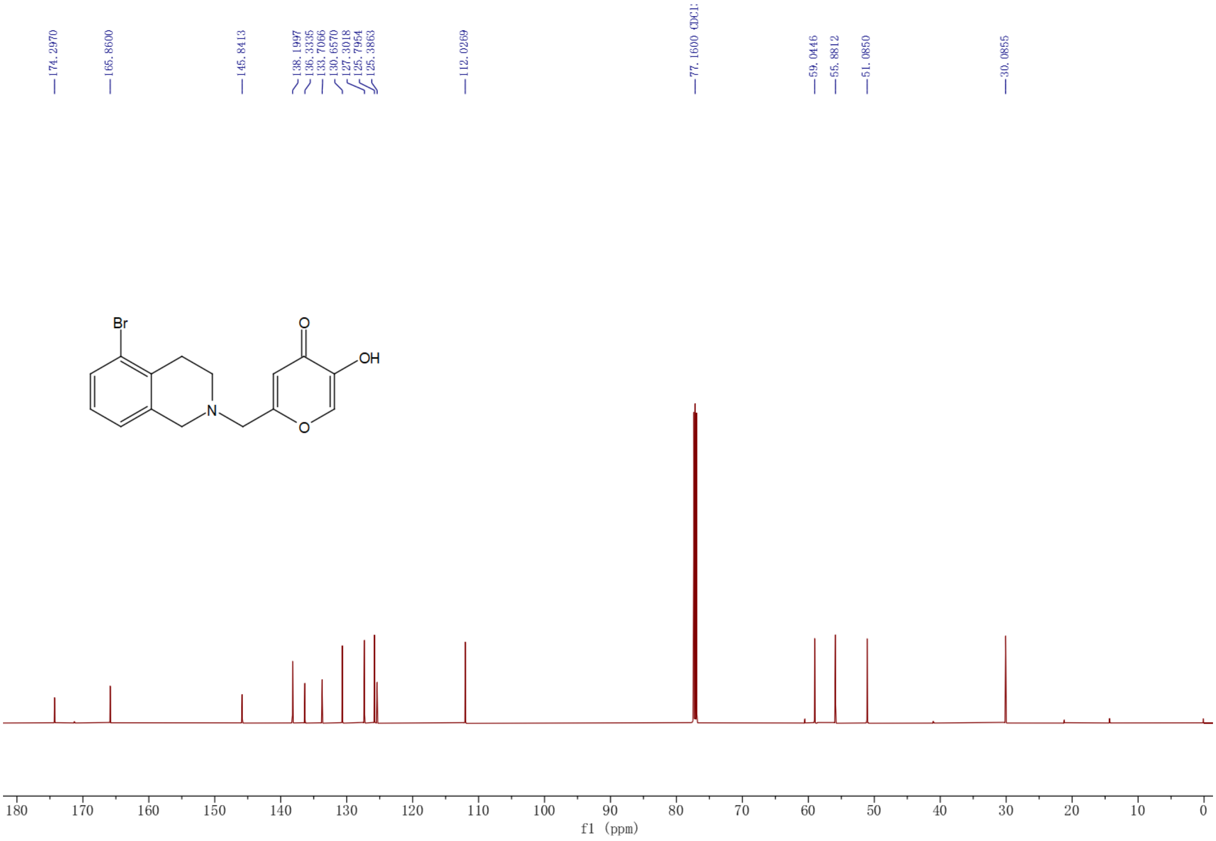
174.2970
165.8600
145.8413
138.1997
136.3335
133.7066
130.6570
127.3018
125.7954
125.3863
112.0269
77.1600 CDCl3
59.0446
55.8812
51.0850
30.0855
Br
O
OH
N
O
f1 (ppm)

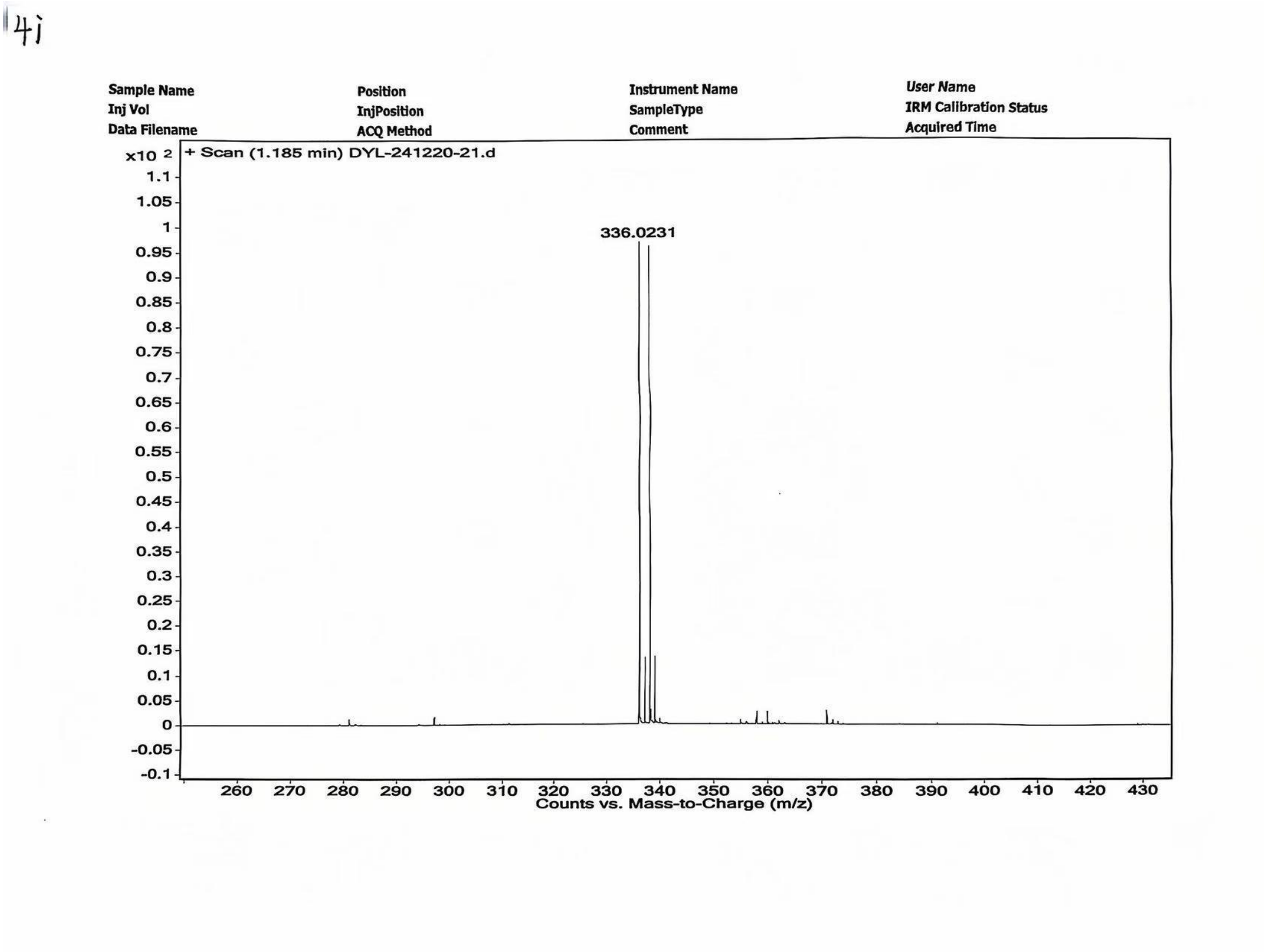

Figure S9. ^{1}H-NMR, ^{13}C-NMR and HRMS (ESI) spectra of compound **4i**.

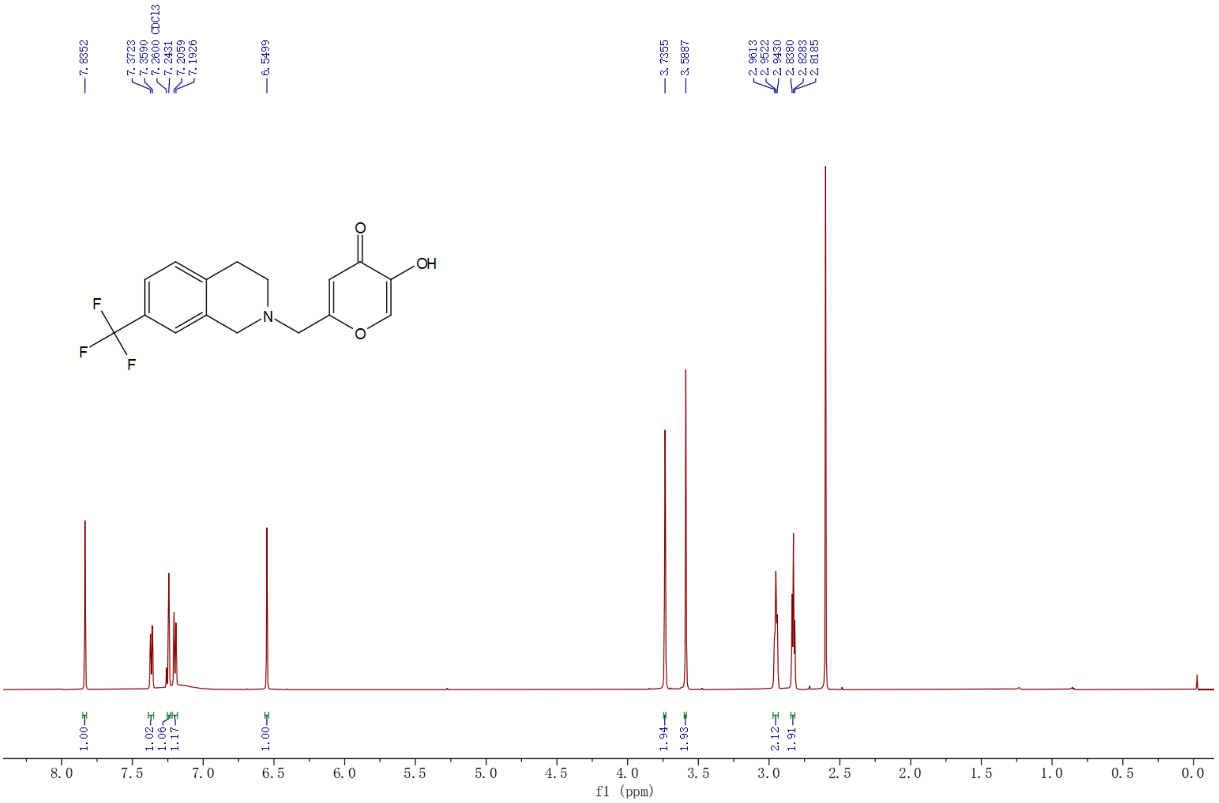

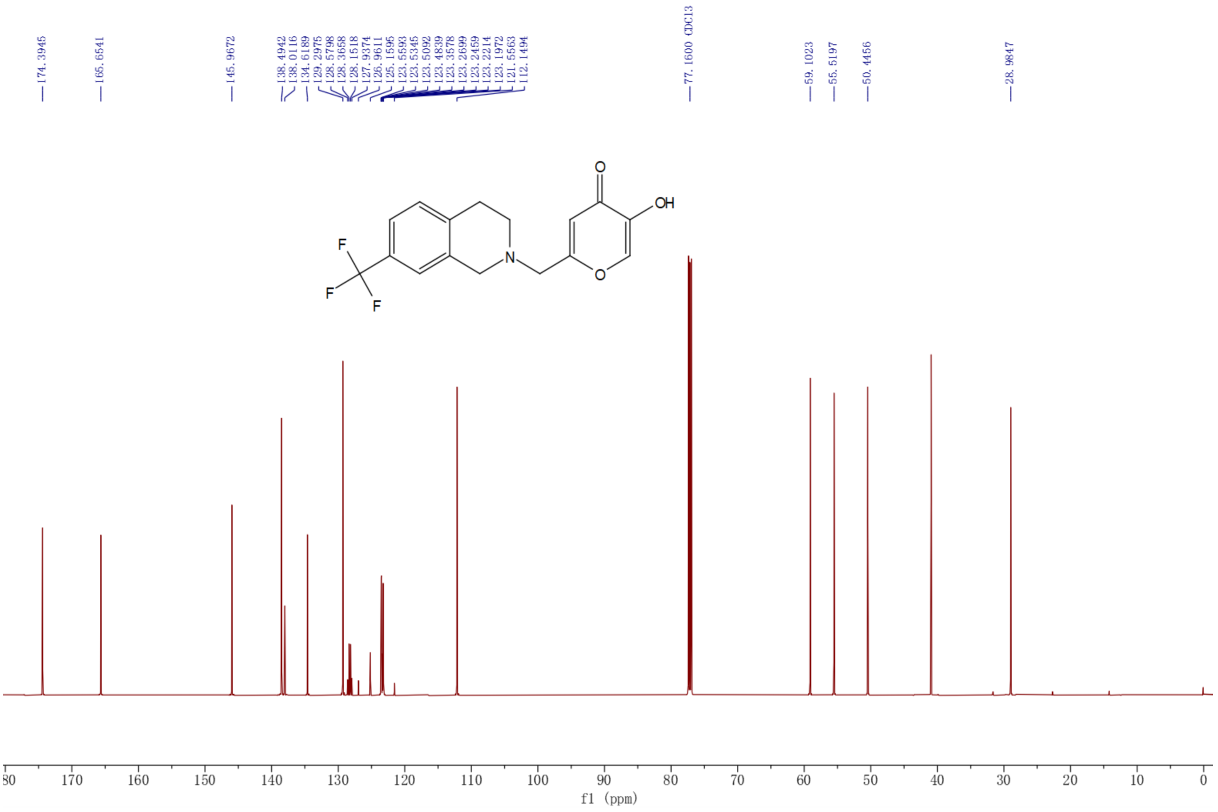

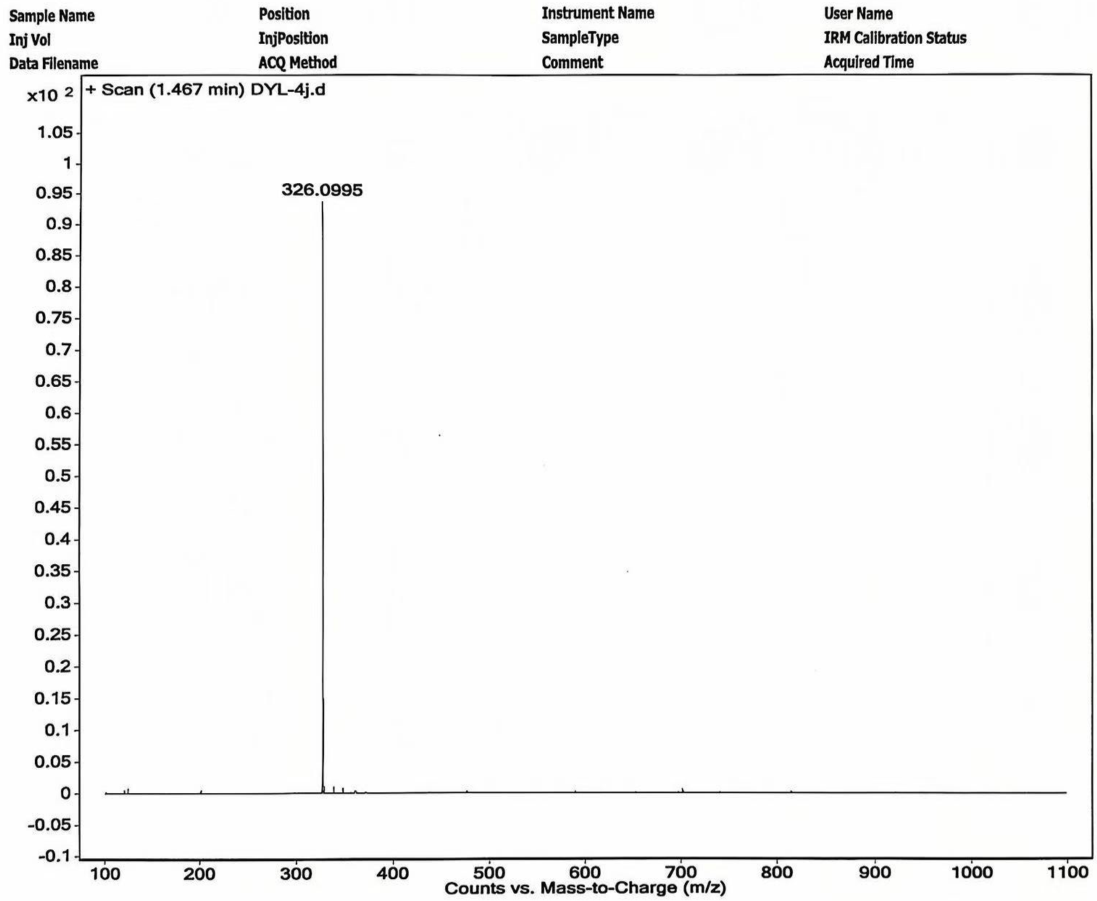

Figure S10. ^{1}H-NMR, ^{13}C-NMR and HRMS (ESI) spectra of compound **4j**.

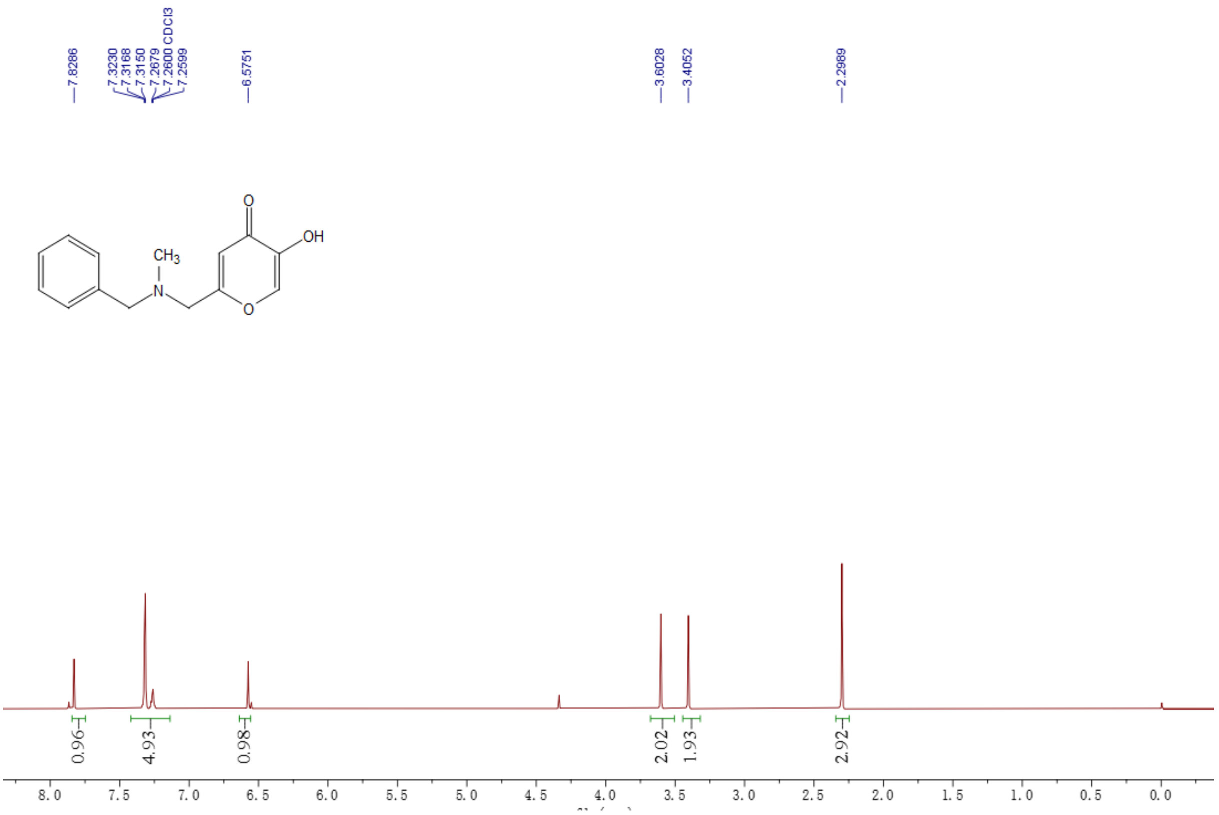
7.8286
7.3230
7.3168
7.3150
7.2679
7.2600 CDCl3
7.2599
6.5751
3.6028
3.4052
2.2989
O
OH
CH3
N
O
0.96
4.93
0.98
2.02
1.93
2.92
8.0
7.5
7.0
6.5
6.0
5.5
5.0
4.5
4.0
3.5
3.0
2.5
2.0
1.5
1.0
0.5
0.0

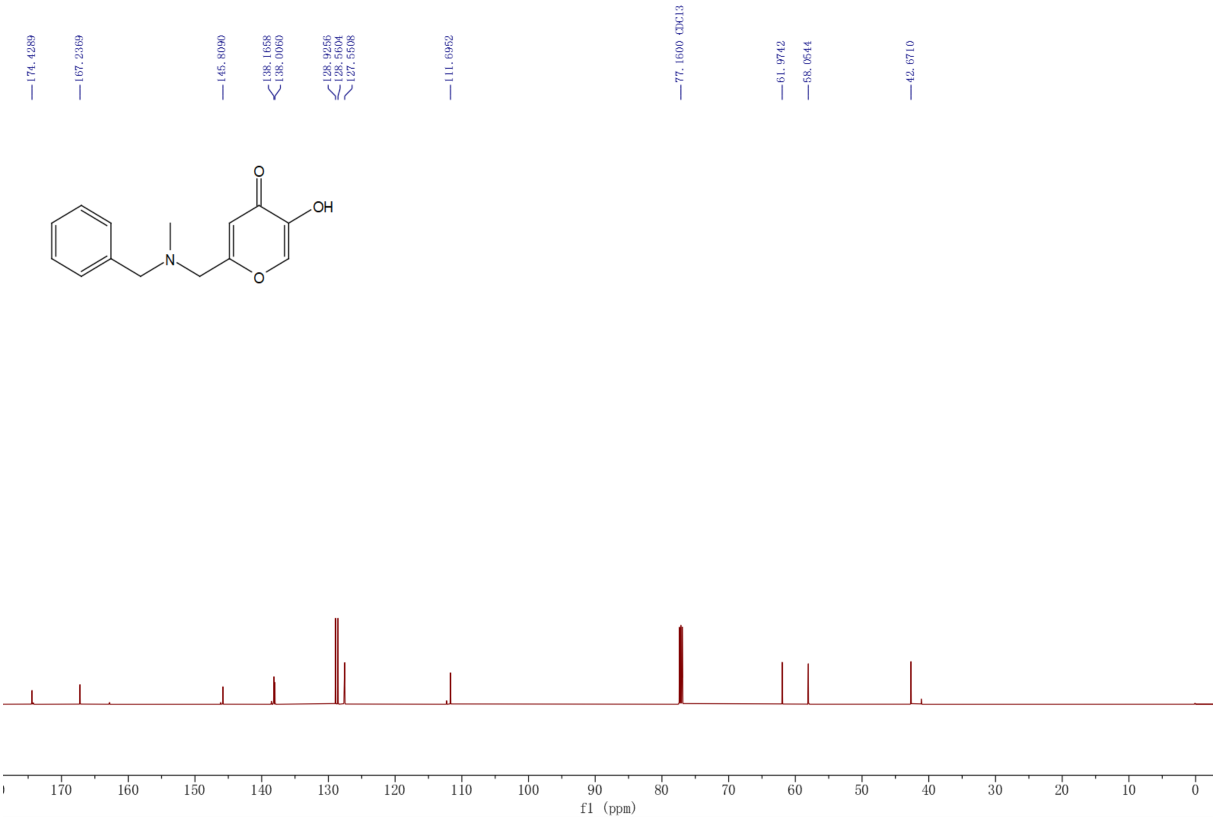
174.4289
167.2369
145.8090
138.1658
138.0060
128.9256
128.5604
127.5508
111.6952
77.1600 CDCl3
61.9742
58.0544
42.6710
O
OH
N
O
170
160
150
140
130
120
110
100
90
80
70
60
50
40
30
20
10
0
f1 (ppm)

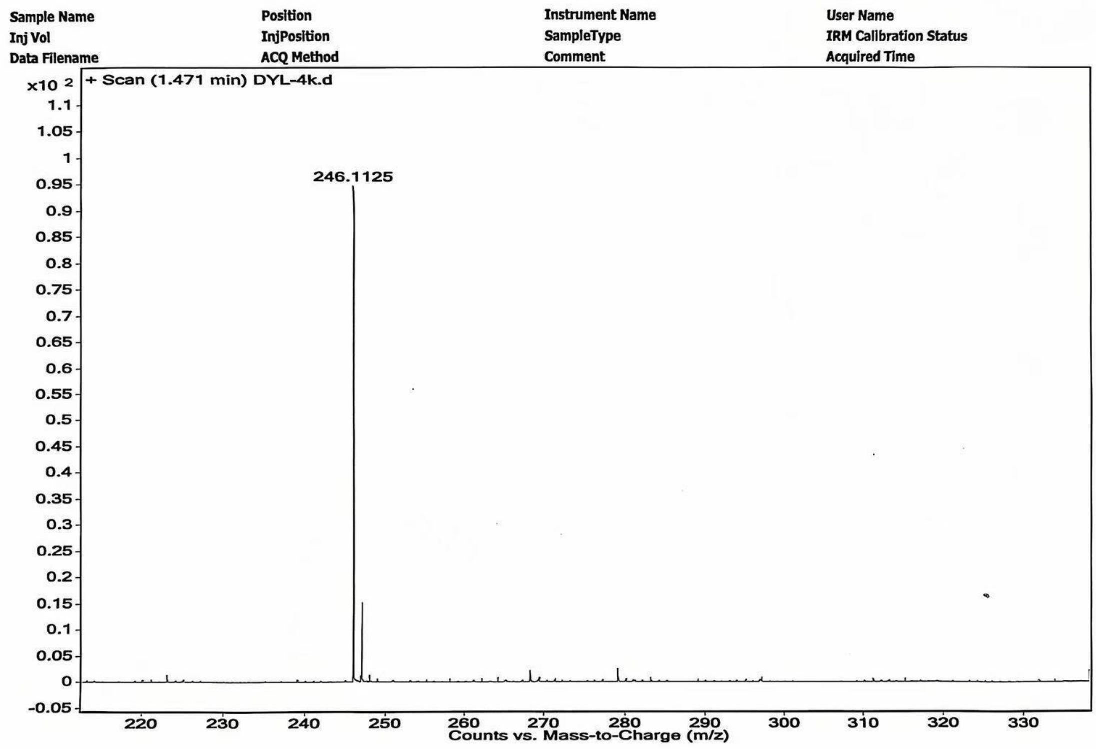

Figure S11. ^{1}H-NMR, ^{13}C-NMR and HRMS (ESI) spectra of compound **4k**.

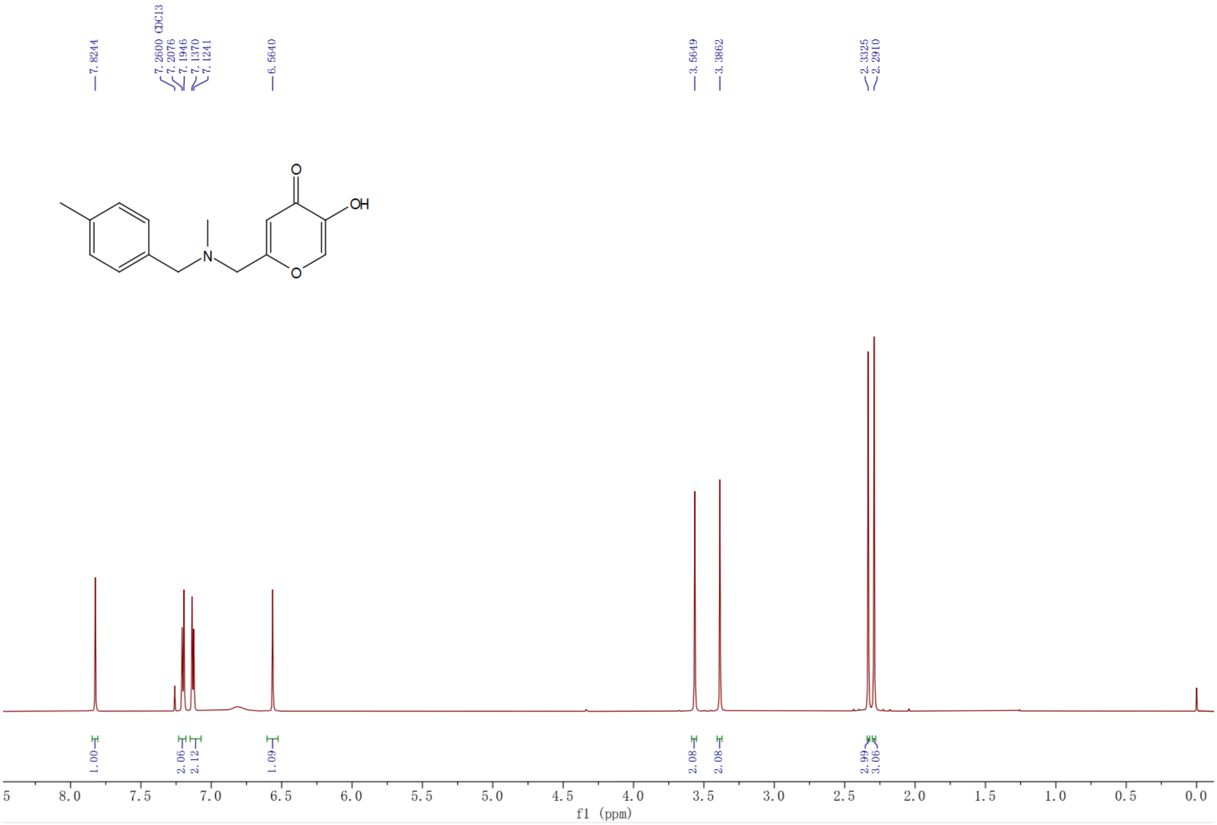
7.8244
7.2600 CDCl3
7.2076
7.1946
7.1370
7.1241
6.5640
3.5649
3.3862
2.3325
2.2910
O
OH
N
O
1.00
2.06
2.12
1.09
2.08
2.08
2.99
3.06
f1 (ppm)

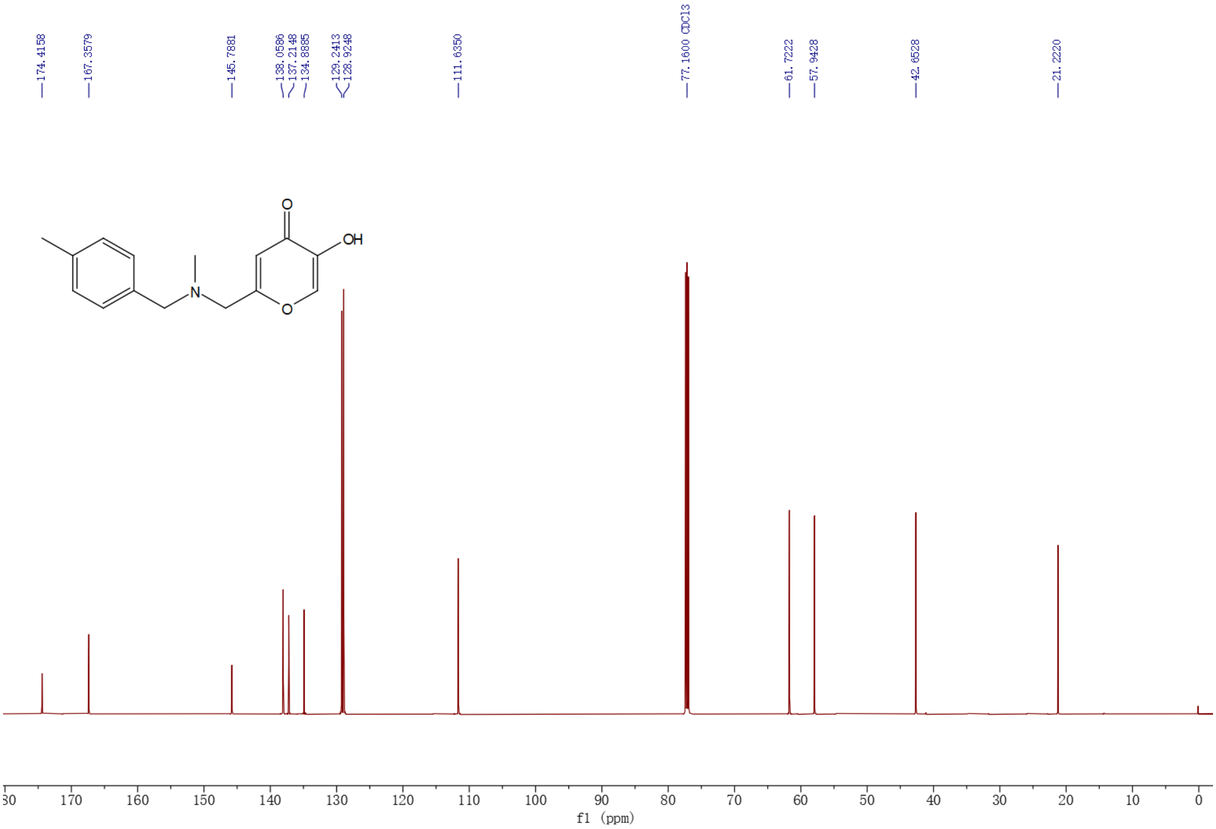
174.4158
167.3579
145.7881
138.0586
137.2148
134.8885
129.2413
128.9248
111.6350
77.1600 CDCl3
61.7222
57.9428
42.6528
21.2220
O
OH
N
O
f1 (ppm)

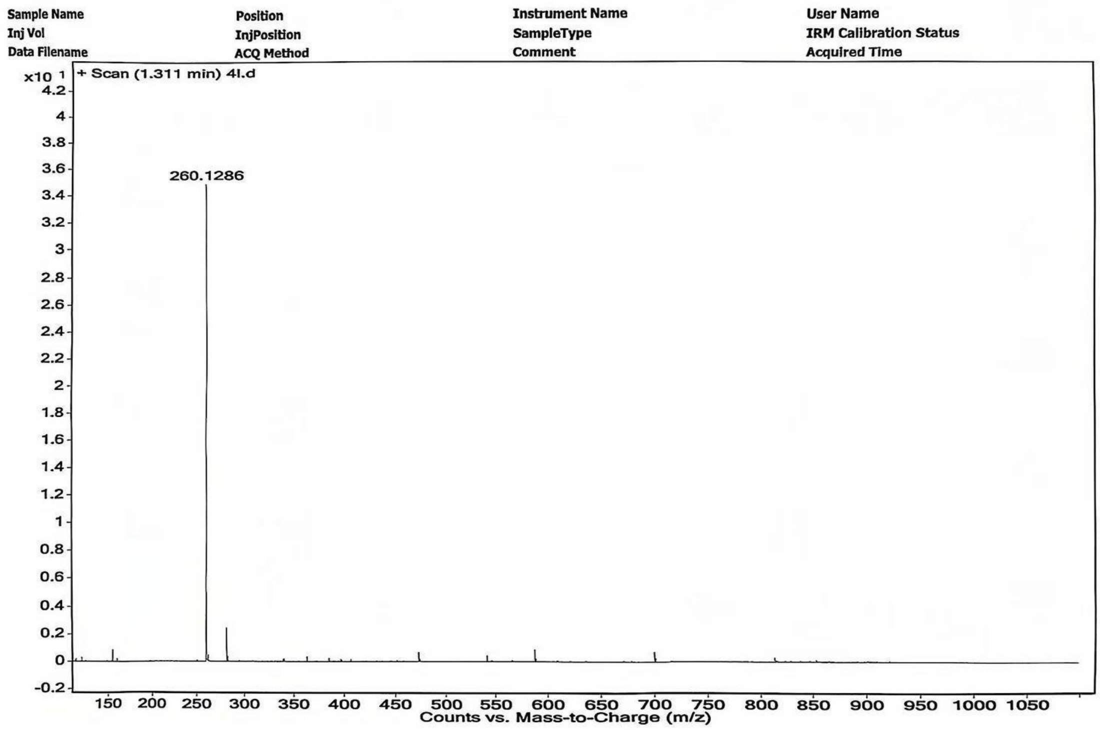

Figure S12. ^{1}H-NMR, ^{13}C-NMR and HRMS (ESI) spectra of compound **4l**.

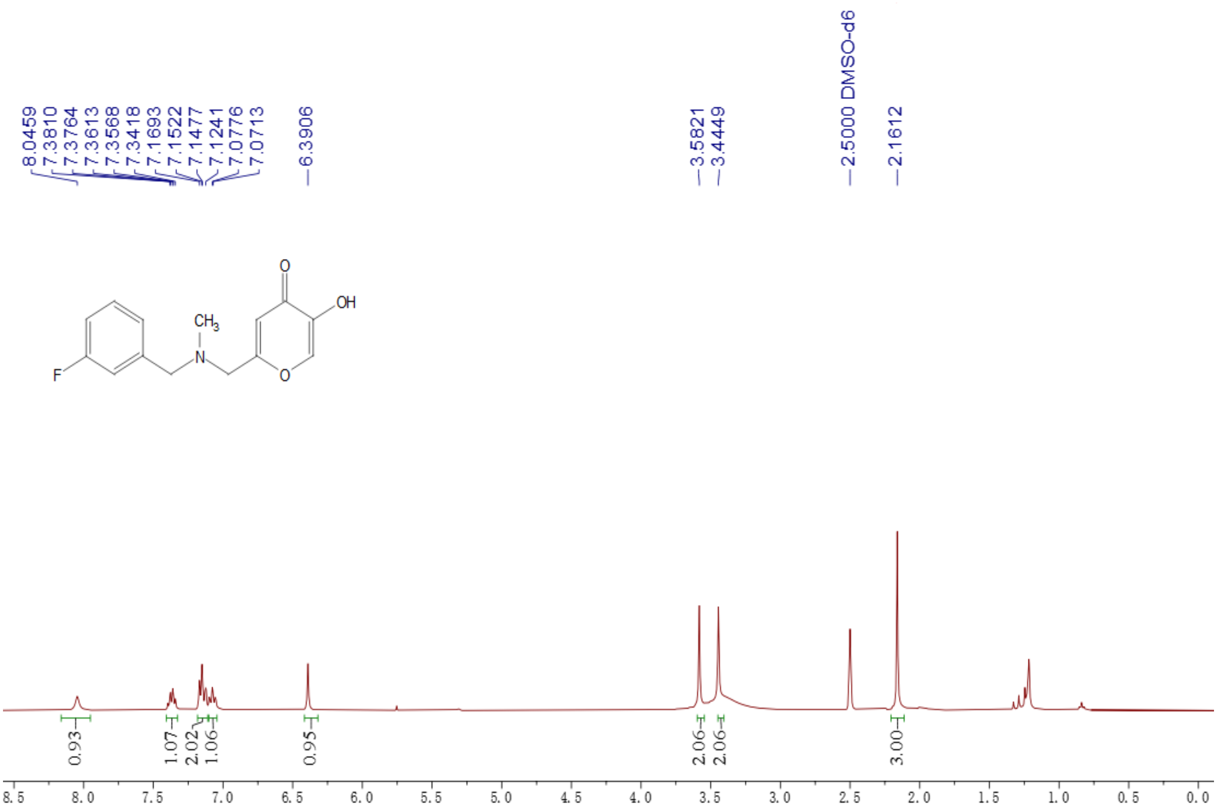
8.0459
7.3810
7.3764
7.3613
7.3568
7.3418
7.1693
7.1522
7.1477
7.1241
7.0776
7.0713
6.3906
3.5821
3.4449
2.5000 DMSO-d6
2.1612
O
OH
CH3
N
F
O
0.93
1.07
2.02
1.06
0.95
2.06
2.06
3.00

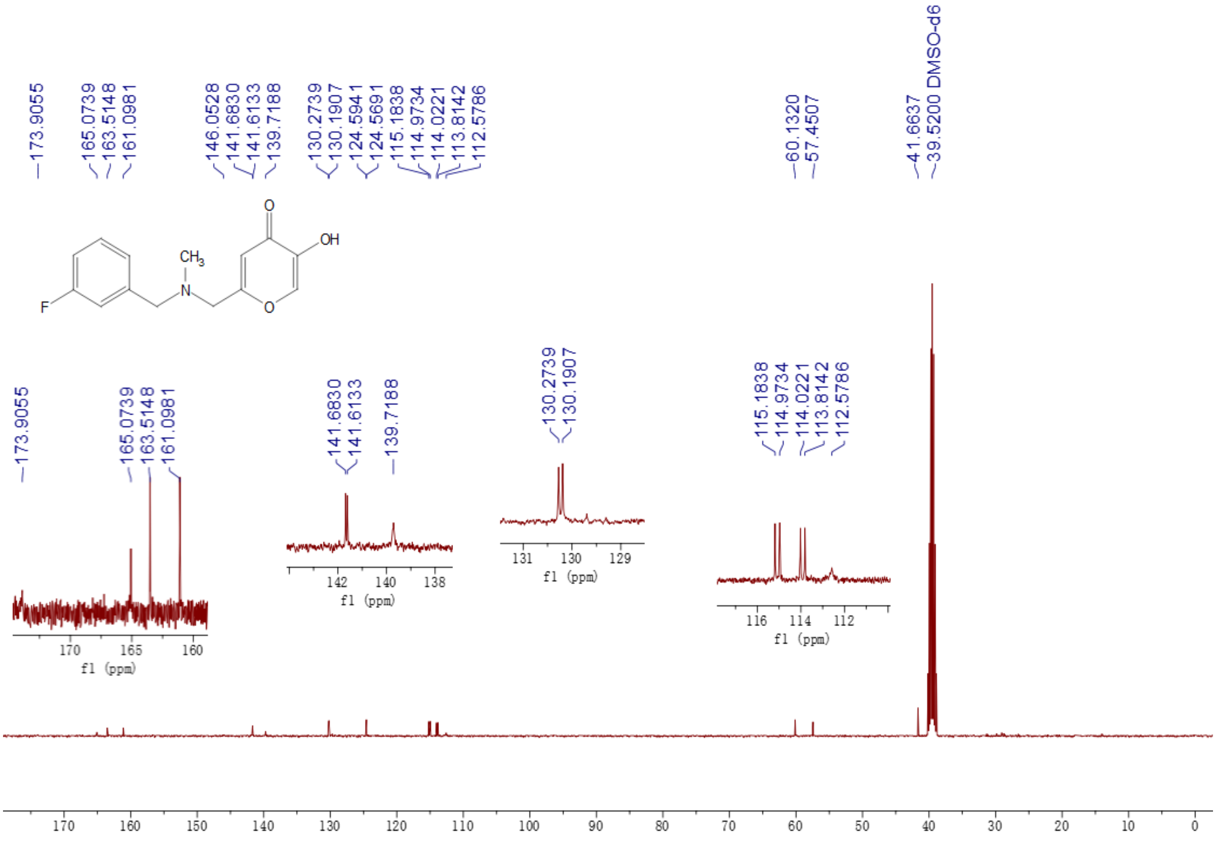
173.9055
165.0739
163.5148
161.0981
146.0528
141.6830
141.6133
139.7188
130.2739
130.1907
124.5941
124.5691
115.1838
114.9734
114.0221
113.8142
112.5786
60.1320
57.4507
41.6637
39.5200 DMSO-d6
O
OH
CH3
N
F
O
f1 (ppm)

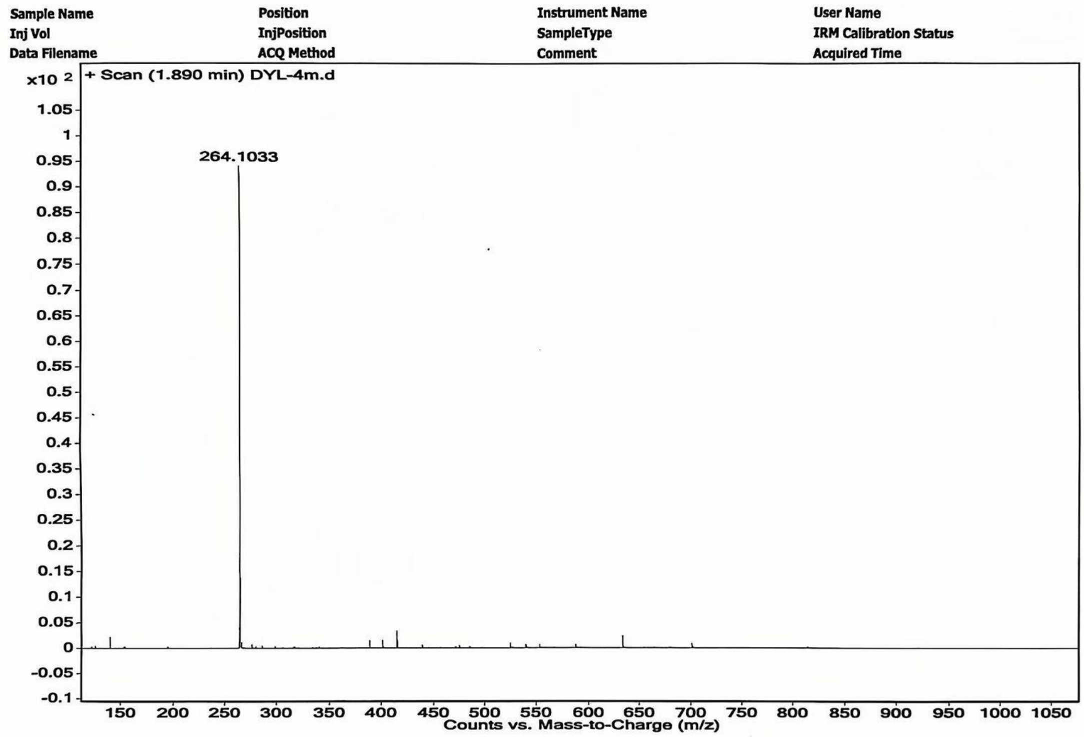

Figure S13. ^{1}H-NMR, ^{13}C-NMR and HRMS (ESI) spectra of compound **4m**.

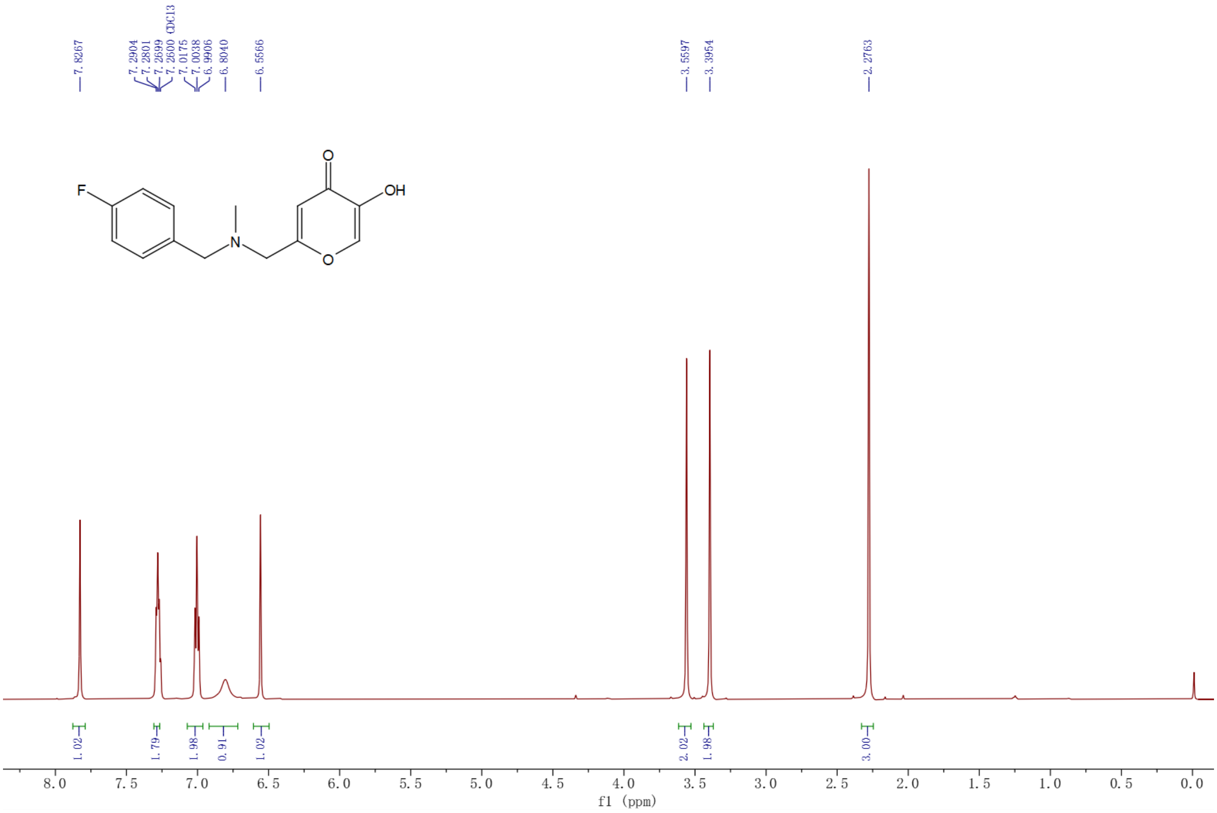
7.8267
7.2904
7.2801
7.2699
7.2600 CDCl3
7.0175
7.0038
6.9906
6.8040
6.5566
3.5597
3.3954
2.2763
F
O
OH
N
O
1.02
1.79
1.98
0.91
1.02
2.02
1.98
3.00
f1 (ppm)

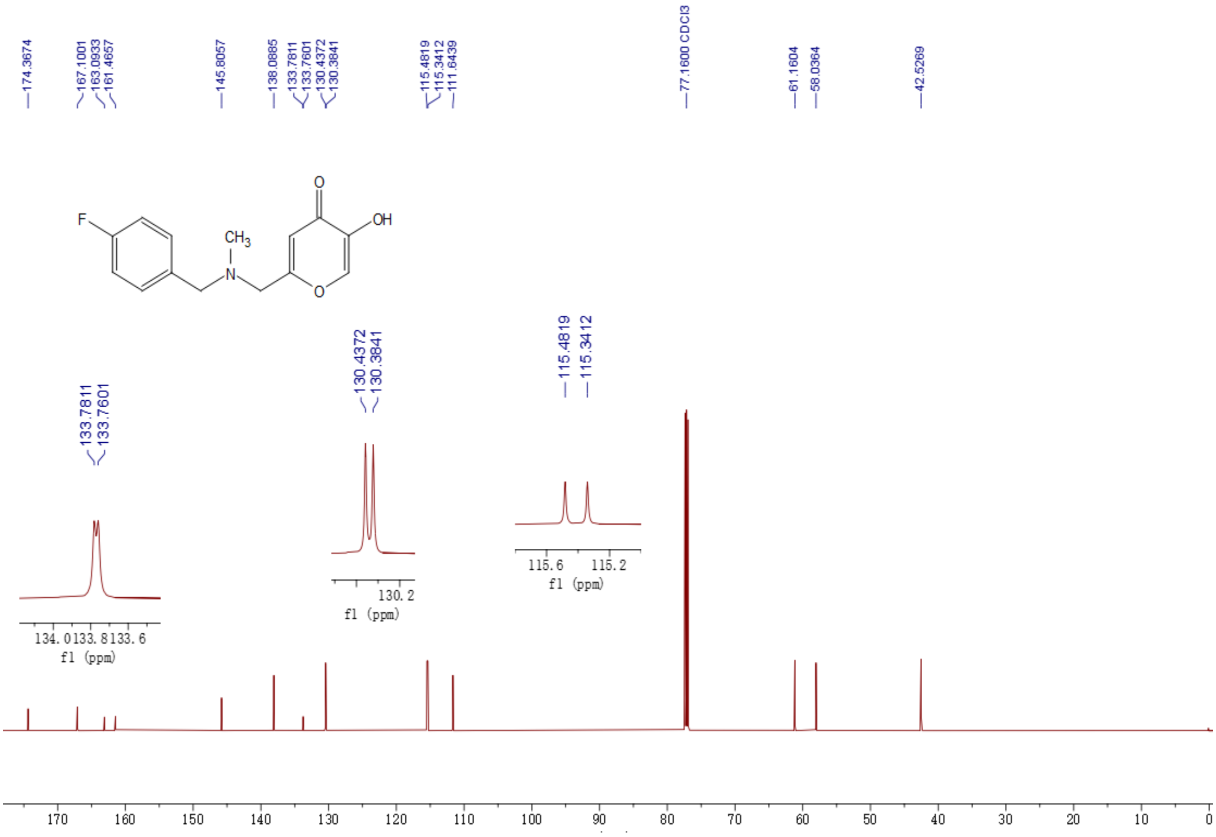
174.3674
167.1001
163.0933
161.4657
145.8057
138.0885
133.7811
133.7601
130.4372
130.3841
115.4819
115.3412
111.6439
77.1600 CDCl3
61.1604
58.0364
42.5269
F
CH3
N
O
OH
O
f1 (ppm)

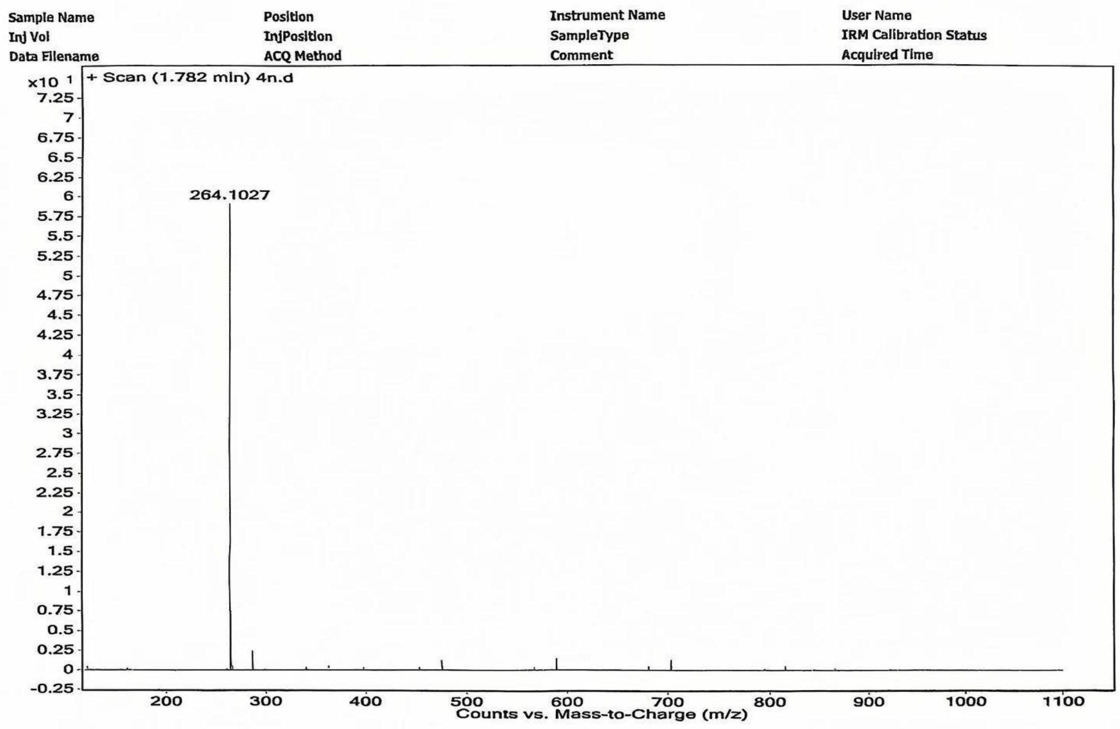

Figure S14. ^{1}H-NMR, ^{13}C-NMR and HRMS (ESI) spectra of compound **4n**.

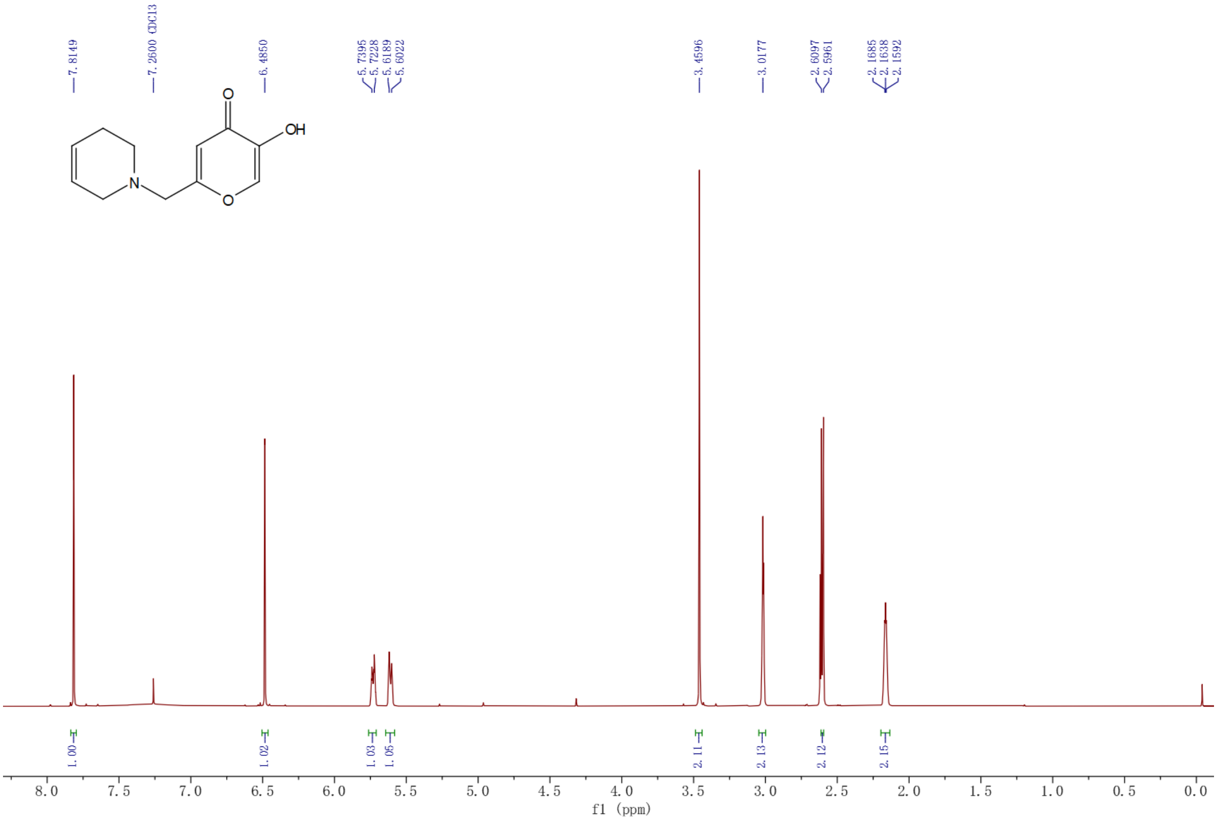
7.8149
7.2600 CDCl3
6.4850
5.7395
5.7228
5.6189
5.6022
3.4596
3.0177
2.6097
2.5961
2.1685
2.1638
2.1592
O
OH
N
O
1.00
1.02
1.03
1.05
2.11
2.13
2.12
2.15
8.0 7.5 7.0 6.5 6.0 5.5 5.0 4.5 4.0 3.5 3.0 2.5 2.0 1.5 1.0 0.5 0.0
f1 (ppm)

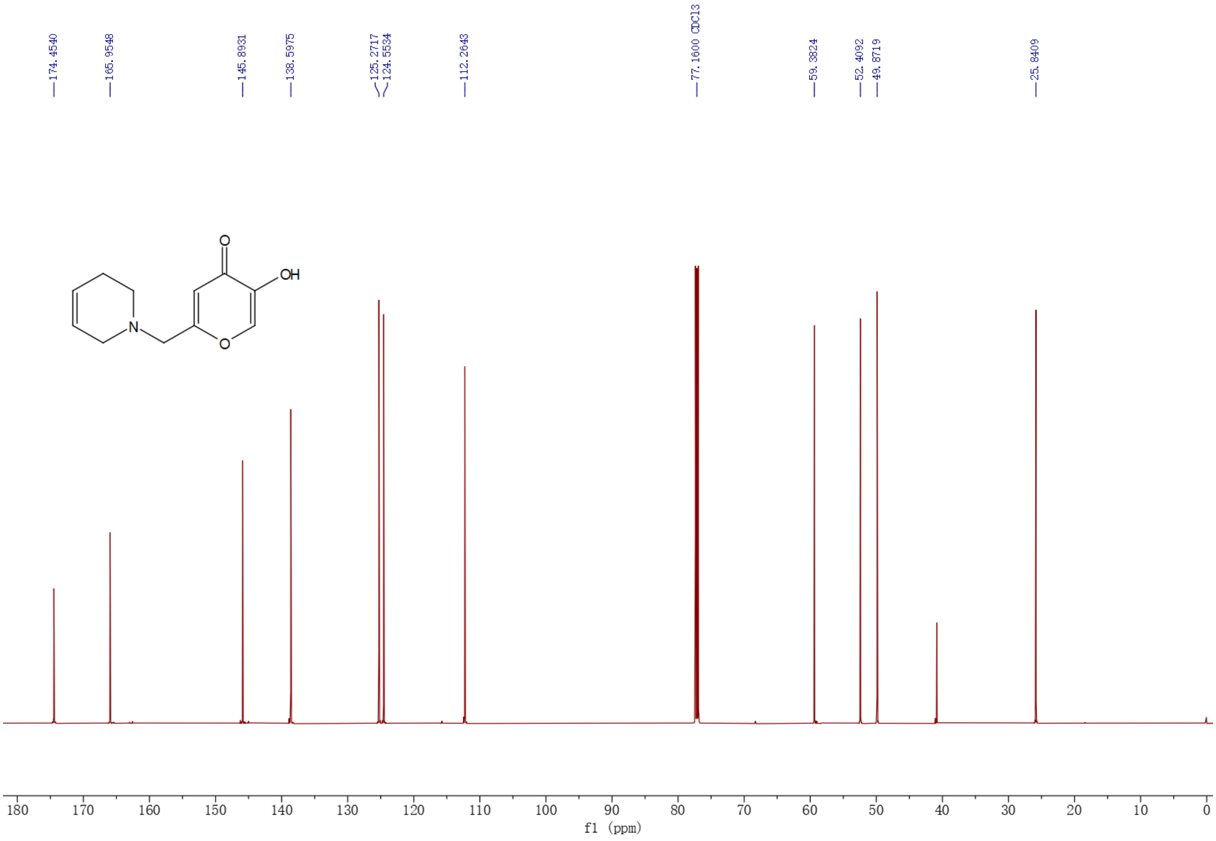
174.4540
165.9548
145.8931
138.5975
125.2717
124.5534
112.2643
77.1600 CDCl3
59.3824
52.4092
49.8719
25.8409
O
OH
N
O
180 170 160 150 140 130 120 110 100 90 80 70 60 50 40 30 20 10 0
f1 (ppm)

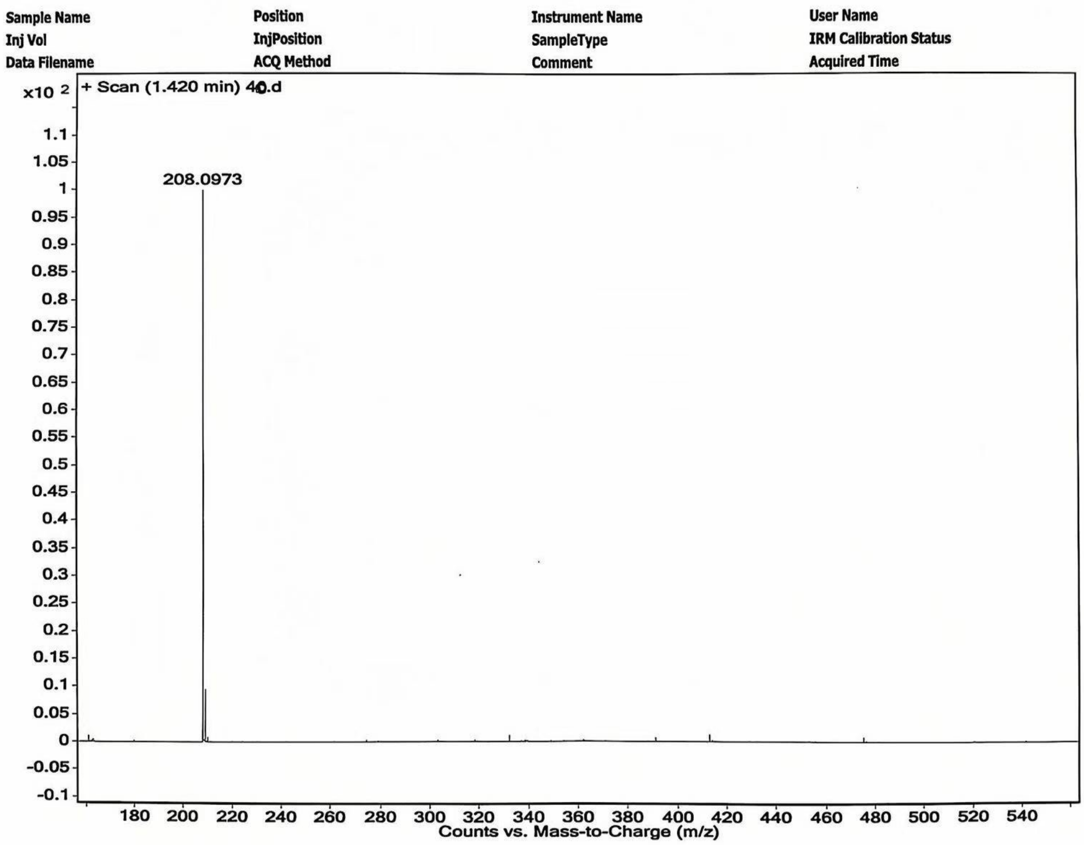

Figure S15. ^{1}H-NMR, ^{13}C-NMR and HRMS (ESI) spectra of compound **4o**.

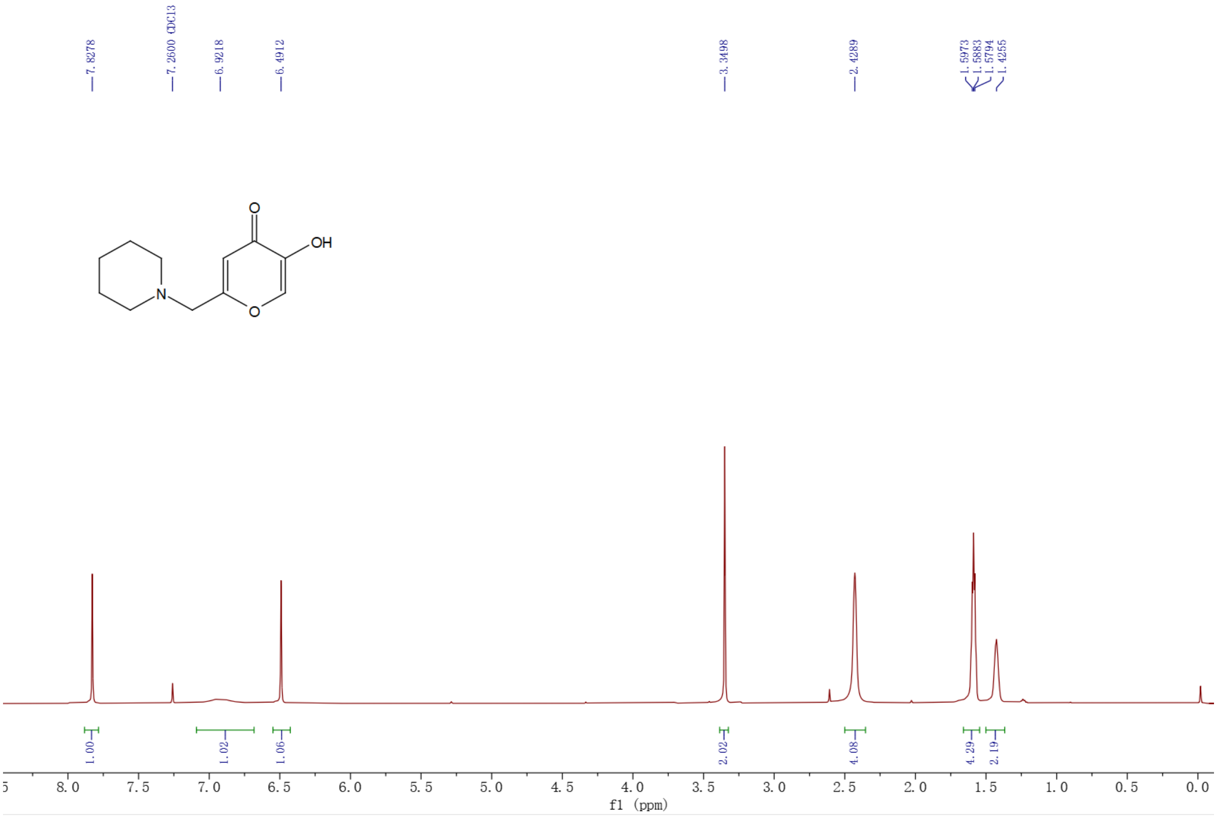
7.8278
7.2600 CDCl3
6.9218
6.4912
3.3498
2.4289
1.5973
1.5883
1.5794
1.4255
O
OH
N
O
1.00
1.02
1.06
2.02
4.08
4.29
2.19
8.0 7.5 7.0 6.5 6.0 5.5 5.0 4.5 4.0 3.5 3.0 2.5 2.0 1.5 1.0 0.5 0.0
f1 (ppm)

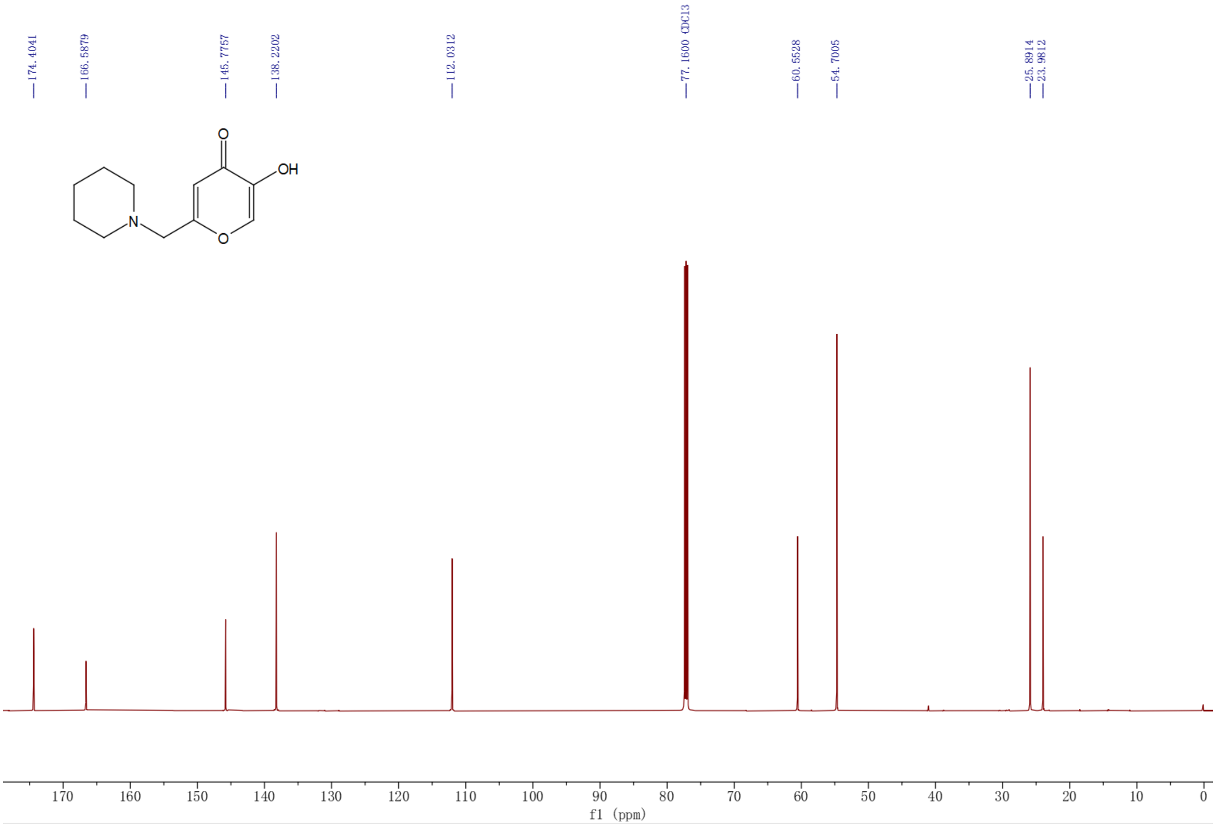
174.4041
166.5879
145.7757
138.2202
112.0312
77.1600 CDCl3
60.5528
54.7005
25.8914
23.9812
O
OH
N
O
170 160 150 140 130 120 110 100 90 80 70 60 50 40 30 20 10 0
f1 (ppm)

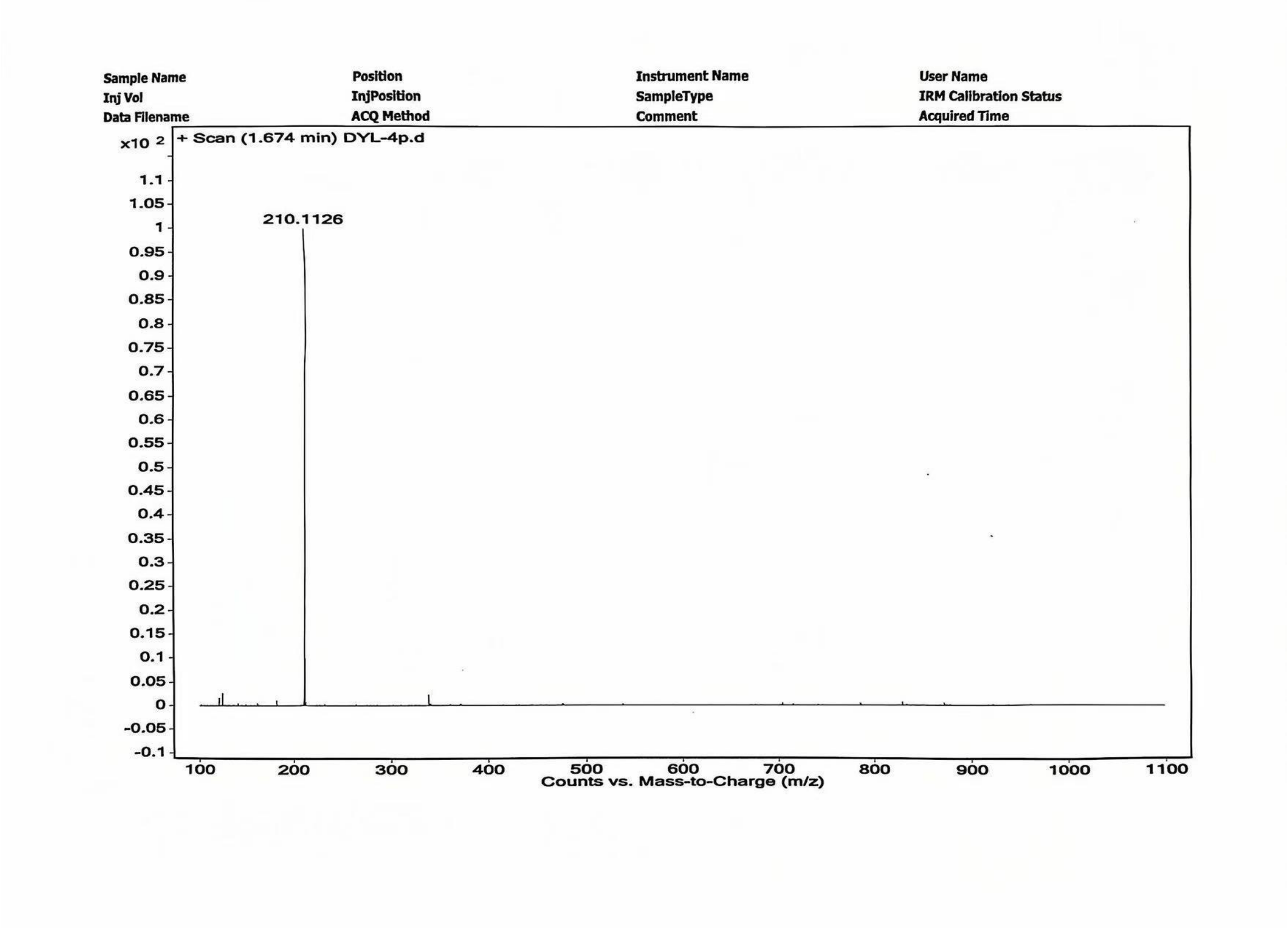

Figure S16. ^{1}H-NMR, ^{13}C-NMR and HRMS (ESI) spectra of compound **4p**.
